# Harmicens, Novel Harmine and Ferrocene Hybrids: Design, Synthesis and Biological Activity

**DOI:** 10.3390/ijms23169315

**Published:** 2022-08-18

**Authors:** Goran Poje, Marina Marinović, Kristina Pavić, Marija Mioč, Marijeta Kralj, Lais Pessanha de Carvalho, Jana Held, Ivana Perković, Zrinka Rajić

**Affiliations:** 1 Faculty of Pharmacy and Biochemistry, University of Zagreb, 10 000 Zagreb, Croatia; 2 Laboratory of Experimental Therapy, Division of Molecular Medicine, Ruder Boškovic Institute, 10 000 Zagreb, Croatia; 3 Institute of Tropical Medicine, University of Tübingen, 72074 Tübingen, Germany; 4 German Center for Infection Research (DZIF), Partner Site Tübingen, 72074 Tübingen, Germany

**Keywords:** β-carboline, harmine, ferrocene, hybrid, amide, triazole, synthesis, antiplasmodial, antiproliferative

## Abstract

Cancer and malaria are both global health threats. Due to the increase in the resistance to the known drugs, research on new active substances is a priority. Here, we present the design, synthesis, and evaluation of the biological activity of harmicens, hybrids composed of covalently bound harmine/β-carboline and ferrocene scaffolds. Structural diversity was achieved by varying the type and length of the linker between the β-carboline ring and ferrocene, as well as its position on the β-carboline ring. Triazole-type harmicens were prepared using Cu(I)-catalyzed azide-alkyne cycloaddition, while the synthesis of amide-type harmicens was carried out by applying a standard coupling reaction. The results of in vitro biological assays showed that the harmicens exerted moderate antiplasmodial activity against the erythrocytic stage of *P. falciparum* (IC_50_ in submicromolar and low micromolar range) and significant and selective antiproliferative activity against the MCF-7 and HCT116 cell lines (IC_50_ in the single-digit micromolar range, SI > 5.9). Cell localization experiments showed different localizations of nonselective harmicene **36** and HCT116-selective compound **28**, which clearly entered the nucleus. A cell cycle analysis revealed that selective harmicene **28** had already induced G1 cell cycle arrest after 24 h, followed by G2/M arrest with a concomitant drastic reduction in the percentage of cells in the S phase, whereas the effect of nonselective compound **36** on the cell cycle was much less pronounced, which agreed with their different localizations within the cell.

## 1. Introduction

Cancer remains one of the leading causes of mortality worldwide, as it was responsible for nearly 10 million deaths in 2020 [1]. Unfortunately, these numbers are expected to rise in the future [2,3]. Current cancer treatment still faces many challenges, such as low specificity and high toxicity of the available drugs, drug resistance, and high cost of the targeted therapy [4]. Malaria, an infectious disease caused by a protozoan parasite of the genus *Plasmodium*, is another serious global health threat [5]. The deadliest form of the disease is caused by the most prevalent and drug-resistant species, *P. falciparum* [6]. Unfortunately, the COVID-19 outbreak interrupted the steady decline in both malaria cases and deaths since 2000, resulting in an increase of 13 million malaria cases and 69,000 deaths in 2020 compared to 2019 [7]. Therefore, the discovery of new anticancer and antimalarial agents is a top priority.

Due to the complexity of cancer pathophysiology and the complex life cycle of the malaria parasite, a combination of drugs is a standard treatment option and could provide protection against drug resistance. However, complications such as drug–drug interactions and lower treatment adherence may also occur. On the other hand, the molecular hybridization approach is a rational strategy for the synthesis of new bioactive compounds. In this approach, a covalent bond is established between two or more pharmacophores to obtain hybrid compounds that have the potential to bridge the shortcomings of combination therapy [8,9,10]. 

β-carbolines, which are naturally occurring or synthetic compounds with a tricyclic pyrido[3,4-*b*]indole ring in their structure, have been extensively studied and have shown a wide range of biological activities, such as anticancer, antimalarial, antiviral, antibacterial, neuropharmacological, and antithrombotic activities [11]. Harmine, isolated from *Peganum harmala*, and its numerous analogues have shown significant cytotoxic activity against human cancer cell lines in the low micromolar and sub-micromolar range [12,13,14,15,16]. Several reviews addressed the anticancer activity of β-carboline derivatives and proposed different modes of action [9,15,17]. Similarly, the antimalarial activity of harmine and other β-carboline derivatives is also well-documented [18,19,20]. On the other hand, ferrocene is an important compound in bioinorganometallic chemistry due to its high stability, favorable redox properties, and low toxicity [21]. Furthermore, extensive research has shown that the incorporation of ferrocene in the structure of known anticancer or antimalarial agents improved activity [4,22,23,24,25,26,27]. The best-known examples are ferrocifen and ferroquine, ferrocene–tamoxifen or chloroquine hybrids [4]. Ferroquine, the first organometallic drug candidate, showed potent and significant antiplasmodial activity against CQ-resistant strains of *P. falciparum* and is in the clinical trials for the treatment of malaria [28,29]. Interestingly, ferroquine also demonstrated potential as an anticancer drug [30].

**Figure 1:**
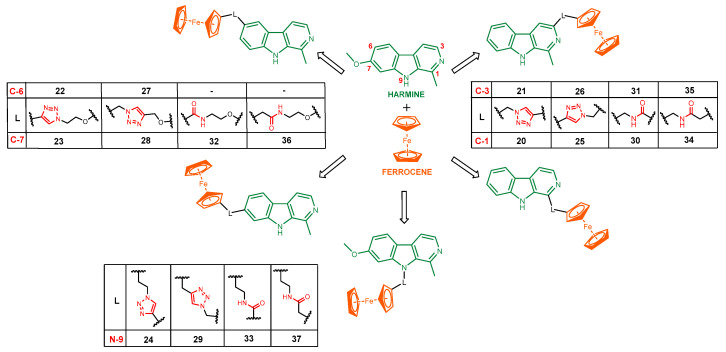
Harmicens—new harmine–ferrocene hybrids (harmine/β-carboline is marked in green and ferrocene is marked in red).

Our previous work on harmine hybrids, i.e., harmicines, harmirins, and harmiquins, demonstrated that the development of hybrids is indeed a valid strategy for obtaining biologically active compounds that exhibit more pronounced activity than the parent compounds [31,32,33,34,35]. Encouraged by the literature data, which clearly show the potential of organometallic compounds as anticancer and antimalarial therapeutics [36,37,38,39,40,41], we set out to prepare harmicens—hybrids in which ferrocene is covalently bound to harmine/β-carboline (Figure 1), and to investigate their antiproliferative and antiplasmodial activities.

## 2. Results and Discussion

### 2.1. Chemistry

In this paper, we report the synthesis of hybrid compounds composed of harmine/β-carboline and ferrocene covalently bound via a triazole ring or an amide bond, leading to the triazole- (TT, **20**–**24** and **25**–**29**) or amide-type (AT, **30**–**33** and **34**–**37**) harmicens, respectively. Additional structural diversity was achieved by changing the position and length of the linker between the β-carboline ring and ferrocene. Thus, harmicens were prepared at five positions of the β-carboline ring, namely C-1, C-3, O-6, O-7, and N-9. Within both series of harmicens, compounds **20**–**24** and **30**–**33** contain either a triazole or an amide bond directly linked to ferrocene, in contrast to compounds **25**–**29** and **34**–**37**, which have a methylene bridge between the triazole/amide and ferrocene (Figure 1).

**Scheme 1:**
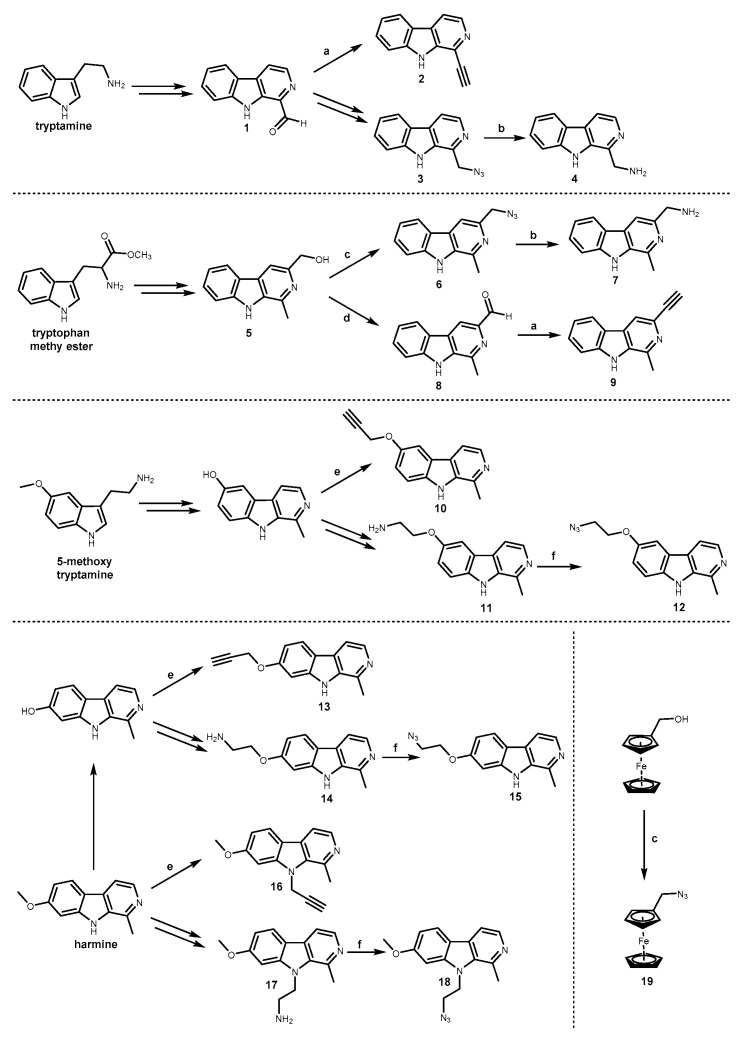
Synthesis of β-carboline and ferrocene intermediates **1**–**19**. Reagents and conditions: (a) Bestmann–Ohira reagent, K_2_CO_3_, MeOH, rt; (b) H_2_/Pd/C, MeOH, rt; (c) ADMP, DBU, THF, 0 °C; (d) MnO_2_, THF, rt; (e) HC≡CCH_2_Br, Cs_2_CO_3_, DMF, rt; (f) ISA × HCl, K_2_CO_3_, CuSO_4_ × 5H_2_O, MeOH, rt.

TT harmicens were prepared using Cu(I)-catalyzed azide-alkyne cycloaddition (CuAAC), known as “click” chemistry, while the synthesis of AT harmicens was performed using a standard coupling reaction. Since both methods required starting building blocks based on harmine and ferrocene motifs, i.e., azides and alkynes or amines and carboxylic acids, extensive synthetic work was performed prior to the synthesis of the title compounds. In our previous work, we synthesized azides at the C-1 and C-3 (**3** and **6**), alkynes at the O-6, O-7, and N-9 (**10**, **13**, and **16**), and amines at the C-1, C-3, O-6, O-7, and N-9 positions of the β-carboline ring (**4**, **7**, **11**, **14**, and **17**) [31,32,33,35]. Here, we present the synthesis of alkynes at the C-1 and C-3 (**2**, **9**) and azides at the O-6, O-7, and N-9 (**12**, **15,** and **18**) positions of the β-carboline ring. We also prepared the required azide-based ferrocene **19**. Scheme 1 shows the simplified synthetic routes to the aforementioned intermediates.

**Scheme 2:**
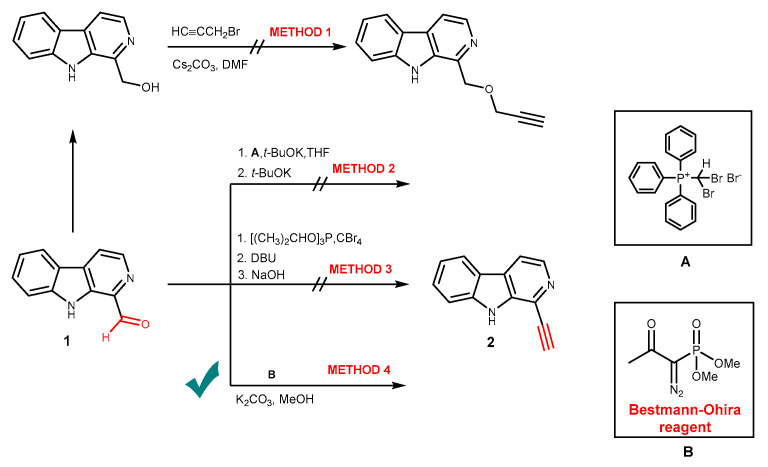
Optimization of the alkyne **2** synthesis.

The synthesis of alkynes **2** and **9** proved to be the most challenging. We tried several synthetic approaches, as shown in Scheme 2, for the synthesis of alkyne at the position C-1. The selective alkylation of the corresponding alcohol with propargyl bromide in the presence of Cs_2_CO_3_ resulted in a complete alkylation of β-carboline at position N-9.

The second attempt was made using aldehyde-to-alkyne homologation (Methods 2–4). Methods 2 and 3 implied the use of strong bases (t-BuOK, DBU, and NaOH), required complex work-up [42], and the reactions resulted in a mixture of products. Method 4 represents the Bestmann–Ohira modification of the Seyferth–Gilbert reaction. Classical Seyferth–Gilbert homologation is a one-pot reaction in which aldehyde is converted to the corresponding alkyne in the presence of dimethyl (diazomethyl)phosphonate (DAMP, the Seyferth–Gilbert reagent). This reagent is not commercially available due to its instability, and requires preparation before the reaction is carried out. The Bestmann–Ohira procedure uses a more stable dimethyl (1-diazo-2-oxopropyl)phosphonate (Bestmann–Ohira reagent (BOR); reagent B, Scheme 2) that is converted to DAMP in situ. This reaction can be successfully employed for the preparation of a wide range of terminal alkynes with good to excellent yields under mild reaction conditions (strong bases, low temperatures, and inert gas techniques are not necessary) following a simple work-up [42]. To our delight and after careful optimization of the reaction conditions, **2** and **9** were successfully prepared from the corresponding aldehydes **1** and **8** by applying Method 4.

On the other hand, amines **11**, **14**, and **17** were easily and effectively converted to azides **12**, **15**, and **18**, respectively, with imidazole-1-sulfonyl azide hydrochloride (ISA × HCl), a diazotransfer reagent, in the presence of K_2_CO_3_ and CuSO_4_ × 5H_2_O. ISA × HCl is an efficient, shelf-stable crystalline crude reagent that can be prepared in a one-pot reaction from inexpensive materials (imidazole, NaN_3_, and SO_2_Cl_2_) [43].

**Scheme 3:**
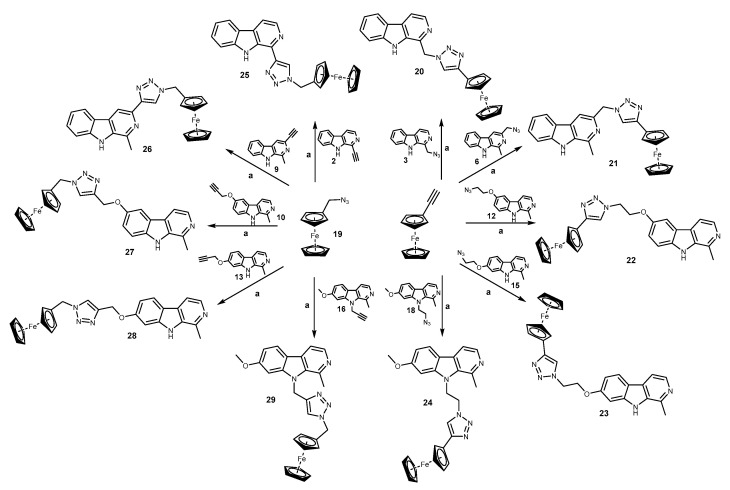
Synthesis of TT harmicens **20**–**29**. Reagents and conditions: (a) Na ascorbate, CuSO_4_ × 5H_2_O, DMF/H_2_O, rt.

TT harmicens **20**–**24** were prepared from ethynylferrocene and β-carboline azides **3**, **6**, **12**, **15**, and **18**, whereas the synthesis of TT harmicens **25**–**29** was carried out via a “click” reaction between (azidomethyl)ferrocene **19** and β-carboline alkynes **2**, **9**, **10**, **13**, and **16** (Scheme 3). The use of copper(II) acetate in methanol at room temperature resulted in a poor reaction performance and a large number of side products. Since we were not satisfied with the reaction outcome, we decided to investigate whether changing the reaction conditions would increase the reaction yield, decrease the number of side products, and simplify the work-up. Heating the reaction mixture in a microwave reactor (50 °C, 1 h) had no positive effect on the progress of the reaction or the purity of the products. Reactions carried out in the presence of sodium ascorbate and CuSO_4_ × 5H_2_O in a mixture of tert-butanol and water (1:1 ratio) at room temperature progressed well, but the selectivity was low again. Changing the solvent to DMF and water (2:1 ratio) significantly reduced the number of side products and increased the reaction yield. Work-up included filtration of the crude product from the reaction mixture or solvent extraction and column chromatography with the addition of Al_2_O_3_ to remove the residual copper salts.

**Scheme 4:**
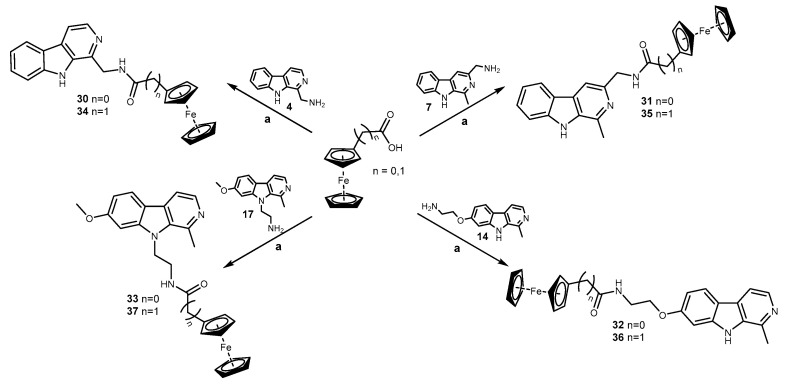
Synthesis of AT harmicens **30**–**37**. Reagents and conditions: (a) HATU, DIEA, CH_2_Cl_2_, rt.

The synthesis of harmicens **30**–**33** and **34**–**37** at positions C-1, C-3, O-7, and N-9 was successfully carried out via a standard coupling reaction (HATU, DIEA) using amines **4**, **7**, **14**, and **17** and ferrocenecarboxylic or ferroceneacetic acid (Scheme 4). The carboxylic acids were added in small excess (1.1 equivalents) to increase the reaction yield. Unfortunately, the preparation of AT harmicene at position 6 of the β-carboline ring was not successful. Attempts to optimize the reaction conditions and to carry out the synthesis via other intermediates did not afford the desired results.

Harmicens were characterized by standard methods (MS, IR, ^1^H and ^13^C NMR), whereas their purity was checked by elemental analysis and HPLC. The data obtained are given in the Materials and Methods section and in the Supplementary Material (Tables S1–S11). 

### 2.2. Biological Activity

Our further work was focused on the evaluation of harmicens’ antiplasmodial and antiproliferative activities against the erythrocytic stages of the *Plasmodium* life cycle and a panel of human cell lines, respectively.

#### 2.2.1. In Vitro Antiplasmodial Activity

The in vitro activity of harmicens **20**–**37** against the erythrocytic stages of CQ-sensitive (*Pf*3D7) and multidrug-resistant (*Pf*Dd2) *P. falciparum* strains was evaluated following a previously described method (Table 1) [44,45,46]. The results showed that the antiplasmodial activity of the prepared harmicens was moderate (IC_50_ in submicromolar and micromolar range). A detailed analysis revealed that most of the TT and AT harmicens exhibited improved antiplasmodial activity over the parent compound harmine against *Pf*3D7. Notably, 7/18 compounds exerted activity that was an order of magnitude stronger. All harmicens had markedly better antiplasmodial activity against *Pf*Dd2 than harmine, which was not active at all. Unfortunately, their activity against both strains was lower than the activity of the reference drug, chloroquine.

A comparison of the harmicens’ activity against two *P. falciparum* strains showed that *Pf*3D7 was generally more sensitive than *Pf*Dd2, as 7/18 compounds were active at submicromolar concentrations, whereas only 2/18 harmicens had submicromolar activity against *Pf*Dd2. Among TT harmicens, hybrids in which the β-carboline ring was substituted at O-6 and O-7 showed activity at submicromolar concentrations. On the other hand, AT harmicens substituted at O-7 and N-9 showed better activity. The length of the linker also influenced the activity. Interestingly, the highest activity against *Pf*3D7 was exerted by TT harmicene **23**, bearing a triazole directly linked to ferrocene (IC_50_ = 0.15 ± 0.05 μM), whereas its counterpart, harmicene **28**, which had a methylene bridge between triazole and ferrocene, was inactive at the highest concentration tested, resulting in a 370-fold decrease in antiplasmodial activity. On the contrary, AT harmicens with a shorter amide linker generally showed stronger antiplasmodial activity against *Pf*Dd2 than their counterparts with a longer linker. The most active compound against *Pf*Dd2 was AT harmicene **32** (IC_50_ = 0.66 ± 0.01 μM). The effect of the linker remains a potentially useful parameter that should be further investigated.

#### 2.2.2. In Vitro Antiproliferative Activity

The antiproliferative activity of harmicens **20**–**37** was evaluated against a panel of human tumor cell lines (hepatocellular carcinoma—HepG2, colorectal adenocarcinoma, Dukes’ type C—SW620, colorectal carcinoma—HCT116, and breast adenocarcinoma—MCF-7) and a noncancer cell line (embryonic kidney—Hek293T) in vitro; the obtained results are shown in Table 2. The parent compound harmine, as well as the reference drug 5-fluorouracil, were used as positive controls. Initially, a prescreening was performed, and only the compounds that resulted in more than a 50% reduction in the mitochondrial metabolic activity at a concentration of 50 µM were selected for IC_50_ determination. Since the MCF-7 and HCT116 cell lines were the most sensitive to harmicens, we decided to calculate the selectivity index (SI) for each harmicene as the fractional ratio between the IC_50_ values for Hek293T and the tumor cell line MCF-7 or HCT116.

The results showed that harmicens exhibited significant antiproliferative activity against the tested tumor cell lines, especially against MCF-7, HCT116, and SW620 (7/18 compounds displayed activity in the low micromolar range), whereas HepG2 was the least sensitive cell line (3/18 compounds displayed a single-digit micromolar IC_50_). Remarkably, their activity against noncancer cell line Hek293T was much lower. Only the most active and nonselective compounds significantly affected Hek293T (**22** and **36**). Thus, the bridging of harmine and ferrocene led to selective compounds, which is a very important result.

TT harmicens showed stronger and more selective activity against the tumor cell lines than the AT harmicens. In particular, TT harmicens containing a methylene group between the triazole and ferrocene moieties, i.e., compounds **25**–**29**, showed the least activity against Hek293T and the strongest against the tumor cell lines. A more detailed analysis revealed that TT harmicens **21**, **25,** and **28** exhibited selective activity against HCT116 (SIs > 6), and AT harmicene **30** against MCF-7 (SI > 5.9). In addition, TT harmicens **26** and **27** displayed selective antiproliferative activity against SW620 and MCF-7, but not against HCT116. Remarkably, the activity of harmicens selective against MCF-7, **26**, **27**, and **30**, was ~3–6.5-fold stronger than the activity of 5-FU. The most active harmicene against MCF-7 was compound **26** (IC_50_ = 3.7 ± 0.2 μM, SI = 12.6). On the other hand, the activity of harmicens against HCT116 was similar to the activity of 5-FU, but, as mentioned earlier, was much more selective (IC_50_ (**28**) = 7.4 ± 0.4 μM, SI > 6.8).

A comparison of compounds prepared at the same position of the β-carboline ring revealed the following:

(1) C-1: Harmicens were either selective against HCT116 (TT harmicene **25**) or MCF-7 (AT harmicene **30**), or exerted very low activity/were inactive.
(2) C-3: Harmicens were mostly selective, especially TT harmicens **21** and **26**. Although AT harmicens had lower activity, a similar trend was observed.
(3) O-6: Harmicens exerted very strong activity against the tumor cell lines. Harmicene **27** was also selective against MCF-7 and SW620. Unfortunately, only TT harmicens were prepared, so comparison with AT harmicens was not possible.
(4) O-7: Harmicens showed pronounced and nonselective activity against all cell lines tested, with the exception of TT harmicene **28**.
(5) N-9: The activity of harmicens was moderate to low, and some degree of selectivity was observed against the HCT116 and MCF-7 cell lines.

#### 2.2.3. Cell Localization

To gain insight into the destiny of the compounds within the cell, we decided to investigate the cell localization of two compounds with low micromolar IC_50_ values: (1) compound **28,** which was cytotoxic only against HCT116; and (2) compound **36,** which showed cytotoxicity toward all tested cell lines. The aforementioned compounds were selected particularly to investigate whether there was a difference in the potential mode of action between the HCT116-selective and generally less selective compounds.

To examine the intracellular localization of compounds **28** and **36**, we took advantage of their intrinsic fluorescence properties. We incubated MCF-7 cells with compound **28** at two concentrations (10 and 50 µM), or with compound **36** at a 5 µM concentration for 30 min and 1 h and analyzed their distribution using fluorescence microscopy. We chose different concentrations and incubation periods to determine the optimal treatment protocol that would not cause cell damage and would result in high-contrast images. No autofluorescence was detected by examining untreated cells under typical imaging conditions. 

**Figure 2:**
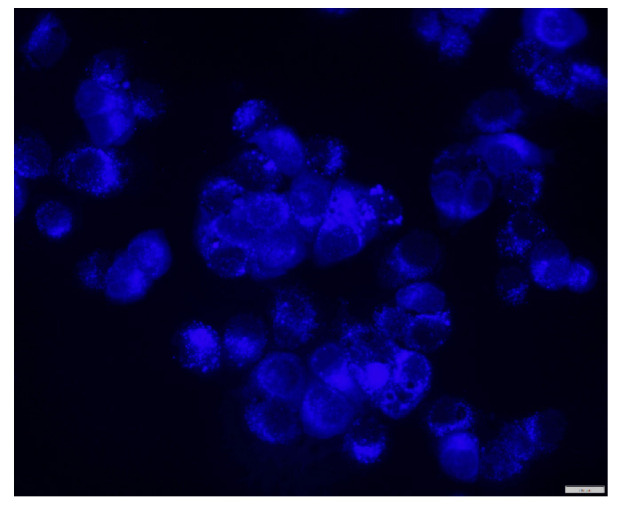
A fluorescence microscopy image (400× magnification) of MCF-7 cells incubated with 10 µM of compound **28** for 30 min showing both cytoplasmic and nuclear localization. The scale bar is 20 µm.

Harmicene **28** showed punctate, bright staining within the cytoplasm at a 10 µM concentration after 30 min, while nuclei also fluoresced, suggesting that compound **28** did enter the nucleus. Thus, compound **28** possibly targeted both nuclear DNA and cytoplasmic targets (Figure 2). This was contrary to our previous finding that harmine derivatives did not enter the cell nuclei [34]. A longer incubation period did not improve the fluorescence signal, while the 5-fold higher concentration disrupted the cell morphology (data not shown). On the other hand, compound **36** clearly showed different distribution within the cell in comparison with **28**. The staining within the cytoplasm appeared less bright, while the fluorescence of the nuclei was not as apparent. However, due to the technical limitations and spectral characteristics of the compound, we could not draw a definite conclusion on the cell distribution of **36** based on the obtained photographs (data shown in Figure S1 of the Supplementary Materials).

#### 2.2.4. Cell Cycle Analysis

**Figure 3:**
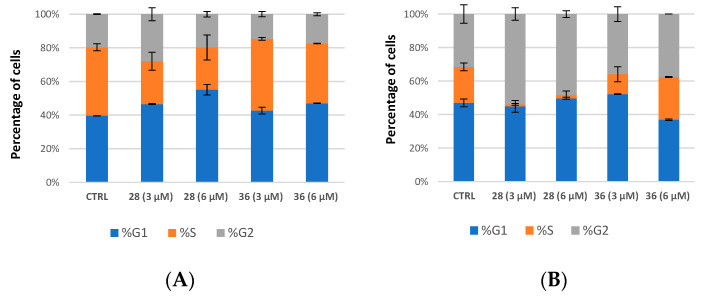
The effect of **28** and **36** on the cell cycle distribution of HCT116 cells. Cells were treated with 3 µM and 6 µM of each compound for 24 h (**A**) and 48 h (**B**), and then the cell cycle was analyzed using flow cytometry. The histograms represent the percentage of cells in the respective cell cycle phase (G1, S, and G2/M). Average values from duplicates from one representative experiment ± SD are presented.

As cell localization experiments showed different distributions of compounds **28** and **36** within the cell, we decided to assess their influences on the cell cycle. To that end, the cells were treated for 24 and 48 h with 3 µM and 6 µM concentrations of each compound, which corresponded to 0.5 × IC_50_ and IC_50_ values obtained in the MTT assay after 72 h, respectively. Afterward, the DNA was stained with propidium iodide and subjected to flow cytometry analysis. The cell cycle distribution is shown in Figure 3.

The treatment of cells with compound **28** resulted in a statistically significant increase in cells in the G1 phase at both tested concentrations already after 24 h, while compound **36** caused G1 phase arrest only at a higher concentration. Furthermore, our data showed a pronounced G2/M cell cycle arrest following the 48 h treatment with **28**, with a simultaneous drastic reduction in the percentage of cells in the S phase. Precisely, the ability of compound **28** to enter the nucleus may be a reason for its pronounced effect on the cell cycle. Conversely, the influence of **36** on the cell cycle of HCT116 was significantly less pronounced, indicating that its pronounced cytotoxicity was mediated through a different mechanism. This finding was consistent with their different localizations within the cell.

Numerous studies have shown that harmine significantly influences the cell cycle of various cancer cell lines, including HCT116, via induction of G2/M cell cycle arrest, intercalation into DNA and induction of DNA fragmentation; upregulation of p53 and p21 expression; and downregulation of cyclin B1, p-cdc2, cdc2, cdc25, and p-cdc25 expression necessary for G2/M transition [47,48,49]. In our previous paper, we also confirmed that the parent compound harmine induced arrest in the G1 phase of MCF-7 cells already after 24 h, along with a reduction in cells in the S phase and an additional accumulation of cells in the G2/M phase after 48 h. The O-7-substituted TT harmine–coumarin derivative, which was synthesized by our research group, caused even more prominent G1 arrest than harmine, accompanied by a drastic reduction in the percentage of cells in the S phase, which also persisted after 48 h [34]. Harmicene **28**, also an O-7-substituted TT-harmine derivative, had an even more pronounced effect on the HCT116 cell cycle. Furthermore, it is known that ferrocene derivatives also inhibit the proliferation of various cancer cells through the induction of G0/G1 cell cycle arrest, downregulation of cyclin D1, CDK4 [50], and CDK6 and concomitant increases in p27 and p21 expression [51].

Considering all the facts mentioned above, harmicene **28** might significantly influence the cell cycle of the colorectal cancer cell line HCT116 as a result of its hybrid nature. However, we must stress the importance of a spacer type—a triazole ring—since the less selective compound **36**, differing only in the spacer type, did not show such an effect on the cell cycle. This finding indicates that their molecular targets are probably different and should be determined in future studies.

## 3. Materials and Methods

### 3.1. Chemistry

#### 3.1.1. General Information

Melting points were determined on a Stuart Melting Point Apparatus (Barloworld Scientific, Stone, UK) in open capillaries and were uncorrected. FTIR-ATR spectra were recorded using a Fourier-Transform Infrared Attenuated Total Reflection UATR Two spectrometer (PerkinElmer, Waltham, MA, USA) in the range of 450 to 4000 cm^−1^. ^1^H and ^13^C NMR spectra were recorded on a Bruker Avance III HD operating at 400 or 600 MHz for the ^1^H and 100, 101, or 151 MHz for the ^13^C nuclei (Bruker, Billerica, MA, USA). Samples were measured in DMSO-d_6_ solutions at 20 °C in 5 mm NMR tubes. Chemical shifts are reported in parts per million (ppm) using tetramethylsilane (TMS) as a reference in the ^1^H and the DMSO residual peak as a reference in the ^13^C spectra (39.52 ppm). Coupling constants (J) are reported in hertz (Hz). Mass spectra were recorded on an Agilent 1200 Series HPLC coupled with an Agilent 6410 Triple Quad (Agilent Technologies, Santa Clara, CA, USA). The mobile phase consisted of Milli-Q water as component A and MeOH (HPLC grade, J. T. Baker) as component B, and a Zorbax XDC C18 column (4.6 × 75 mm, 3.5 µm) was used as the stationary phase. Gradient elution was used at a flow rate of 0.5 mL/min, and 5 µL of analyte solution was injected per analysis. The starting conditions and gradient steepness were adjusted according to the analyte polarity. A diode array detector was utilized, and the data are presented as a total wavelength chromatogram (TWC). Mass spectrometry conditions were as follows: electrospray ionization (ESI) in positive and negative mode was used; the capillary voltage and current were set to 4.0 kV and 20 nA, respectively; the nebulizer pressure was set to 15 psi; and the drying gas (nitrogen) temperature and flow were 300 °C and 11 L/min, respectively. For the MS data analysis, Agilent MassHunter software (Agilent Technologies, Santa Clara, CA, USA) was used. Elemental analyses were performed on a CHNS LECO analyzer (LECO Corporation, St. Joseph, MI, USA). Analyses indicated by the symbols of the elements were within ±0.4% of their theoretical values. Microwave-assisted reactions were performed in a CEM Discover microwave reactor (CEM, Matthews, NC, USA) in a glass reaction vessel.

All compounds were routinely checked using TLC with silica gel 60F-254 glass plates (Merck KGaA, Darmstadt, Germany) using DCM/MeOH 8:1, 9:1, 97:3, cyclohexane/EtOAc/MeOH 1:1:0.5, 1:1:0.1, and acetone/DCM 1:1, 9:1, 3:2 as the solvent system. Spots were visualized using UV light (λ = 254 nm; 365 nm) and iodine vapor. Column chromatography was performed on silica gel 0.063–0.200 mm (Sigma-Aldrich, Waltham, MA, USA) with the same eluents used for TLC. All chemicals and solvents were of analytical grade and purchased from commercial sources.

ISA × HCl, alcohol **5**, and aldehydes **1** and **8** were prepared according to the procedures found in the literature [42,52,53]. Amines **4**, **7**, **11**, **14**, and **17**, azides **3** and **6**, and alkynes **10**, **13**, and **16** were prepared according to our published procedures [31,32,33].

#### 3.1.2. General Procedure for the Synthesis of Alkynes **2** and **9**

A corresponding aldehyde **1** or **8** (0.476 mmol) and K_2_CO_3_ (0.132 g, 0.952 mmol) were suspended in anhydrous MeOH (7.186 mL). Under a nitrogen atmosphere, Bestmann–Ohira reagent was added (0.857 mL, 5.712 mmol), and the reaction was stirred at room temperature for 3 h (alkyne **2**) or 48 h (alkyne **9**). Upon completion, MeOH was evaporated and 5% NaHCO_3_ was added dropwise until the precipitate formation was complete. The resulting precipitate was filtered off, purified using column chromatography with DCM/MeOH 9:1 (**2**) or cyclohexane/EtOAc/MeOH 1:1:0.1 (**9**) as a mobile phase, and triturated with a diethyl ether/petroleum ether mixture.

##### 1-Ethynyl-9*H*-pyrido[3,4-*b*]indole (**2**)

Aldehyde **1**: 0.093 g; yield: 0.068 g (74%); IR (ATR, ν/cm^−1^) 3289, 3154, 3061, 2111, 1626, 1562, 1498, 1452, 1424, 1387, 1321, 1273, 1246, 1151, 1081, 1066, 939, 874, 856, 837, 783, 748, 684, 631, 569, 513; ^1^H NMR (DMSO-d_6_) δ 11.79 (s, 1H), 8.34 (d, 1H, J = 5.2 Hz), 8.26 (d, 1H, J = 7.9 Hz), 8.17 (d, 1H, J = 5.2 Hz), 7.64 (d, 1H, J = 8.2 Hz), 7.60–7.56 (m, 1H), 7.32–7.24 (m, 1H), 4.77 (s, 1H); ^13^C NMR (DMSO-d_6_) δ 140.83, 138.66, 137.67, 128.67, 128.14, 125.42, 122.02, 120.69, 119.81, 115.32, 112.33, 84.94, 30.68; ESI-MS: *m*/*z* 193.1 (M + 1)^+^.

##### 3-Ethynyl-1-methyl-9*H*-pyrido[3,4-*b*]indole (**9**)

Aldehyde **8**: 0.100 g; yield: 0.060 g (61%); mp 230–235 °C; IR (ATR, ν/cm^−1^) 3306, 3143, 2104, 1622, 1597, 1563, 1499, 1446, 1375, 1338, 1315, 1281, 1247, 1178, 1147, 1014, 961, 899, 877, 776, 740, 654, 625, 587, 504, 457; ^1^H NMR (DMSO-d_6_) δ 11.83 (s, 1H), 8.25–8.22 (m, 1H), 8.21 (s, 1H), 7.62–7.59 (m, 1H), 7.57–7.53 (m, 1H), 7.27–7.23 (m, 1H), 4.03 (s, 1H), 2.74 (s, 3H); ^13^C NMR (DMSO-d_6_) δ 142.83, 140.70, 134.06, 129.54, 128.30, 127.01, 122.05, 120.72, 119.78, 117.22, 112.15, 84.85, 76.72, 20.33; ESI-MS: *m*/*z* 207.6 (M + 1)^+^.

#### 3.1.3. General Procedure for the Synthesis of Azides **12**, **15**, **18**

An appropriate amine **11**, **14**, or **17** (0.746 mmol); K_2_CO_3_ (0.258 g, 1.865 mmol); CuSO_4_ × 5H_2_O (q.s.); and ISA × HCl (0.188 g, 0.895 mmol) were suspended in anhydrous MeOH (2 mL). The mixture was stirred at room temperature for 2 h, the organic solvent was evaporated, and the residue was dissolved in H_2_O (15 mL) and extracted with EtOAc (2 × 40 mL). The collected organic layers were dried over anhydrous sodium sulfate, filtered, and evaporated under the reduced pressure. The crude product (oil) was purified using column chromatography (cyclohexane/EtOAc/MeOH 1:1:0.5).

##### 6-(2-Azidoethoxy)-1-methyl-9*H*-pyrido[3,4-*b*]indole (**12**)

Amine **11**: 0.180 g; yield: 0.185 g (93%).

##### 7-(2-Azidoethoxy)-1-methyl-9*H*-pyrido[3,4-*b*]indole (**15**)

Amine **14**: 0.180 g; yield: 0.158 g (79%); ^1^H NMR (DMSO-d_6_) δ 11.44 (s, 1H), 8.16 (d, 1H, J = 5.3 Hz), 8.08 (d, 1H, J = 8.6 Hz), 7.82 (d, 1H, J = 5.2 Hz), 7.04 (d, 1H, J = 2.2 Hz), 6.87 (dd, 1H, J = 8.6, 2.2 Hz), 4.30 (t, 2H, J = 4.8 Hz), 3.72 (t, 2H, J = 4.8 Hz), 2.73 (s, 3H); ^13^C NMR (DMSO-d_6_) δ 158.70, 141.77, 141.35, 137.78, 134.60, 127.12, 122.75, 115.26, 111.99, 109.14, 95.62, 67.14, 49.64, 20.33.

##### 9-(2-Azidoethyl)-7-methoxy-1-methyl-9*H*-pyrido[3,4-*b*]indole (**18**)

Amine **17**: 0.190 g; yield: 0.111 g (53%).

##### 3.1.4. (Azidomethyl)ferrocene (**19**)

To a suspension of ferrocenemethanol (0.100 g, 0.463 mmol) in dry THF (5 mL), ADMP (0.330 g, 1.158 mmol) and DBU (0.187 mL, 1.250 mmol) were added at 0 °C. The reaction was stirred at 0 °C for 1 h, diluted with saturated NH_4_Cl solution (40 mL), and extracted with DCM (2 × 30 mL). Organic layers were collected and washed with brine (2 × 30 mL) and H_2_O (1 × 20 mL), filtered, dried over anhydrous sodium sulfate, and the solvent evaporated under the reduced pressure. The crude product was purified using column chromatography (cyclohexane/EtOAc/MeOH 1:1:0.5); yield: 0.080 g (72%); oil; ^1^H NMR (DMSO-d_6_) δ 4.29 (t, 2H, J = 1.8 Hz), 4.21 (t, 2H, J = 1.9 Hz), 4.20 (s, 2H), 4.20 (s, 5H); ^13^C NMR (DMSO-d_6_) δ 81.78, 68.62, 68.58, 68.44, 50.02.

#### 3.1.5. General Procedure for the Synthesis of TT Harmicens **20**–**24**

An appropriate azide **3**, **6**, **12**, **15,** or **18** (0.209 mmol) and ethynylferrocene (0.040 g, 0.190 mmol) were dissolved in dry DMF (2 mL), followed by the addition of sodium ascorbate (0.202 mmol, 1 mL of freshly prepared 0.2 M solution in H_2_O) and CuSO_4_ × 5H_2_O (0.02 mmol, 20 µL of 1 M solution in H_2_O). The reaction mixture was stirred overnight at room temperature. After completion of the reaction, purification was performed using either Method A or Method B.

Method A: Ice-cold H_2_O (5 mL) was added to the reaction mixture and the resulting precipitate was filtered off, purified using column chromatography (with an additional Al_2_O_3_ layer to remove Cu salts, mobile phase cyclohexane/EtOAc/MeOH 1:1:0.5), and triturated with diethyl ether/petroleum ether mixture.

Method B: The reaction mixture was diluted with H_2_O (20 mL) and the product was extracted with EtOAc (2 × 30 mL). Organic layers were dried over anhydrous sodium sulfate, filtered, and the solvent evaporated under the reduced pressure. The crude product was purified using column chromatography (with an additional Al_2_O_3_ layer to remove Cu salts, mobile phase cyclohexane/EtOAc/MeOH) and triturated with a diethyl ether/petroleum ether mixture.

##### 1-((4-Ferrocenyl-1*H*-1,2,3-triazol-1-yl)methyl)-9*H*-pyrido[3,4-*b*]indole (**20**)

Azide **3**: 0.047 g; purification: Method A; yield: 0.064 g (78%); mp 252–255.5 °C; IR (ATR, ν/cm^−1^) 3249, 3138, 3094, 2987, 2953, 1625, 1585, 1567, 1497, 1455, 1430, 1389, 1321, 1236, 1212, 1189, 1094, 1058, 998, 875, 816, 794, 750, 730, 622, 592, 500; ^1^H NMR (DMSO-d_6_) δ 11.95 (s, 1H), 8.31 (d, 1H, J = 5.2 Hz), 8.28–8.26 (m, 2H), 8.13 (d, 1H, J = 5.1 Hz), 7.70–7.68 (m, 1H), 7.60 (ddd, 1H, J = 8.2, 7.1, 1.2 Hz), 7.28 (ddd, 1H, J = 8.0, 7.0, 1.0 Hz), 6.08 (s, 2H), 4.73 (t, 2H, J = 1.9 Hz), 4.28 (t, 2H, J = 1.8 Hz), 4.03 (s, 5H); ^13^C NMR (DMSO-d_6_) δ 145.22, 140.71, 138.17, 137.86, 133.85, 128.71, 128.54, 121.91, 121.57, 120.76, 119.64, 114.82, 112.09, 76.04, 69.23, 68.21, 66.34, 51.24; ESI-MS: *m*/*z* 434.9 (M + 1)^+^. HPLC purity > 99%. Anal. Calcd. for C_24_H_19_FeN_5_: C, 66.53; H, 4.42; N, 16.16; found: C, 66.87; H, 4.09; N, 16.54.

##### 1-Methyl-3-((4-ferrocenyl-1*H*-1,2,3-triazol-1-yl)methyl)-9*H*-pyrido[3,4-*b*]indole (**21**)

Azide **6**: 0.050 g; purification: Method B; cyclohexane/EtOAc/MeOH 1:1:0.1; yield: 0.053 g (62%); mp 242.5–244 °C; IR (ATR, ν/cm^−1^) 3148, 3101, 2993, 2891, 1737, 1627, 1566, 1506, 1456, 1443, 1351, 1328, 1251, 1219, 1086, 1057, 1019, 1000, 874, 815, 729, 680, 587, 506, 487; ^1^H NMR (DMSO-d_6_) δ 11.66 (s, 1H), 8.23 (s, 1H), 8.16 (d, 1H, J = 7.9 Hz), 7.90 (s, 1H), 7.60–7.59 (m, 1H), 7.55–7.52 (m, 1H), 7.24–7.21 (m, 1H), 5.76 (s, 2H), 4.74 (t, 2H, J = 1.9 Hz), 4.29 (t, 2H, J = 1.9 Hz), 4.03 (s, 5H), 2.76 (s, 3H); ^13^C NMR (DMSO-d_6_) δ 145.37, 142.95, 142.05, 140.77, 133.94, 128.08, 127.63, 121.64, 121.06, 120.91, 119.44, 112.09, 111.42, 76.09, 69.26, 68.21, 66.32, 55.10, 20.38; ESI-MS: *m*/*z* 448.9 (M + 1)^+^. Anal. Calcd. for C_25_H_21_FeN_5_: C, 67.13; H, 4.73; N, 15.66; found: C, 67.38; H, 4.54; N, 15.39.

##### 1-Methyl-6-(2-(4-ferrocenyl-1*H*-1,2,3-triazol-1-yl)ethoxy)-9*H*-pyrido[3,4-*b*]indole (**22**)

Azide **12**: 0.056 g; purification: Method B; mobile phase: cyclohexane/EtOAc/MeOH 1:1:0.5; yield: 0.034 g (37%); mp 201.5–204.0 °C; IR (ATR, ν/cm^−1^) 3133, 2949, 2872, 1583, 1568, 1497, 1458, 1286,1210, 1105, 1041, 988, 878, 818, 705, 621, 504; ^1^H NMR (DMSO-d_6_) δ 11.39 (s, 1H), 8.29 (s, 1H), 8.15 (d, 1H, J = 5.3 Hz), 7.87 (d, 1H, J = 5.3 Hz), 7.78 (d, 1H, J = 2.5 Hz), 7.48 (d, 1H, J = 8.8 Hz), 7.16 (dd, 1H, J = 8.8, 2.5 Hz), 4.82 (t, 2H, J = 5.1 Hz), 4.73 (t, 2H, J = 1.9 Hz), 4.53 (t, 2H, J = 5.2 Hz), 4.30 (t, 2H, J = 1.8 Hz), 4.01 (s, 5H), 2.72 (s, 3H); ^13^C NMR (DMSO-d_6_) δ 151.85, 145.24, 142.24, 136.98, 135.52, 135.10, 126.63, 121.35, 121.28, 118.14, 112.77, 112.64, 105.13, 76.05, 69.22, 68.21, 67.14, 66.34, 49.24, 20.40; ESI-MS: *m*/*z* 477.9 (M + 1)^+^. HPLC purity > 98%. Anal. Calcd. for C_26_H_23_FeN_5_O: C, 65.42; H, 4.86; N, 14.67; found: C, 65.69; H, 4.98; N, 14.37.

##### 1-Methyl-7-(2-(4-ferrocenyl-1*H*-1,2,3-triazol-1-yl)ethoxy)-9*H*-pyrido[3,4-*b*]indole (**23**)

Azide **15**: 0.056 g; purification: Method A; yield: 0.070 g, (77%); mp 228–229.5 °C; IR (ATR, ν/cm^−1^) 3125, 3077, 2959, 2850, 2772, 1623, 1566, 1443, 1426, 1301, 1278, 1240, 1185, 1108, 1053, 1042, 977, 874, 822, 810, 743, 720, 693, 638, 587, 570, 522, 513, 495, 483; ^1^H NMR (DMSO-d_6_) δ 11.44 (s, 1H), 8.30 (s, 1H), 8.14 (d, 1H, J = 5.3 Hz), 8.05 (d, 1H, J = 8.6 Hz), 7.79 (d, 1H, J = 5.3 Hz), 7.03 (d, 1H, J = 2.2 Hz), 6.85 (dd, 1H, J = 8.6, 2.2 Hz), 4.83 (t, 2H, J = 5.1 Hz), 4.73 (t, 2H, J = 1.9 Hz), 4.55 (t, 2H, J = 5.1 Hz), 4.30 (t, 2H, J = 1.8 Hz), 4.01 (s, 5H), 2.72 (s, 3H); ^13^C NMR (DMSO-d_6_) δ 158.62, 145.25, 141.72, 141.35, 137.76, 134.59, 127.07, 122.71, 121.34, 115.33, 111.98, 109.17, 95.73, 76.01, 69.23, 68.22, 66.57, 66.35, 49.09, 20.36; ESI-MS: *m*/*z* 477.9 (M + 1)^+^. HPLC purity > 99.5%. Anal. Calcd. for C_26_H_23_FeN_5_O: C, 65.42; H, 4.86; N, 14.67; found: C, 65.77; H, 4.56; N, 14.44.

##### 7-Methoxy-1-methyl-9-(2-(4-ferrocenyl-1*H*-1,2,3-triazol-1-yl)ethyl)-9*H*-pyrido[3,4-*b*]indole (**24**)

Azide **18**: 0.059 g; purification: Method A; yield: 0.059 g, (63%); mp 218–220 °C; IR (ATR, ν/cm^−1^) 2968, 1621, 1565, 1445, 1404, 1339, 1253, 1222, 1158, 1182, 1136, 1096, 1041, 1021, 970, 929, 877, 821, 807, 641, 590, 545, 501, 475; ^1^H NMR (DMSO-d_6_) δ 8.17 (d, 1H, J = 5.2 Hz), 8.04 (d, 1H, J = 8.5 Hz), 7.88 (d, 1H, J = 5.2 Hz), 7.80 (s, 1H), 6.84–6.78 (m, 2H), 5.08 (t, 2H, J = 5.6 Hz), 4.88 (t, 2H, J = 5.6 Hz), 4.49 (t, 2H, J = 1.9 Hz), 4.22 (t, 2H, J = 1.9 Hz), 3.86–3.85 (m, 8H), 2.90 (s, 3H); ^13^C NMR (DMSO-d_6_) δ 160.51, 145.15, 142.76, 140.67, 138.12, 134.68, 128.73, 122.23, 121.79, 114.10, 112.26, 109.81, 92.93, 75.78, 68.09, 68.06, 66.26, 55.37, 49.71, 44.47, 23.18; ESI-MS: *m*/*z* 491.9 (M + 1)^+^. HPLC purity > 99.5%. Anal. Calcd. for C_27_H_25_FeN_5_O: C, 66.00; H, 5.13; N, 14.25; found: C, 65.94; H, 4.89; N, 14.38.

#### 3.1.6. General Procedure for the Synthesis of TT Harmicens **25**–**29**

An appropriate alkyne **2**, **9**, **20**, **13**, or **16** (0.169 mmol) and (azidomethyl)ferrocene (**19**) (0.045 g, 0.186 mmol) were dissolved in dry DMF (2 mL), followed by the addition of sodium ascorbate (0.202 mmol, 1 mL of freshly prepared 0.2 M solution in H_2_O) and CuSO_4_ × 5 H_2_O (0.02 mmol, 20 µL of 1 M solution in H_2_O). The reaction mixture was stirred overnight at room temperature. After completion of the reaction, 5 mL of H_2_O was added and the precipitate was filtered off. Purification was performed using column chromatography (with an additional Al_2_O_3_ layer to remove Cu salts, mobile phase cyclohexane/EtOAc/MeOH 1:1:0.5 or DCM/MeOH 97:3) and triturated with a diethyl ether/petroleum ether mixture.

##### 1-(1-(Ferrocenylmethyl)-1*H*-1,2,3-triazol-4-yl)-9*H*-pyrido[3,4-*b*]indole (**25**)

Alkyne **2**: 0.032 g; yield: 0.037 g, (51%); mp 229–230 °C; IR (ATR, ν/cm^−1^) 3385, 3172, 3105, 3065, 2991, 1625, 1576, 1489, 1452, 1437, 1419, 1351, 1314, 1282, 1254, 1239, 1152, 1103, 1000, 829, 807, 749, 629, 591, 548, 499, 479; ^1^H NMR (DMSO-d_6_) δ 11.53 (s, 1H), 8.78 (s, 1H), 8.38 (d, 1H, J = 5.2 Hz), 8.26–8.24 (m, 1H), 8.11 (d, 1H, J = 5.1 Hz), 7.91–7.90 (m, 1H), 7.5–7.54 (m, 1H), 7.27–7.25 (m, 1H), 5.49 (s, 2H), 4.46 (t, 2H, J = 1.9 Hz), 4.24 (s, 5H), 4.22 (t, 2H, J = 1.9 Hz); ^13^C NMR (DMSO-d_6_) δ 147.60, 141.15, 137.83, 133.68, 131.74, 129.05, 128.18, 122.94, 121.47, 120.40, 119.51, 114.11, 113.23, 82.36, 68.74, 68.69, 68.48, 49.33; ESI-MS: *m*/*z* 434.1 (M + 1)^+^. HPLC purity > 99.5%. Anal. Calcd. for C_24_H_19_FeN_5_: C, 66.53; H, 4.42; N, 16.16; found: C, 66.38; H, 4.27; N, 16.32.

##### 3-(1-(Ferrocenylmethyl)-1*H*-1,2,3-triazol-4-yl)-1-methyl-9*H*-pyrido[3,4-*b*]indole (**26**)

Alkyne **9**: 0.035 g; yield: 0.036 g, (45%); mp 183–186 °C; IR (ATR, ν/cm^−1^) 3361, 2920, 2851, 1734, 1626, 1574, 1496, 1455, 1431, 1373, 1338, 1303, 1277, 1234, 1172, 1104, 1049, 1024, 1000, 924, 889, 818, 788, 775, 704, 631, 558, 584, 503, 478; ^1^H NMR (DMSO-d_6_) δ 11.69 (s, 1H), 8.58 (s, 1H), 8.41 (s, 1H), 8.29 (d, 1H, J = 7.9 Hz), 7.60 (d, 1H, J = 8.1 Hz), 7.54 (t, 1H, J = 7.6 Hz), 7.24 (t, 1H, J = 7.4 Hz), 5.39 (s, 2H), 4.45 (t, 2H, J = 1.9 Hz), 4.23 (s, 5H), 4.21 (t, 2H, J = 1.9 Hz), 2.80 (s, 3H); ^13^C NMR (DMSO-d_6_) δ 148.61, 141.93, 140.82, 139.95, 133.96, 128.02, 127.73, 121.97, 121.27, 121.25, 119.36, 112.01, 108.31, 82.45, 68.86, 68.68, 68.44, 49.05, 20.48; ESI-MS: *m*/*z* 447.9 (M + 1)^+^. HPLC purity > 97.5%. Anal. Calcd. for C_25_H_21_FeN_5_: C, 67.13; H, 4.73; N, 15.66; found: C, 67.41; H, 4.91; N, 15.36.

##### 6-((1-(Ferrocenylmethyl)-1*H*-1,2,3-triazol-4-yl)methoxy)-1-methyl-9*H*-pyrido[3,4-*b*]indole (**27**)

Alkyne **10**: 0.040 g; yield: 0.055 g, (68%); mp 245–248 °C; IR (ATR, ν/cm^−1^) 3631, 3137, 3081, 2950, 1637, 1603, 1583, 1567, 1499, 1480, 1451, 1411, 1283, 1211, 1107, 1069, 1054, 1040, 1027, 849, 821, 765, 620, 504; ^1^H NMR (DMSO-d_6_) δ 11.37 (s, 1H), 8.24 (s, 1H), 8.16 (d, 1H, J = 5.3 Hz), 7.89–7.88 (m, 2H), 7.49 (d, 1H, J = 8.8 Hz), 7.21 (dd, 1H, J = 8.9, 2.5 Hz), 5.32 (s, 2H), 5.21 (s, 2H), 4.32 (t, 2H, J = 1.8 Hz), 4.16–4.14 (m, 7H), 2.73 (s, 3H); ^13^C NMR (DMSO-d_6_) δ 151.88, 142.95, 142.19, 136.96, 135.42, 135.08, 126.69, 124.09, 121.34, 118.39, 112.73, 112.67, 105.20, 82.49, 68.62, 68.31, 61.88, 48.92, 20.40; ESI-MS: *m*/*z* 478.1 (M + 1)^+^. HPLC purity > 97%. Anal. Calcd. for C_26_H_23_FeN_5_O: C, 65.42; H, 4.86; N, 14.67; found: C, 65.25; H, 4.63; N, 14.42.

##### 7-((1-(Ferrocenylmethyl)-1*H*-1,2,3-triazol-4-yl)methoxy)-1-methyl-9*H*-pyrido[3,4-*b*]indole (**28**)

Alkyne **13**: 0.040 g; yield: 0.048 g, (60%); mp 242–244 °C; IR (ATR, ν/cm^−1^) 3344, 3072, 2869, 2785, 1738, 1626, 1567, 1488, 1444, 1377, 1325, 1305, 1290, 1278, 1177, 1142, 1110, 1058, 1142, 1011, 965, 828, 813, 781, 661, 639, 598, 572, 505, 480; ^1^H NMR (DMSO-d_6_) δ 11.45 (s, 1H), 8.23 (s, 1H), 8.15 (d, 1H, J = 5.2 Hz), 8.05 (d, 1H, J = 8.6 Hz), 7.80 (d, 1H, J = 5.3 Hz), 7.16 (d, 1H, J = 2.2 Hz), 6.89 (dd, 1H, J = 8.7, 2.3 Hz), 5.33 (s, 2H), 5.25 (s, 2H), 4.34 (t, 2H, J = 1.9 Hz), 4.18–4.16 (m, 7H), 2.72 (s, 3H); ^13^C NMR (DMSO-d_6_) δ 158.74, 142.71, 141.76, 141.33, 137.74, 134.58, 127.13, 124.15, 122.63, 115.11, 111.95, 109.46, 95.86, 82.41, 68.66, 68.64, 68.36, 61.45, 48.98, 20.32; ESI-MS: *m*/*z* 478.1 (M + 1)^+^. HPLC purity > 97%. Anal. Calcd. for C_26_H_23_FeN_5_O: C, 65.42; H, 4.86; N, 14.67; found: C, 65.71; H, 4.59; N, 14.82.

##### 9-((1-(Ferocenylmethyl)-1*H*-1,2,3-triazol-4-yl)methyl)-7-methoxy-1-methyl-9*H*-pyrido[3,4-*b*]indole (**29**)

Alkyne **16**: 0.042 g; yield: 0.025 g, (30%); mp 187–190 °C; IR (ATR, ν/cm^−1^) 3135, 3090, 2929, 1709, 1623, 1564, 1499, 1449, 1408, 1325, 1252, 1227, 1193, 1174, 1106, 1042, 1000, 913, 815, 764, 732, 638, 596, 550, 481;^1^H NMR (DMSO-d_6_) δ 8.16 (d, 1H, J = 5.2 Hz), 8.07 (d, 1H, J = 8.5 Hz), 7.98 (s, 1H), 7.86 (d, 1H, J = 5.2 Hz), 7.32 (d, 1H, J = 2.2 Hz), 6.87 (dd, 1H, J = 8.5, 2.2 Hz), 5.86 (s, 2H), 5.21 (s, 2H), 4.23 (t, 2H, J = 1.8 Hz), 4.12 (t, 2H, J = 1.8 Hz), 4.06 (s, 5H), 3.88 (s, 3H), 3.03 (s, 3H); ^13^C NMR (DMSO-d_6_) δ 160.55, 143.81, 142.67, 141.13, 138.05, 134.66, 128.54, 122.55, 122.38, 114.46, 112.26, 109.46, 94.05, 82.59, 68.54, 68.44, 68.21, 55.62, 48.77, 23.24; ESI-MS: *m*/*z* 492.1 (M + 1)^+^. HPLC purity > 99.5%. Anal. Calcd. for C_27_H_25_FeN_5_O C, 66.00; H, 5.13; N, 14.25; found: C, 66.23; H, 5.32; N, 14.59.

#### 3.1.7. General Procedure for the Synthesis of AT Harmicens **30**–**33**

A solution of a ferrocenecarboxylic acid (0.050 g, 0.217 mmol), DIEA (0.075 mL, 0.434 mmol), and HATU (0.083 g, 0.217 mmol) in DCM (4 mL) was stirred at room temperature for 20 min, followed by the addition of the corresponding amine **4**, **7**, **11**, or **17** (0.197 mmol). The resulting solution was stirred overnight at room temperature. Purification was performed using either Method A or Method B.

Method A: After completion of the reaction, the solvent was evaporated, and the residue was dissolved in EtOAc (20 mL) and washed with brine (2 × 20 mL) and water (1 × 20 mL). The organic layer was dried over anhydrous sodium sulfate, filtered, and evaporated under the reduced pressure. The crude product was purified using column chromatography with acetone/DCM as a mobile phase and triturated with diethyl ether/petroleum ether mixture.

Method B: The resulting precipitate was filtered off, purified using column chromatography (acetone/DCM 9:1), and triturated with diethyl ether/petroleum ether mixture.

##### *N*-((9*H*-Pyrido[3,4-*b*]indol-1-yl)methyl)ferrocenecarboxamide (**30**)

Amine **4**: 0.039 g; purification: Method A; mobile phase: acetone/DCM 1:1; yield: 0.039 g, (48%); mp 210–213 °C; IR (ATR, ν/cm^−1^) 3623, 3467, 3085, 3050, 2962, 2928, 1711, 1649, 1622, 1531, 1503, 1444, 1408, 1375, 1340, 1300, 1249, 1229, 1195, 138, 1123, 1106, 1041, 1017, 914, 819, 801, 634, 598, 558; ^1^H NMR (DMSO-d_6_) δ 11.49 (s, 1H), 8.58 (t, 1H, J = 6.0 Hz), 8.31 (d, 1H, J = 5.2 Hz), 8.23 (d, 1H, J = 7.8 Hz), 8.05 (d, 1H, J = 5.2 Hz), 7.68 (d, 1H, J = 8.2 Hz), 7.56 (t, 1H, J = 7.6 Hz), 7.25 (t, 1H, J = 7.5 Hz), 4.88–4.87 (m, 4H), 4.35 (t, 2H, J = 1.9 Hz), 4.07 (s, 5H); ^13^C NMR (DMSO-d_6_) δ 169.83, 142.64, 140.17, 137.35, 133.55, 128.10, 127.74, 121.74, 120.93, 119.37, 113.86, 111.99, 76.11, 70.10, 69.31, 68.30, 41.73; ESI-MS: *m*/*z* 410.0 (M + 1)^+^. Anal. Calcd. for C_23_H_19_FeN_3_O: C, 67.50; H, 4.68; N, 10.27; found: C, 67.78; H, 4.59; N, 10.62.

##### *N*-((1-Methyl-9*H*-pyrido[3,4-*b*]indol-3-yl)methyl)ferrocenecarboxamide (**31**)

Amine **7**: 0.042 g; purification: Method A; mobile phase: acetone/DCM 1:1; yield: 0.033 g, (39%); mp 240.5–243 °C; IR (ATR, ν/cm^−1^) 3324, 3226, 1634, 1574, 1532, 1498, 1447, 1380, 1348, 1297, 1248, 1104, 1026, 901, 814, 755, 738, 629, 528, 482; ^1^H NMR (DMSO-d_6_) δ 11.51 (s, 1H), 8.47 (t, 1H, J = 6.1 Hz), 8.12 (d, 1H, J = 7.9 Hz), 7.89 (s, 1H), 7.57 (d, 1H, J = 8.2 Hz), 7.52–7.48 (m, 1H), 7.21–7.17 (m, 1H), 4.90 (t, 2H, J = 1.9 Hz), 4.60 (d, 2H, J = 6.0 Hz), 4.37 (t, 2H, J = 1.9 Hz), 4.20 (s, 5H), 2.78 (s, 3H); ^13^C NMR (DMSO-d_6_) δ 168.94, 147.17, 141.14, 140.76, 133.49, 127.78, 127.66, 121.35, 120.95, 119.16, 111.98, 109.47, 76.73, 69.96, 69.25, 68.28, 44.31, 20.27; ESI-MS: *m*/*z* 424.0 (M + 1)^+^. Anal. Calcd. for C_24_H_21_FeN_3_O: C, 68.10; H, 5.00; N, 9.93; found: C, 68.28; H, 4.69; N, 9.62.

##### *N*-(2-((1-Methyl-9*H*-pyrido[3,4-*b*]indol-7-yl)oxy)ethyl)ferrocenecarboxamide (**32**)

Amine **11**: 0.048 g; purification: Method B; yield: 0.038 g, (42%); mp 243–245.5 °C; IR (ATR, ν/cm^−1^) 3216, 1716, 1622, 1551, 1451, 1422, 1376, 1312, 1278, 1236, 1217, 1105, 960, 842, 801, 741, 641, 605, 505,476; ^1^H NMR (DMSO-d_6_) δ 11.41 (s, 1H), 8.14 (d, 1H, J = 5.3 Hz), 8.07–8.05 (m, 2), 7.79 (d, 1H, J = 5.3 Hz), 7.07 (d, 1H, J = 2.4 Hz), 6.90 (dd, 1H, J = 8.7, 2.2 Hz), 4.83 (t, 2H, J = 2.0 Hz), 4.35 (t, 2H, J = 1.9 Hz), 4.23 (t, 2H, J = 5.7 Hz), 4.13 (s, 5H), 3.63 (q, 2H, J = 5.7 Hz), 2.71 (s, 3H); ^13^C NMR (DMSO-d_6_) δ 169.36, 159.31, 141.89, 141.28, 137.74, 134.55, 127.17, 122.69, 114.98, 111.93, 109.22 95.38, 76.31, 70.01, 69.40, 68.22, 66.40, 38.49, 20.33; ESI-MS: *m*/*z* 454.0 (M + 1)^+^. Anal. Calcd. for C_25_H_23_FeN_3_O_2_: C, 66.24; H, 5.11; N, 9.27; found: C, 65.99; H, 5.21; N, 9.54.

##### *N*-(2-(7-Methoxy-1-methyl-9*H*-pyrido[3,4-*b*]indol-9-yl)ethyl)ferrocenecarboxamide (**33**)

Amine **17**: 0.050 g; purification: Method A; mobile phase: acetone/DCM 3:2; yield: 0.046 g, (50%); mp 225–227 °C; IR (ATR, ν/cm^−1^) 3623, 3467, 3085, 3050, 2962, 2928, 1711, 1649, 1622, 1531, 1503, 1444, 1408, 1375, 1340, 1300, 1249, 1229, 1195, 1177, 1138, 1123, 1107, 1041, 1016, 914, 819, 801, 634, 598, 558; ^1^H NMR (DMSO-d_6_) δ 8.17 (d, 1H, J = 5.1 Hz), 8.10–8.07 (m, 2H), 7.88 (d, 1H, J = 5.1 Hz), 7.32 (d, 1H, J = 2.2 Hz), 6.88 (dd, 1H, J = 8.5, 2.1 Hz), 4.69 (t, 2H, J = 1.9 Hz), 4.66 (t, 2H, J = 7.1 Hz), 4.34 (t, 2H, J = 1.9 Hz), 4.07 (s, 5H), 3.91 (s, 3H), 3.60 (q, 2H, J = 6.7 Hz), 3.04 (s, 3H); ^13^C NMR (DMSO-d_6_) δ 169.75, 160.52, 143.00, 140.66, 137.82, 134.67, 128.43, 122.39, 114.28, 112.26, 109.20, 93.78, 76.40, 69.93, 69.32, 68.08, 55.50, 43.56, 39.1, 23.14; ESI-MS: *m*/*z* 468.1 (M + 1)^+^. Anal. Calcd. for C_26_H_25_FeN_3_O_2_: C, 66.82; H, 5.39; N, 8.99; found: C, 66.78; H, 5.52; N, 9.15.

#### 3.1.8. General Procedure for the Synthesis of AT Harmicens **34**–**37**

A solution of a ferroceneacetic acid (0.050 g, 0.205 mmol), DIEA (0.071 mL, 0.410 mmol), and HATU (0.078 g, 0.205 mmol) in DCM (4 mL) was stirred at room temperature for 20 min, followed by the addition of the corresponding amine **4**, **7**, **11**, or **17** (0.186 mmol). The resulting solution was stirred at room temperature for 1 h and purified using either Method A or Method B.

Method A: The resulting precipitate was filtered off and recrystallized from EtOH.

Method B: The reaction mixture was extracted with brine (2 × 20 mL) and water (1 × 20 mL). The organic layer was dried over anhydrous sodium sulfate, filtered, and evaporated under the reduced pressure. The crude product was triturated with a diethyl ether/petroleum ether mixture.

##### *N*-((9*H*-Pyrido[3,4-*b*]indol-1-yl)methyl)-2-ferrocenylacetamide (**34**)

Amine **4**: 0.037 g; purification: Method A; yield: 0.027 g, (34%); mp 222.0–224.5 °C; IR (ATR, ν/cm^−1^) 3333, 3219, 3168, 3099, 2995, 2898, 1651, 1568, 1521, 1502, 1445, 1436, 1413, 1361, 1321, 1285, 1248, 1151, 1106, 1045, 1026, 999, 878, 820, 740, 677, 595, 564, 504; ^1^H NMR (DMSO-d_6_) δ 11.50 (s, 1H), 8.55 (t, 1H, J = 5.4 Hz), 8.29 (d, 1H, J = 5.2 Hz), 8.22 (d, 1H, J = 7.8 Hz), 8.04 (d, 1H, J = 5.2 Hz), 7.63 (d, 1H, J = 8.2 Hz), 7.55 (t, 1H, J = 7.6 Hz), 7.25 (t, 1H, J = 7.4 Hz), 4.76 (d, 2H, J = 5.4 Hz), 4.22 (t, 2H, J = 1.9 Hz), 4.05 (m, 7H), 3.26 (s, 2H); ^13^C NMR (DMSO-d_6_) δ 170.47, 141.60, 140.36, 137.36, 133.50, 128.12, 127.74, 121.73, 120.87, 119.39, 113.89, 112.05, 82.66, 68.58, 68.41, 67.11, 41.52, 36.33; ESI-MS: *m*/*z* 424.1 (M + 1)^+^; HPLC purity > 99.5%. Anal. Calcd. for C_24_H_21_FeN_3_O: C, 68.10; H, 5.00; N, 9.93; found: C, 68.48; H, 4.79; N, 9.63.

##### *N*-((1-Methyl-9*H*-pyrido[3,4-*b*]indol-3-yl)methyl)-2-ferrocenylacetamide (**35**)

Amine **7**: 0.039 g; purification: Method A; yield: 0.032 g, (39%); mp 247.5–250.0 °C; IR (ATR, ν/cm^−1^) 3404, 3191, 3081, 2944, 1649, 1621, 1570, 1522, 1471, 1455, 1421, 1312, 1247, 1267, 1136, 1103, 1038, 1023, 997, 925, 826, 803, 753, 741, 693, 621, 584, 543, 500, 482; ^1^H NMR (DMSO-d_6_) δ 11.48 (s, 1H), 8.42 (t, 1H, J = 5.9 Hz), 8.10 (d, 1H, J = 7.8 Hz), 7.73 (s, 1H), 7.56 (d, 1H, J = 8.2 Hz), 7.52–7.50 (m, 1H), 7.21 (t, 1H, J = 7.4 Hz), 4.46 (d, 2H, J = 5.8 Hz), 4.27 (t, 2H, J = 1.9 Hz), 4.12 (s, 5H), 4.10 (t, 2H, J = 1.9 Hz), 3.24 (s, 2H), 2.73 (s, 3H); ^13^C NMR (DMSO-d_6_) δ 169.93, 146.33, 141.16, 140.71, 133.44, 127.78, 127.66, 121.49, 120.98, 119.11, 111.94, 109.54, 83.05, 68.63, 68.45, 67.15, 44.28, 36.72, 20.28; ESI-MS: *m*/*z* 438.0 (M + 1)^+^; HPLC purity > 99.5%. Anal. Calcd. for C_25_H_23_FeN_3_O: C, 68.66; H, 5.30; N, 9.61; found: C, 68.75; H, 5.47; N, 9.32.

##### *N*-(2-((1-Methyl-9*H*-pyrido[3,4-*b*]indol-7-yl)oxy)ethyl)-2-ferrocenylacetamide (**36**)

Amine **11**: 0.045 g; purification: Method B; yield: 0.050 g, (58%); mp 176.0–177.5 °C; IR (ATR, ν/cm^−1^) 3222, 3055, 2875, 1656, 1630, 1539, 1484, 1434, 1378, 1324, 1299, 1273, 1238, 1174, 1134, 1106, 1073, 1037, 1023, 1002, 961, 924, 871, 814, 776, 738, 661, 634, 590, 567, 484; ^1^H NMR (DMSO-d_6_) δ 11.40 (s, 1H), 8.19 (t, 1H, J = 5.5 Hz), 8.14 (d, 1H, J = 5.3 Hz), 8.05 (d, 1H, J = 8.6 Hz), 7.80 (d, 1H, J = 5.3 Hz), 7.02 (d, 1H, J = 2.2 Hz), 6.85 (dd, 1H, J = 8.6, 2.2 Hz), 4.20 (t, 2H, J = 1.9 Hz), 4.13 (t, 2H, J = 5.5 Hz), 4.11 (s, 5H), 4.05 (t, 2H, J = 1.9 Hz), 3.51 (q, 2H, J = 5.6 Hz), 3.16 (s, 2H), 2.72 (s, 3H); ^13^C NMR (DMSO-d_6_) δ 170.25, 159.17, 141.84, 141.29, 137.75, 134.56, 127.16, 122.63, 115.00, 111.93, 109.30, 95.40, 82.72, 68.50, 68.43, 67.08, 66.55, 38.40, 36.37, 20.35; ESI-MS: *m*/*z* 468.1 (M + 1)^+^; HPLC purity > 99.5%. Anal. Calcd. For C_26_H_25_FeN_3_O_2_: C, 66.82; H, 5.39; N, 8.99; found: C, 66.53; H, 5.67; N, 9.12.

##### *N*-(2-(7-Methoxy-1-methyl-9*H*-pyrido[3,4-*b*]indol-9-yl)ethyl)-2-ferrocenylacetamide (**37**)

Amine **17**: 0.047 g; purification: Method B; yield: 0.062 g, (69%); mp 170.5–172.0 °C; IR (ATR, ν/cm^−1^) 3341, 2931, 1655, 1624, 1566, 1503, 1451, 1439, 1410, 1347, 1330, 1300, 1251, 1195, 1172, 1141, 1104, 1048, 1024, 929, 835, 813, 794, 639, 567, 496, 482; ^1^H NMR (DMSO-d_6_) δ 8.17 (d, 1H, J = 5.1 Hz), 8.09 (d, 1H, J = 8.6 Hz), 8.01 (t, 1H, J = 6.0 Hz), 7.88 (d, 1H, J = 5.1 Hz), 7.27 (d, 1H, J = 2.2 Hz), 6.89 (dd, 1H, J = 8.6, 2.2 Hz), 4.57 (t, 2H, J = 7.1 Hz), 4.13 (t, 2H, J = 1.9 Hz), 4.11 (s, 5H), 4.05 (t, 2H, J = 1.8 Hz), 3.92 (s, 3H), 3.45 (q, 2H, J = 6.7 Hz), 3.07 (s, 2H), 2.95 (s, 3H); ^13^C NMR (DMSO-d_6_) δ 170.58, 160.52, 142.86, 140.61, 137.80, 134.64, 128.43, 122.40, 114.31, 112.25, 109.24, 93.64, 82.12, 68.72, 68.46, 67.21, 55.52, 43.37, 38.73, 36.65, 23.08; ESI-MS: *m*/*z* 482.2 (M + 1)^+^. Anal. Calcd. For C_27_H_27_FeN_3_O_2_: C, 67.37; H, 5.65; N, 8.73; found: C, 67.16; H, 5.84; N, 8.89.

### 3.2. Biological Evaluation

#### 3.2.1. In Vitro Drug Sensitivity Assay against Erythrocytic Stages of *P. falciparum*

The antiplasmodial activity of harmicens **20**–**37** was evaluated against two strains of *P. falciparum* (3D7—CQ-sensitive, Dd2—multidrug-resistant) as previously described using the histidine-rich protein 2 (HRP2) assay [44,45]. Briefly, 96-well plates were pre-coated with the tested compounds in a three-fold dilution before ring-stage parasites were added to complete the culture medium at a hematocrit of 1.5% and parasitemia of 0.05%. After three days of incubation at 37 °C, 5% CO_2_, and 5% oxygen, plates were frozen until HRP2-ELISA analysis. All compounds were evaluated in duplicate in at least two independent experiments. The IC_50_ was determined using a nonlinear regression analysis of log concentration–response curves using the drc-package v0.9.0 of R v2.6.1 (R Foundation, Vienna, Austria) [46].

#### 3.2.2. Cytotoxicity Assay in Human Cell Lines

The experiments were carried out on five human cell lines purchased from American Type Culture Collection (ATCC): HepG2 (hepatocellular carcinoma; ATCC^®^ HB-8065™), SW620 (colorectal adenocarcinoma; ATCC^®^ CCL-227™), HCT116 (colorectal carcinoma; ATCC^®^ CCL-247™), MCF-7 (breast adenocarcinoma; ATCC^®^ HTB-22™), and Hek293T (embryonic kidney cells; ATCC^®^ CRL-3216™). All cell lines were cultured as monolayers and maintained in Dulbecco’s Modified Eagle Medium (DMEM) (Capricorn Scientific, Ebsdorfergrund, Germany) supplemented with 10% fetal bovine serum (FBS) (Capricorn Scientific, Ebsdorfergrund, Germany), 100 U/mL penicillin, and 100 µg/mL streptomycin (Capricorn Scientific, Ebsdorfergrund, Germany) in a humidified atmosphere with 5% CO_2_ at 37 °C. Cells were seeded in 96-well plates (Corning, Durham, NC, USA) at 5000–7000 cells per well (depending on the cell-doubling time of a specific cell line) in 0.1 mL media and cultured for 24 h. The next day, the medium was aspirated, and cells were treated for 72 h. Only the compounds that led to more than a 50% reduction in mitochondrial metabolic activity at a concentration of 50 µM were selected for further analysis. The following concentrations of selected compounds were used: 25, 10, 5, and 1 µM. Working dilutions were freshly prepared on the day of the testing. A fresh growth medium was added to untreated control cells, which were defined as 100% viable. DMSO (0.13%) in DMEM was considered a negative control. 5-Fluorouracil (5-FU) and harmine were used as positive controls. At the end of treatment, the medium was removed, and cells were incubated for 1 h with 0.5 mg/mL MTT (Abcam, Cambridge, MA, USA) dissolved in serum-deprived DMEM. The MTT-containing medium was then removed, and 0.1 mL isopropanol was added per well to lyse cells and dissolve formazan. The optical density was measured at 570 nm using a microplate reader (VICTOR3, PerkinElmer, Waltham, MA, USA). Each test point was performed in triplicate. The absorbance was directly proportional to the cell viability. The IC_50_ values (concentration required to decrease viability by 50%) were calculated by using nonlinear regression on the sigmoidal dose–response plots and are expressed as mean ± SD.

#### 3.2.3. Cell Localization

The MCF-7 cells were seeded onto round microscopic glass coverslips placed in 24-well plates at a density of 5 × 10^4^ cells per well and grown at 37 °C and 5% CO_2_ for 24 h in DMEM supplemented with FBS, penicillin, and streptomycin, as described above. On the next day, the growth medium was replaced with compound **28** at 10 and 50 µM concentrations or compound **36** at a 5 µM concentration in a complete cell culture medium and incubated for 30 min and 1 h. Afterward, the medium was discarded and the coverslips were rinsed twice with PBS, placed on the microscopic slides, and immediately analyzed. The uptake and intracellular distribution of the tested derivative were analyzed under a fluorescence microscope (Olympus BX51, Tokyo, Japan) at 400× magnification using a DAPI filter. Images were captured with an Olympus DP70 digital camera.

#### 3.2.4. Cell Cycle Analysis

HCT116 cells were seeded onto 6-well plates (2 × 10^5^ cells per well). After 24 h, the tested compounds **28** and **36** were added at two concentrations—3 µM and 6 µM. After 24 h and 48 h, the attached cells were trypsinized, combined with floating cells, washed with phosphate buffer saline (PBS), fixed with 70% ethanol, and stored at −20 °C. Immediately before analysis, the cells were washed two times with PBS, treated with 0.1 μg/μL of RNAse A, and stained for 1 h on ice with 50 μg/mL of propidium iodide. The stained cells were then analyzed using a BD FACScalibur flow cytometer (20,000 counts were measured). The percentage of cells in each cell cycle phase was determined using FlowJo software (TreeStar Inc., San Francisco, CA, USA). The tests were performed in duplicate and repeated in two separate experiments. The average values from duplicates from one representative experiment ± SD are presented.

#### 3.2.5. Statistical Analysis

Data are presented as mean ± SD for the indicated number of independent experiments. Statistical significance (*p* < 0.05) was determined via one-way ANOVA using the Microsoft Excel Analysis ToolPak add-in (Microsoft 365 MSO, v.2206 build 16.0.15330.20216, Redmond, WA, USA).

## 4. Conclusions

We synthesized 18 harmicens—new hybrids combining harmine/β-carboline and ferrocene motifs. The compounds obtained differed in the type and length of the linker between the β-carboline ring and ferrocene, as well as its position at the β-carboline ring. Evaluation of their antiplasmodial activity in vitro against the erythrocytic stages of *P. falciparum* showed that the hybrids had moderate antiplasmodial activity in the submicromolar/low micromolar range. On the other hand, their antiproliferative activity in vitro was significant. Several compounds exhibited selective activity against tumor cells (MCF-7 and HCT116) compared to a nontumor cell line (Hek293T) (IC_50_ in the single-digit micromolar range, SI > 5.9). We further demonstrated that cell localization and its effect on the cell cycle were markedly different for HCT116-selective **28** and nonselective **36**. The experiments revealed that **28** penetrated the nucleus and induced G2/M cell cycle arrest with a concomitant drastic reduction in the percentage of cells in the S phase, whereas the effect of the nonselective compound **36** on the cell cycle was much less pronounced. We believe that our study clearly demonstrated the potential of harmicens as valuable anticancer hits. In future experiments, we will focus on the enrichment of the harmicene library to establish a reliable structure–activity relationship and the elucidation of their mechanism of action.

## Figures and Tables

**Table 1:** In vitro antiplasmodial activity of harmicens **20**–**37** against the erythrocytic stages of *P. falciparum* (*Pf*3D7 and *Pf*Dd2 strains).

Compd.	IC_50_ ^a^ (µM)				
	*Pf*3D7	*Pf*Dd2		*Pf*3D7	*Pf*Dd2
**20**	9.23 ± 1.96	11.20 ± 2.23	**30**	>56 ^b^	14.01 ± 1.95 ^c^
**21**	7.14 ± 0.38	6.06 ± 1.36	**31**	13.24 ± 8.54	8.03 ± 0.19
**22**	0.35 ± 0.19	0.89 ± 0.01	**32**	0.30 ± 0.09	0.66 ± 0.01
**23**	0.15 ± 0.05	1.02 ± 0.52	**33**	0.40 ± 0.18	2.04 ± 0.29
**24**	1.35 ± 0.28	2.31 ± 0.29	**34**	1.34 ± 0.09	1.11 ± 0.01
**25**	3.62 ± 1.08	4.37 ± 1.79	**35**	7.85 ± 2.18	12.99 ± 5.90
**26**	2.63 ± 0.27	4.02 ± 0.61	**36**	0.42 ± 0.02	1.53 ± 0.06
**27**	0.31 ± 0.01	1.09 ± 0.01	**37**	0.83 ± 0.23	1.80 ± 0.35
**28**	>56	>56	HAR ^d^	8.25 ± 2.83	>27.7
**29**	2.12 ± 0.81	2.91 ± 0.05	CQ ^e^	0.004 ± 0.002	0.29 ± 0.10

^a^ IC_50_, the concentration of the tested compound necessary for 50% growth inhibition. ^b^ The exact IC_50_ could not be obtained, as activity could only be detected at the highest tested concentration. ^c^ Results represent mean ± SD, n > 2. ^d^ HAR, harmine; ^e^ CQ, chloroquine.

**Table 2:** In vitro antiproliferative activity of harmicens **20**–**37** against human cell lines and calculated selectivity indices.

Compd.	IC_50_ ^a^ (µM)					SI ^b^ (HCT116)	SI (MCF-7)
	HepG2	SW620	HCT116	MCF-7	Hek293T		
**20**	43.9 ± 1.1	>50	29.4 ± 4.6	39.4 ± 7.9	>50	>1.7	>1.3
**21**	>50	19.7 ± 1.9	8.3 ± 1.4	17.7 ± 0.6	>50	>6	>2.8
**22**	8.4 ± 0.9	6.5 ± 0.7	9 ± 0.7	6.9 ± 0.5	7.3 ± 2.8	0.8	1.1
**23**	9.5 ± 0.5	7.6 ± 0.1	38.6 ± 2.8	8.0 ± 0.2	17.1 ± 1.8	0.4	2.1
**24**	>50	>50	>50	>50	>50	>1	>1
**25**	>50	23.1 ± 6.4	7.6 ± 0.5	>50	>50	>6.6	>1
**26**	12.3 ± 5	3.8 ± 0.4	35.2 ± 4.7	3.7 ± 0.2	46.5 ± 1	1.3	12.6
**27**	17 ± 0.3	8.1 ± 0.2	>50	6.8 ± 0.2	43 ± 8.6	< 0.9	6.3
**28**	>50	>50	7.4 ± 0.4	>50	>50	>6.8	>1
**29**	>50	>50	14.6 ± 3.7	>50	>50	>3.4	>1
**30**	>50	45.1 ± 3.7	16.8 ± 0.9	8.5 ± 1.5	>50	>3	>5.9
**31**	>50	>50	>50	19.9 ± 1.1	>50	>1	>2.51
**32**	15.6 ± 0.3	9.9 ± 4	6.6 ± 0.8	9.1 ± 1.4	18.6 ± 0.2	2.8	2
**33**	>50	>50	16.3 ± 3.2	30.5 ± 13.2	>50	>1	>1.7
**34**	>50	>50	>50	>50	>50	>1	>1
**35**	>50	25 ± 8.1	20.9 ± 1.5	36.9 ± 6.8	>50	>2.4	>1.4
**36**	7.4 ± 0.5	4.2 ± 0.3	5.9 ± 1.3	4.5 ± 0.3	8.4 ± 0.3	1.4	1.9
**37**	28 ± 2.5	9.9 ± 1.1	7.5 ± 0.4	13.2 ± 0.5	30.8 ± 2.4	4.1	2.3
HAR ^c^	18.7 ± 0.8	4.7 ± 0.6	4.0 ± 0.8	13.5 ± 1.1	12.6 ± 0.8	3.2	0.9
5-FU ^d^	5.5 ± 0.6	9.4 ± 0.3	5.2 ± 2.8	23.9 ± 5.7	8.1 ± 0.8	1.6	0.3

^a^ IC_50_, the concentration that causes 50% growth inhibition; ^b^ SI, selectivity index; ^c^ HAR, harmine; ^d^ 5-FU, 5-fluorouracil.

## Data Availability

The data presented in this study are available in this article and in the Supplementary Materials.

## Supplementary Materials

The supporting information can be downloaded at: https://www.mdpi.com/article/10.3390/ijms23169315/s1.

## References

[1] World Health Organization. https://www.who.int/news-room/fact-sheets/detail/cancer.

[2] Ferlay J., Colombet M., Soerjomataram I., Parkin D.M., Piñeros M., Znaor A., Freddie B. (2021). Cancer statistics for the year 2020: An overview. Int. J. Cancer..

[3] World Health Organization International Agency for Research on Cancer. https://gco.iarc.fr/today.

[4] Wang R., Chen H., Yan W., Zheng M., Zhang T., Zhang Y. (2020). Ferrocene-containing hybrids as potential anticancer agents: Current developments, mechanisms of action and structure-activity relationships. Eur. J. Med. Chem..

[5] Sato S. (2021). *Plasmodium*—A brief introduction to the parasites causing human malaria and their basic biology. J. Physiol. Anthropol..

[6] Yeung S. (2018). Malaria-Update on Antimalarial Resistance and Treatment Approaches. Pediatr. Infect. Dis. J..

[7] World Health Organization World Malaria Report 2021. https://www.who.int/publications/i/item/9789240040496.

[8] Nepali K., Sharma S., Sharma M., Bedi P.M., Dhar K.L. (2014). Rational approaches, design strategies, structure activity relationship and mechanistic insights for anticancer hybrids. Eur. J. Med. Chem..

[9] Soni J.P., Yeole Y., Shankaraiah N. (2021). β-Carboline-based molecular hybrids as anticancer agents: A brief sketch. RSC Med. Chem..

[10] Walsh J.J., Bell A. (2009). Hybrid drugs for malaria. Curr. Pharm. Des..

[11] Cao R., Peng W., Wang Z., Xu A. (2007). beta-Carboline alkaloids: Biochemical and pharmacological functions. Curr. Med. Chem..

[12] Ishida J., Wang H.K., Bastow K.F., Hu C.Q., Lee K.H. (1999). Antitumor agents 201. Cytotoxicity of harmine and β-carboline analogs. Bioorg. Med. Chem. Lett..

[13] Guan H., Chen H., Peng W., Ma Y., Cao R., Liu X., Xu A. (2006). Design of beta-carboline derivatives as DNA-targeting antitumor agents. Eur. J. Med. Chem..

[14] Xu Q.B., Chen X.F., Feng J., Miao J.F., Liu J., Liu F.T., Niu B.X., Cai J.Y., Huang C., Zhang Y. (2016). Design, synthesis and biological evaluation of hybrids of β-carboline and salicylic acid as potential anticancer and apoptosis inducing agents. Sci. Rep..

[15] Aaghaz S., Sharma K., Jain R., Kamal A. (2021). β-Carbolines as potential anticancer agents. Eur. J. Med. Chem..

[16] Meinguet C., Bruyère C., Frédérick R., Mathieu V., Vancraeynest C., Pochet L., Laloy J., Mortier J., Wolber G., Kiss R. (2015). 3D-QSAR, design, synthesis and characterization of trisubstituted harmine derivatives with *in vitro* antiproliferative properties. Eur. J. Med. Chem..

[17] Kumar S., Singh A., Kumar K., Kumar V. (2017). Recent insights into synthetic β-carbolines with anti-cancer activities. Eur. J. Med. Chem..

[18] Shahinas D., Macmullin G., Benedict C., Crandall I., Pillai D.R. (2012). Harmine is a potent antimalarial targeting Hsp90 and synergizes with chloroquine and artemisinin. Antimicrob. Agents Chemother..

[19] Gorki V., Walter N.S., Singh R., Chauhan M., Dhingra N., Salunke D.B., Kaur S. (2020). β-Carboline Derivatives Tackling Malaria: Biological Evaluation and Docking Analysis. ACS Omega.

[20] Kamboj A., Sihag B., Brar D.S., Kaur A., Salunke D.B. (2021). Structure activity relationship in β-carboline derived anti-malarial agents. Eur. J. Med. Chem..

[21] Heinze K., Lang H. (2013). Ferrocene—Beauty and Function. Organometallics.

[22] Sharma B., Kumar V. (2021). Has Ferrocene Really Delivered Its Role in Accentuating the Bioactivity of Organic Scaffolds?. J. Med. Chem..

[23] Roux C., Biot C. (2012). Ferrocene-based antimalarials. Future Med. Chem..

[24] Peter S., Aderibigbe B.A. (2019). Ferrocene-Based Compounds with Antimalaria/Anticancer Activity. Molecules.

[25] Ren S.Z., Wang Z.C., Zhu D., Zhu X.H., Shen F.Q., Wu S.Y., Chen J.J., Xu C., Zhu H.L. (2018). Design, synthesis and biological evaluation of novel ferrocene-pyrazole derivatives containing nitric oxide donors as COX-2 inhibitors for cancer therapy. Eur. J. Med. Chem..

[26] Chellan P., Sadler P.J. (2020). Enhancing the Activity of Drugs by Conjugation to Organometallic Fragments. Chem. Eur. J..

[27] Nguyen A., Top S., Pigeon P., Vessières A., Hillard E.A., Plamont M.A., Huché M., Rigamonti C., Jaouen G. (2009). Synthesis and structure-activity relationships of ferrocenyl tamoxifen derivatives with modified side chains. Chem. Eur. J..

[28] Dubar F., Khalife J., Brocard J., Dive D., Biot C. (2008). Ferroquine, an ingenious antimalarial drug: Thoughts on the mechanism of action. Molecules.

[29] Adoke Y., Zoleko-Manego R., Ouoba S., Tiono A.B., Kaguthi G., Bonzela J.E., Duong T.T., Nahum A., Bouyou-Akotet M., Ogutu B. (2021). A randomized, double-blind, phase 2b study to investigate the efficacy, safety, tolerability and pharmacokinetics of a single-dose regimen of ferroquine with artefenomel in adults and children with uncomplicated *Plasmodium falciparum* malaria. Malar. J..

[30] Kondratskyi A., Kondratska K., Vanden Abeele F., Gordienko D., Dubois C., Toillon R.A., Slomianny C., Lemière S., Delcourt P., Dewailly E. (2017). Ferroquine, the next generation antimalarial drug, has antitumor activity. Sci. Rep..

[31] Perković I., Raić-Malić S., Fontinha D., Prudêncio M., Pessanha de Carvalho L., Held J., Tandarić T., Vianello R., Zorc B., Rajić Z. (2020). Harmicines-harmine and cinnamic acid hybrids as novel antiplasmodial hits. Eur. J. Med. Chem..

[32] Marinović M., Perković I., Fontinha D., Prudêncio M., Held J., Pessanha de Carvalho L., Tandarić T., Vianello R., Zorc B., Rajić Z. (2020). Novel Harmicines with Improved Potency against *Plasmodium*. Molecules.

[33] Marinović M., Poje G., Perković I., Fontinha D., Prudêncio M., Held J., Pessanha de Carvalho L., Tandarić T., Vianello R., Rajić Z. (2021). Further investigation of harmicines as novel antiplasmodial agents: Synthesis, structure-activity relationship and insight into the mechanism of action. Eur. J. Med. Chem..

[34] Pavić K., Beus M., Poje G., Uzelac L., Kralj M., Rajić Z. (2021). Synthesis and Biological Evaluation of Harmirins, Novel Harmine-Coumarin Hybrids as Potential Anticancer Agents. Molecules.

[35] Poje G., Pessanha de Carvalho L., Held J., Moita D., Prudêncio M., Perković I., Tandarić T., Vianello R., Rajić Z. (2022). Design and synthesis of harmiquins, harmine and chloroquine hybrids as potent antiplasmodial agents. Eur. J. Med. Chem..

[36] Allardyce C.S., Dorcier A., Scolaro C., Dyson P.J. (2005). Development of organometallic (organo-transitionmetal) pharmaceuticals. Appl. Organometal. Chem..

[37] Hartinger C.G., Dyson P.J. (2009). Bioorganometallic chemistry--from teaching paradigms to medicinal applications. Chem. Soc. Rev..

[38] Matos J., da Cruz F.P., Cabrita É., Gut J., Nogueira F., do Rosário V.E., Moreira R., Rosenthal P.J., Prudêncio M., Gomes P. (2012). Novel potent metallocenes against liver stage malaria. Antimicrob. Agents Chemother..

[39] Patra M., Gasser G. (2017). The medicinal chemistry of ferrocene and its derivatives. Nat. Rev. Chem..

[40] Wani W.A., Jameel E., Baig U., Mumtazuddin S., Hun L.T. (2015). Ferroquine and its derivatives: New generation of antimalarial agents. Eur. J. Med. Chem..

[41] Ludwig B.S., Correia J.D.G., Kühn F.E. (2019). Ferrocene derivatives as anti-infective agents. Coord. Chem. Rev..

[42] Habrant D., Rauhala V., Koskinen A.M. (2010). Conversion of carbonyl compounds to alkynes: General overview and recent developments. Chem. Soc. Rev..

[43] Goddard-Borger E.D., Stick R.V. (2007). An efficient, inexpensive, and shelf-stable diazotransfer reagent: Imidazole-1-sulfonyl azide hydrochloride. Org. Lett..

[44] Held J., Gebru T., Kalesse M., Jansen R., Gerth K., Müller R., Mordmüller B. (2014). Antimalarial activity of the myxobacterial macrolide chlorotonil a. Antimicrob. Agents Chemother..

[45] Noedl H., Bronnert J., Yingyuen K., Attlmayr B., Kollaritsch H., Fukuda M. (2005). Simple histidine-rich protein 2 double-site sandwich enzyme-linked immunosorbent assay for use in malaria drug sensitivity testing. Antimicrob. Agents Chemother..

[46] The R Project for Statistical Computing. https://www.R-project.org/.

[47] Kim G.D. (2021). Harmine Hydrochloride Triggers G2/M Cell Cycle Arrest and Apoptosis in HCT116 Cells through ERK and PI3K/AKT/mTOR Signaling Pathways. Prev. Nutr. Food Sci..

[48] Ock C.W., Kim G.D. (2021). Harmine Hydrochloride Mediates the Induction of G2/M Cell Cycle Arrest in Breast Cancer Cells by Regulating the MAPKs and AKT/FOXO3a Signaling Pathways. Molecules.

[49] Mota N.S.R.S., Kviecinski M.R., Felipe K.B., Grinevicius V.M.A.S., Siminski T., Almeida G.M., Zeferino R.C., Pich C.T., Filho D.W., Pedrosa R.C. (2020). β-carboline alkaloid harmine induces DNA damage and triggers apoptosis by a mitochondrial pathway: Study in silico, in vitro and in vivo. Int. J. Funct. Nutr..

[50] Zheng J., Zeng L., Tang M., Lin H., Pi C., Xu R., Cui X. (2021). Novel Ferrocene Derivatives Induce G0/G1 Cell Cycle Arrest and Apoptosis through the Mitochondrial Pathway in Human Hepatocellular Carcinoma. Int. J. Mol. Sci..

[51] Zeng L., Tang M., Pi C., Zheng J., Gao S., Chabanne T., Chauvin R., Cheng W., Lin H., Xu R. (2021). Novel Ferrocene Derivatives Induce Apoptosis through Mitochondria-Dependent and Cell Cycle Arrest via PI3K/Akt/mTOR Signaling Pathway in T Cell Acute Lymphoblastic Leukemia. Cancers.

[52] Singh D., Hazra C.K., Malakar C.C., Pandey S.K., Kaith B.S., Singh V. (2018). Indium-Mediated Domino Allylation-Lactonisation Approach: Diastereoselective Synthesis of β-Carboline C-3 Tethered α-Methylene γ-Butyrolactones. ChemistrySelect.

[53] Szabó T., Hazai V., Volk B., Simig G., Milen M. (2019). First total synthesis of the β-carboline alkaloids trigonostemine A, trigonostemine B and a new synthesis of pityriacitrin and hyrtiosulawesine. Tetrahedron Lett..

