# Supplementary material for: Harmicens, Novel Harmine and Ferrocene Hybrids: Design, Synthesis and Biological Activity
Source: Int J Mol Sci. 2022 Aug 18;23(16):9315. doi: 10.3390/ijms23169315 (PMC9408872; doi:10.3390/ijms23169315)
Supplement: Supplementary file 1 [file ijms-23-09315-s001.zip › ijms-1866349-supplementary.pdf]

Table S1. IR, ^{1}H and ^{13}C NMR spectroscopic data for alkynes **2** and **9**

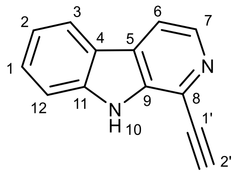

2

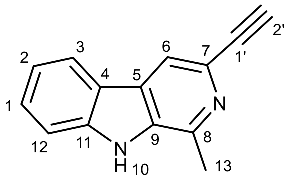

9

Compd.	IR (ATR, ν/cm^{-1})	^{1}H NMR (DMSO-d_6, δ ppm)	^{13}C NMR (DMSO-d_6, δ ppm)
**2**	3289, 3154, 3061, 2111, 1626, 1562, 1498, 1452, 1424, 1387, 1321, 1273, 1246, 1151, 1081, 1066, 939, 874, 856, 837, 783, 748, 684, 631, 569, 513	11.79 (s, 1H, 10), 8.34 (d, 1H, 7, *J* = 5.2 Hz), 8.26 (d, 1H, 3, *J* = 7.9 Hz), 8.17 (d, 1H, 6, *J* = 5.2 Hz), 7.64 (d, 1H, 12, *J* = 8.2 Hz), 7.60-7.56 (m, 1H, 1), 7.30–7.26 (m, 1H, 2), 4.77 (s, 1H, 2')	140.83 (8), 138.66 (7), 137.67 (11), 128.67 (1), 128.14 (9), 125.42 (5), 122.02 (3), 120.69 (4), 119.81 (2), 115.32 (6), 112.33 (12), 84.94 (1'), 30.68 (2')
**9**	3306, 3143, 2104, 1622, 1597, 1563, 1499, 1446, 1375, 1338, 1315, 1281, 1247, 1178, 1147, 1014, 961, 899, 877, 776, 740, 654, 625, 587, 504, 457	11.83 (s, 1H, 10), 8.25–8.22 (m, 1H, 3), 8.21 (s, 1H, 6), 7.62–7.59 (m, 1H, 12), 7.57–7.53 (m, 1H, 1), 7.27–7.23 (m, 1H, 2), 4.03 (s, 1H, 2'), 2.74 (s, 3H, 13)	142.83 (7), 140.70 (8), 134.06 (11), 129.54 (9), 128.30 (1), 127.01 (5), 122.05 (3), 120.72 (4), 119.78 (2), 117.22 (6), 112.15 (12), 84.85 (1'), 76.72 (2'), 20.33 (13)

Table S2. ^{1}H and ^{13}C NMR spectroscopic data for azide **15**

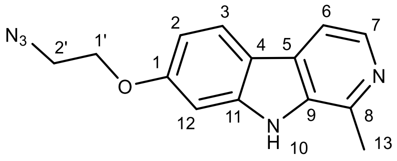

15

Compd.	^{1}H NMR (DMSO-d_6, δ ppm)	^{13}C NMR (DMSO-d_6, δ ppm)
**15**	11.44 (s, 1H, 10), 8.16 (d, 1H, 7, *J* = 5.3 Hz), 8.08 (d, 1H, 3, *J* = 8.6 Hz), 7.82 (d, 1H, 6, *J* = 5.2 Hz), 7.04 (d, 1H, 12, *J* = 2.2 Hz), 6.87 (dd, 1H, 2, *J* = 8.6, 2.2 Hz), 4.30 (t, 2H, 1', *J* = 4.8 Hz), 3.72 (t, 2H, 2', *J* = 4.8 Hz), 2.73 (s, 3H, 13)	158.70 (1), 141.77 (8), 141.35 (11), 137.78 (7), 134.60 (9), 127.12 (5), 122.75 (3), 115.26 (4), 111.99 (6), 109.14 (2), 95.62 (12), 67.14 (1'), 49.64 (2'), 20.33 (13)

Table S3. ^{1}H and ^{13}C NMR spectroscopic data for ferrocene azide **19**

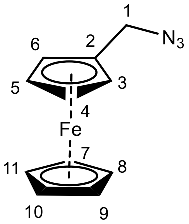

19

Compd.	^{1}H NMR (DMSO-d_6, δ ppm)	^{13}C NMR (DMSO-d_6, δ ppm)
**19**	4.29 (t, 2H, 3, 6, *J* = 1.8 Hz), 4.21 (t, 2H, 4, 5, *J* = 1.9 Hz), 4.20 (s, 2H, 1), 4.20 (s, 5H, 7–11)	81.78 (2), 68.62 (3, 6), 68.58 (7–11), 68.44 (4, 5), 50.02 (1)

Table S4. Analytical and MS data for harmicenes **20–24**

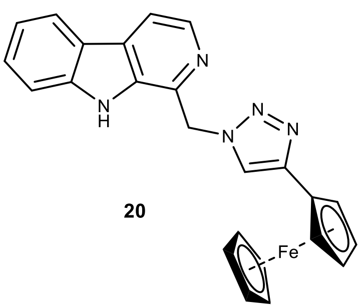

20

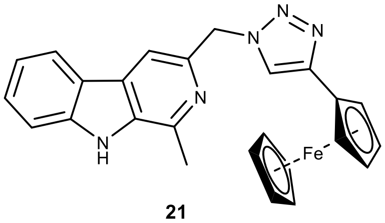

21

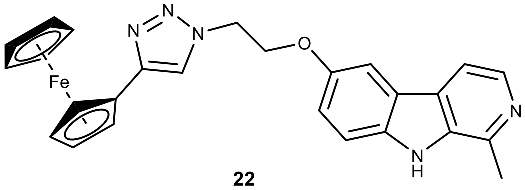

22

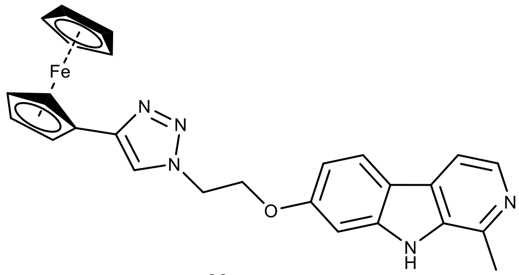

23

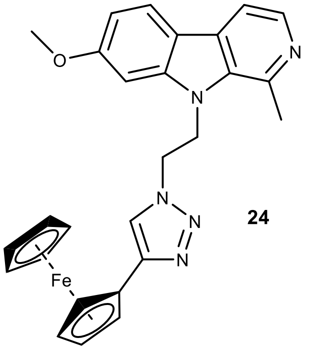

24

Compd.	Yield (%)	t_t (°C)	Molecular formula	M_r	MS (*m/z*)	CHN
**20**	78	252–255.5	$C_{24}H_{19}FeN_5$	433.30	434.9 $(M+1)^+$	C, 66.53; H, 4.42; N, 16.16
**21**	62	242.5–244	$C_{25}H_{21}FeN_5$	447.32	448.9 $(M+1)^+$	C, 67.13; H, 4.73; N, 15.66
**22**	37	201.5–204	$C_{26}H_{23}FeN_5O$	477.35	477.9 $(M+1)^+$	C, 65.42; H, 4.86; N, 14.67
**23**	77	228–229.5	$C_{26}H_{23}FeN_5O$	477.35	477.9 $(M+1)^+$	C, 65.42; H, 4.86; N, 14.67
**24**	63	218–220	$C_{27}H_{25}FeN_5O$	491.38	491.9 $(M+1)^+$	C, 66.00; H, 5.13; N, 14.25

Table S5. IR, ^{1}H and ^{13}C NMR spectroscopic data for harmicenes **20–24**

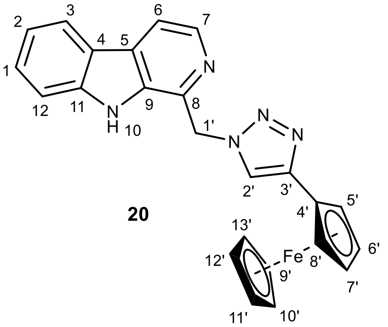

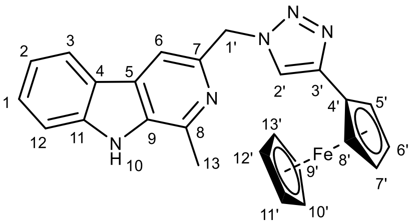

21

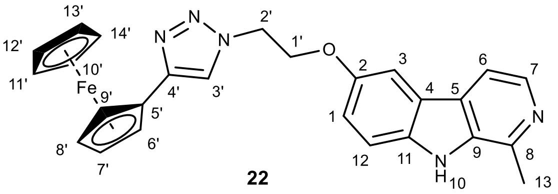

22

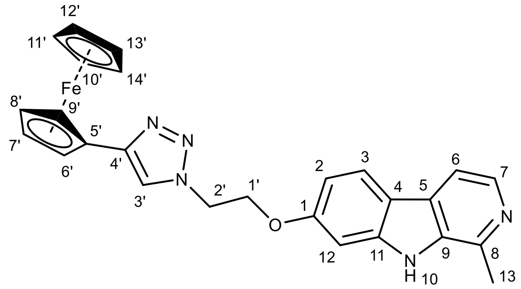

23

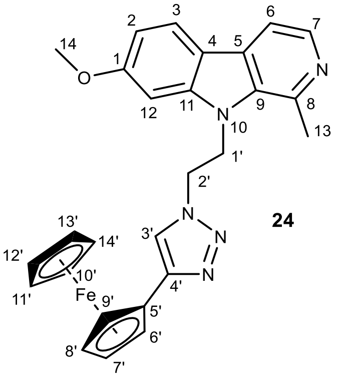

24

Compd.	IR (ATR, *ν*/cm^{-1})	^{1}H NMR (DMSO-d_6, δ ppm)	^{13}C NMR (DMSO-d_6, δ ppm)
**20**	3249, 3138, 3094, 2987, 2953, 1625, 1585, 1567, 1497, 1455, 1430, 1389, 1321, 1236, 1212, 1189, 1094, 1058, 998, 875, 816, 794, 750, 730, 622, 592, 500	11.95 (s, 1H, 10), 8.31 (d, 1H, 7, *J* = 5.2 Hz), 8.28–8.26 (m, 2H, 3, 2'), 8.13 (d, 1H, 6, *J* = 5.1 Hz), 7.70–7.68 (m, 1H, 12), 7.61–7.59 (m, 1H, 1), 7.30–7.27 (m, 1H, 2), 6.08 (s, 2H, 1'), 4.73 (t, 2H, 5', 8', *J* = 1.9 Hz), 4.28 (t, 2H, 6', 7', *J* = 1.8 Hz), 4.03 (s, 5H, 9'–13')	145.22 (8), 140.71 (11), 138.17 (3'), 137.86 (7), 133.85 (9), 128.71 (5), 128.54 (2'), 121.91 (3), 121.57 (1), 120.76 (4), 119.64 (2), 114.82 (6), 112.09 (12), 76.04 (4'), 69.23 (9'–13'), 68.21 (5', 8'), 66.34 (6', 7'), 51.24 (1')
**21**	3148, 3101, 2993, 2891, 1737, 1627, 1566, 1506, 1456, 1443, 1351, 1328, 1251, 1219, 1086, 1057, 1019, 1000, 874, 815, 729, 680, 587, 506, 487	11.66 (s, 1H, 10), 8.23 (s, 1H, 6), 8.16 (d, 1H, 3, *J* = 7.9 Hz), 7.90 (s, 1H, 2'), 7.60–7.59 (m, 1H, 12), 7.55–7.52 (m, 1H, 1), 7.24–7.21 (m, 1H, 2), 5.76 (s, 2H, 1'), 4.74 (t, 2H, 5', 8', *J* = 1.9 Hz), 4.29 (t, 2H, 6', 7', *J* = 1.9 Hz), 4.03 (s, 5H, 9'–13'), 2.76 (s, 3H, 13)	145.37 (8), 142.95 (7), 142.05 (11), 140.77 (3'), 133.94 (9), 128.08 (2'), 127.63 (5), 121.64 (3), 121.06 (1), 120.91 (4), 119.44 (2), 112.09 (6), 111.42 (12), 76.09 (4'), 69.26 (9'–13'), 68.21 (5', 8'), 66.32 (6', 7'), 55.10 (1'), 20.38 (13)
**22**	3133, 2949, 2872, 1583, 1568, 1497, 1458, 1286, 1210, 1105, 1041, 988, 878, 818, 705, 621, 504	11.39 (s, 1H, 10), 8.29 (s, 1H, 3'), 8.15 (d, 1H, 7, *J* = 5.3 Hz), 7.87 (d, 1H, 6, *J* = 5.3 Hz), 7.78 (d, 1H, 12, *J* = 2.5 Hz), 7.48 (d, 1H, 12, *J* = 8.8 Hz), 7.16 (dd, 1H, 1, *J* = 8.8, 2.5 Hz), 4.82 (t, 2H, 1', *J* = 5.1 Hz), 4.73 (t, 2H, 6', 9', *J* = 1.9 Hz), 4.53 (t, 2H, 2', *J* = 5.2	151.85 (2), 145.24 (8), 142.24 (4'), 136.98 (7), 135.52 (11), 135.10 (9), 126.63 (4), 121.35 (5), 121.28 (3'), 118.14 (1), 112.77 (6), 112.64 (12), 105.13 (3), 76.05 (5'), 69.22 (10'–14'), 68.21 (6', 9'), 67.14 (1'), 66.34 (7', 8'), 49.24 (2'), 20.40 (13)

		Hz), 4.30 (t, 2H, 7', 8', *J* = 1.8 Hz), 4.01 (s, 5H, 10'–14'), 2.72 (s, 3H, 13)	
**23**	3125, 3077, 2959, 2850, 2772, 1623, 1566, 1443, 1426, 1301, 1278, 1240, 1185, 1108, 1053, 1042, 977, 874, 822, 810, 743, 720, 693, 638, 587, 570, 522, 513, 495, 483	11.44 (s, 1H, 10), 8.30 (s, 1H, 3'), 8.14 (d, 1H, 7, *J* = 5.3 Hz), 8.05 (d, 1H, 3, *J* = 8.6 Hz), 7.79 (d, 1H, 6, *J* = 5.3 Hz), 7.03 (d, 1H, 12, *J* = 2.2 Hz), 6.85 (dd, 1H, 2, *J* = 8.6, 2.2 Hz), 4.83 (t, 2H, 1', *J* = 5.1 Hz), 4.73 (t, 2H, 6', 9', *J* = 1.9 Hz), 4.55 (t, 2H, 2', *J* = 5.1 Hz), 4.30 (t, 2H, 7', 8', *J* = 1.8 Hz), 4.01 (s, 5H, 10'–14'), 2.72 (s, 3H, 13)	158.62 (1), 145.25 (8), 141.72 (4'), 141.35 (11), 137.76 (7), 134.59 (9), 127.07 (5), 122.71 (3'), 121.34 (3), 115.33 (4), 111.98 (6), 109.17 (2), 95.73 (12), 76.01 (5'), 69.23 (10'–14'), 68.22 (6', 9'), 66.57 (1'), 66.35 (7', 8'), 49.09 (2'), 20.36 (13)
**24**	2968, 1621, 1565, 1445, 1404, 1339, 1253, 1222, 1158, 1182, 1136, 1096, 1041, 1021, 970, 929, 877, 821, 807, 641, 590, 545, 501, 475	8.17 (d, 1H, 7, *J* = 5.2 Hz), 8.04 (d, 1H, 3, *J* = 8.5 Hz), 7.88 (d, 1H, 6, *J* = 5.2 Hz), 7.80 (s, 1H, 3'), 6.84–6.78 (m, 2H, 2, 12), 5.08 (t, 2H, 1', *J* = 5.6 Hz), 4.88 (t, 2H, 2', *J* = 5.6 Hz), 4.49 (t, 2H, 6', 9', *J* = 1.9 Hz), 4.22 (t, 2H, 7', 8', *J* = 1.9 Hz), 3.86–3.85 (m, 8H, 14, 10'–14'), 2.90 (s, 3H, 13)	160.51 (1), 145.15 (4'), 142.76 (8), 140.67 (11), 138.12 (7), 134.68 (9), 128.73 (5), 122.23 (3'), 121.79 (3), 114.10 (4), 112.26 (6), 109.81 (2), 92.93 (12), 75.78 (5'), 68.09 (10'–14'), 68.06 (6', 9'), 66.26 (7', 8'), 55.37 (14), 49.71 (1'), 44.47 (2'), 23.18 (13)

Table S6. Analytical and MS data for harmicenes **25–29**

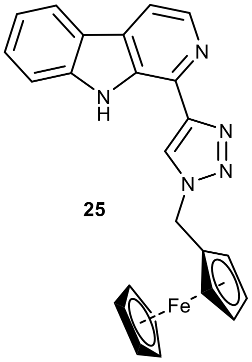

25

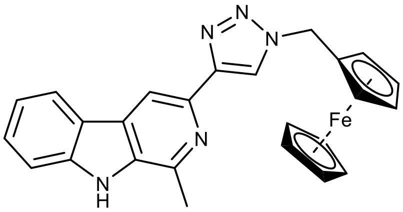

26

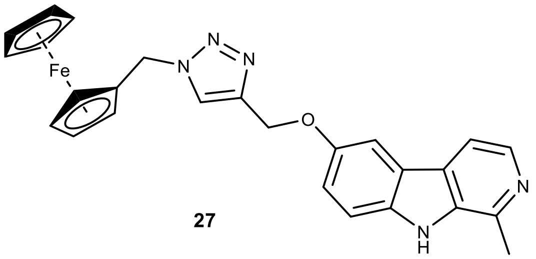

27

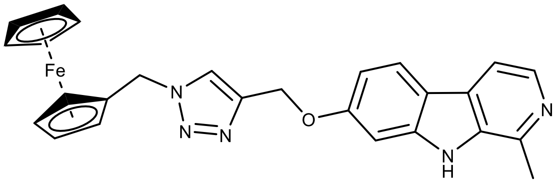

28

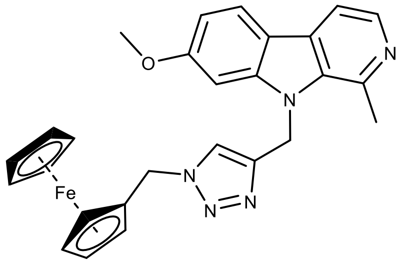

29

Compd.	Yield (%)	t_t (°C)	Molecular formula	M_r	MS (m/z)	CHN
**25**	51	229–230	$C_{24}H_{19}FeN_5$	433.30	434.1 $(M+1)^+$	C, 66.53; H, 4.42; N, 16.16
**26**	45	183–186	$C_{25}H_{21}FeN_5$	447.32	447.9 $(M+1)^+$	C, 67.13; H, 4.73; N, 15.66
**27**	68	244.5–248	$C_{26}H_{23}FeN_5O$	477.35	478.1 $(M+1)^+$	C, 65.42; H, 4.86; N, 14.67
**28**	60	242–244	$C_{26}H_{23}FeN_5O$	477.35	478.1 $(M+1)^+$	C, 65.42; H, 4.86; N, 14.67
**29**	30	187–190	$C_{27}H_{25}FeN_5O$	491.38	492.1 $(M+1)^+$	C, 66.00; H, 5.13; N, 14.25

Table S7. IR, ^{1}H and ^{13}C NMR spectroscopic data for harmicenes **25–29**

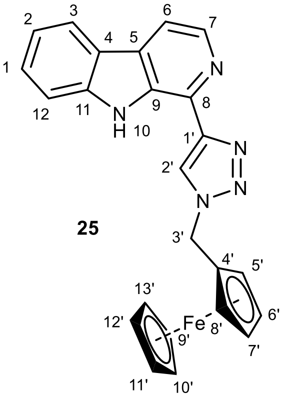

25

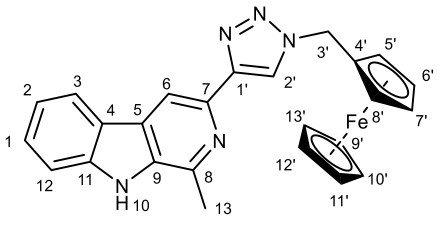

26

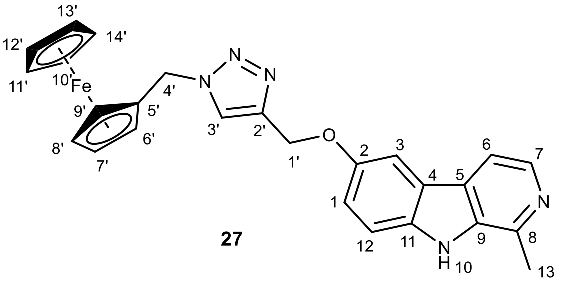

27

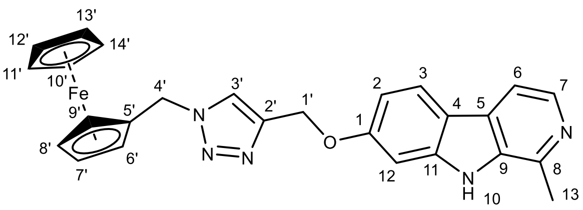

28

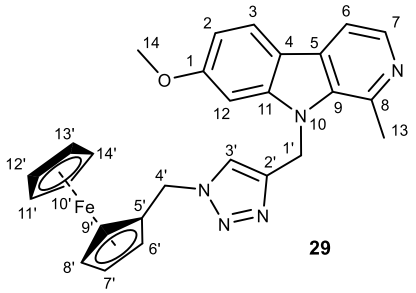

29

Compd.	IR (ATR, ν/cm^{-1})	^{1}H NMR (DMSO-d_6, δ ppm)	^{13}C NMR (DMSO-d_6, δ ppm)
**25**	3385, 3172, 3105, 3065, 2991, 1625, 1576, 1489, 1452, 1437, 1419, 1351, 1314, 1282, 1254, 1239, 1152, 1103, 1000, 829, 807, 749, 629, 591, 548, 499, 479	11.53 (s, 1H, 10), 8.78 (s, 1H, 2'), 8.38 (d, 1H, 7, *J* = 5.2 Hz), 8.26–8.24 (m, 1H, 3), 8.11 (d, 1H, 6, *J* = 5.1 Hz), 7.91–7.90 (m, 1H, 12), 7.57–7.54 (m,, 1H, 1), 7.27–7.25 (m, 1H, 2), 5.49 (s, 2H, 3'), 4.46 (t, 2H, 5', 8', *J* = 1.9 Hz), 4.24 (s, 5H, 9'–13'), 4.22 (t, 2H, 6', 7', *J* = 1.9 Hz)	147.60 (8), 141.15 (11), 137.83 (7), 133.68 (9), 131.74 (1'), 129.05 (4), 128.18 (2'), 122.94 (1), 121.47 (3), 120.40 (5), 119.51 (2), 114.11 (6), 113.23 (12), 82.36 (4'), 68.74 (5', 8'), 68.69 (9'–13'), 68.48 (6', 7'), 49.33 (3')
**26**	3361, 2920, 2851, 1734, 1626, 1574, 1496, 1455, 1431, 1373, 1338, 1303, 1277, 1234, 1172, 1104, 1049, 1024, 1000, 924, 889, 818, 788, 775, 704, 631, 558, 584, 503, 478	11.69 (s, 1H, 10), 8.58 (s, 1H, 2'), 8.41 (s, 1H, 6), 8.29 (d, 1H, 3, *J* = 7.9 Hz), 7.60 (d, 1H, 12, *J* = 8.1 Hz), 7.54 (t, 1H, 1, *J* = 7.6 Hz), 7.24 (t, 1H, 2, *J* = 7.4 Hz), 5.39 (s, 2H, 3'), 4.45 (t, 2H, 5', 8', *J* = 1.9 Hz), 4.23 (s, 5H, 9'–13'), 4.21 (t, 2H, 6', 7', *J* = 1.9 Hz), 2.80 (s, 3H, 13)	148.61 (7), 141.93 (11), 140.82 (8), 139.95 (9), 133.96 (1'), 128.02 (2'), 127.73 (4), 121.97 (1), 121.27 (5), 121.25 (3), 119.36 (2), 112.01 (6), 108.31 (12), 82.45 (4'), 68.86 (5', 8'), 68.68 (9'–13'), 68,44 (6', 7'), 49.05 (3'), 20.48 (13)
**27**	3631, 3137, 3081, 2950, 1637, 1603, 1583, 1567, 1499, 1480, 1451, 1411, 1283, 1211, 1107, 1069, 1054, 1040, 1027, 849, 821, 765, 620, 504	11.37 (s, 1H, 10), 8.24 (s, 1H, 3'), 8.16 (d, 1H, 7, *J* = 5.3 Hz), 7.89–7.88 (m, 2H, 3, 6), 7.49 (d, 1H, 12, *J* = 8.8 Hz), 7.21 (dd, 1H, 1, *J* = 8.9, 2.5 Hz), 5.32 (s, 2H, 1'), 5.21 (s, 2H, 4'), 4.32 (t, 2H, 6', 9', *J* = 1.8 Hz), 4.16–4.14 (m, 7H, 7', 8', 10'–14'), 2.73 (s, 3H, 13)	151.88 (2), 142.95 (2'), 142.19 (8), 136.96 (7), 135.42 (11), 135.08 (9), 126.69 (4), 124.09 (3'), 121.34 (5), 118.39 (1), 112.73 (6), 112.67 (12), 105.20 (3), 82.49 (5'), 68.62 (10'-14'), 68.59 (6', 9',), 68.31 (7', 8'), 61.88 (1'), 48.92 (4'), 20.40 (13)
**28**	3344, 3072, 2869, 2785, 1738, 1626, 1567, 1488, 1444, 1377, 1325, 1305, 1290, 1278, 1177, 1142, 1110, 1058, 1142, 1011, 965, 828, 813, 781, 661, 639, 598, 572, 505, 480	11.45 (s, 1H, 10), 8.23 (s, 1H, 3'), 8.15 (d, 1H, 7, *J* = 5.2 Hz), 8.05 (d, 1H, 3, *J* = 8.6 Hz), 7.80 (d, 1H, 6, *J* = 5.3 Hz), 7.16 (d, 1H, 12, *J* = 2.2 Hz), 6.89 (dd, 1H, 2, *J* = 8.7, 2.3 Hz), 5.33 (s, 2H, 1'), 5.25 (s, 2H, 4'), 4.34 (t, 2H, 6', 9', *J* = 1.9 Hz), 4.18–4.16 (m, 7H, 7', 8', 10'–14'), 2.72 (s, 3H, 13)	158.74 (1), 142.71 (8), 141.76 (2'), 141.33 (11), 137.74 (7), 134.58 (9), 127.13 (5), 124.15 (3'), 122.63 (3), 115.11 (4), 111.95 (6), 109.46 (2), 95.86 (12), 82.41 (5'), 68.66 (6', 9'), 68.64 (10'–14'), 68.36 (7', 8'), 61.45 (1'), 48.98 (4'), 20.32 (13)

**29**	3135, 3090, 2929, 1709, 1623, 1564, 1499, 1449, 1408, 1325, 1252, 1227, 1193, 1174, 1106, 1042, 1000, 913, 815, 764, 732, 638, 596, 550, 481	8.16 (d, 1H, 7, *J* = 5.2 Hz), 8.07 (d, 1H, 3, *J* = 8.5 Hz), 7.98 (s, 1H, 3'), 7.86 (d, 1H, 6, *J* = 5.2 Hz), 7.32 (d, 1H, 12, *J* = 2.2 Hz), 6.87 (dd, 1H, 2, *J* = 8.5, 2.2 Hz), 5.86 (s, 2H, 1'), 5.21 (s, 2H, 4'), 4.23 (t, 2H, 6', 9', *J* = 1.8 Hz), 4.12 (t, 2H, 7', 8', *J* = 1.8 Hz), 4.06 (s, 5H, 10'–14'), 3.88 (s, 3H, 14), 3.03 (s, 3H, 13)	160.55 (1), 143.81 (2'), 142.67 (8), 141.13 (11), 138.05 (7), 134.66 (9), 128.54 (5), 122.55 (3'), 122.38 (3), 114.46 (4), 112.26 (6), 109.46 (2), 94.05 (12), 82.59 (5'), 68.54 (10'–14'), 68.44 (6', 9'), 68.21 (7', 8'), 55.62 (14), 48.77 (4'), 23.24 (13)

Table S8. Analytical and MS data for harmicenes **30–33**

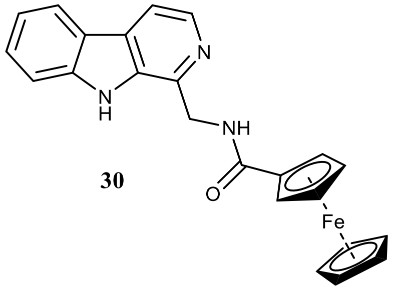

30

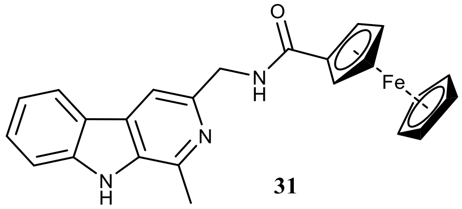

31

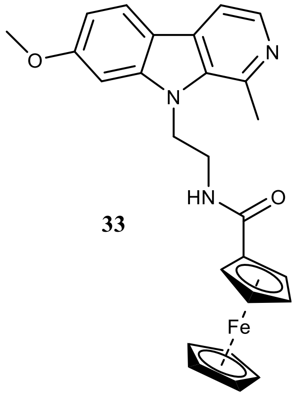

33

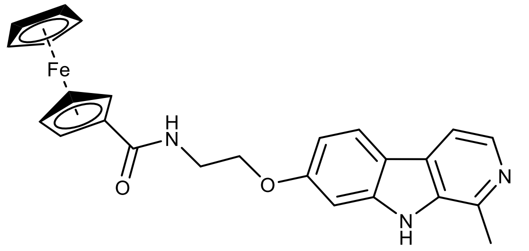

32

Compd.	Yield (%)	t_t (°C)	Molecular formula	M_r	MS (*m/z*)	CHN
**30**	48	210–213	$C_{23}H_{19}FeN_3O$	409.27	410.0 $(M+1)^+$	C, 67.50; H, 4.68; N, 10.27
**31**	39	240.5–243	$C_{24}H_{21}FeN_3O$	423.30	424.0 $(M+1)^+$	C, 68.10; H, 5.00; N, 9.93
**32**	42	243–245.5	$C_{25}H_{23}FeN_3O_2$	453.32	454.0 $(M+1)^+$	C, 66.24; H, 5.11; N, 9.27
**33**	50	225–227	$C_{26}H_{25}FeN_3O_2$	467.35	468.1 $(M+1)^+$	C, 66.82; H, 5.39; N, 8.99

Table S9. IR, ^{1}H and ^{13}C NMR spectroscopic data for harmicenes **30–33**

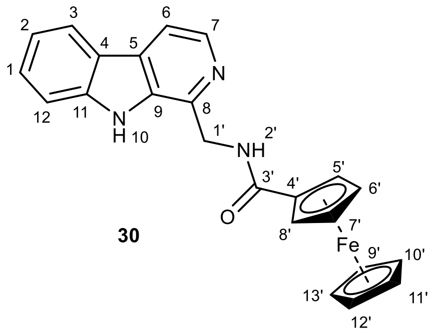

30

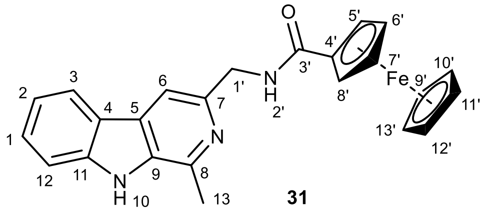

31

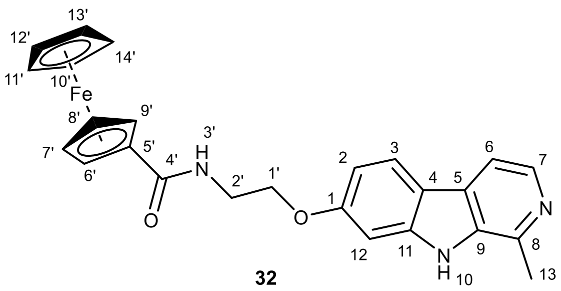

32

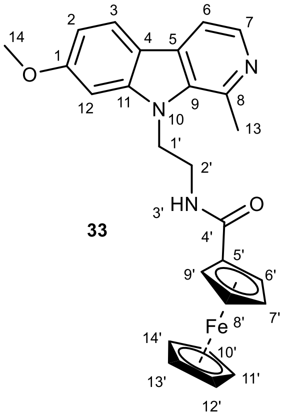

33

Compd.	IR (ATR, ν/cm^{-1})	^{1}H NMR (DMSO-d_6, δ ppm)	^{13}C NMR (DMSO-d_6, δ ppm)
**30**	3623, 3467, 3085, 3050, 2962, 2928, 1711, 1649, 1622, 1531, 1503, 1444, 1408, 1375, 1340, 1300, 1249, 1229, 1195, 138, 1123, 1106, 1041, 1017, 914, 819, 801, 634, 598, 558	11.49 (s, 1H, 10), 8.58 (t, 1H, 2', *J* = 6.0 Hz), 8.31 (d, 1H, 7, *J* = 5.2 Hz), 8.23 (d, 1H, 3, *J* = 7.8 Hz), 8.05 (d, 1H, 6, *J* = 5.2 Hz), 7.68 (d, 1H, 12, *J* = 8.2 Hz), 7.56 (t, 1H, 1, *J* = 7.6 Hz), 7.25 (t, 1H, 2, *J* = 7.5 Hz), 4.88–4.87 (m, 4H, 1', 5', 8'), 4.35 (t, 2H, 6', 7', *J* = 1.9 Hz), 4.07 (s, 5H, 9'–13')	169.83 (3'), 142.64 (8), 140.17 (11), 137.35 (7), 133.55 (9), 128.10 (3), 127.74 (5), 121.74 (1), 120.93 (4), 119.37 (2), 113.86 (6), 111.99 (12), 76.11 (4'), 70.10 (5', 8'), 69.31 (9'–13'), 68.30 (6', 7'), 41.73 (1')
**31**	3324, 3226, 1634, 1574, 1532, 1498, 1447, 1380, 1348, 1297, 1248, 1104, 1026, 901, 814, 755, 738, 629, 528, 482	11.51 (s, 1H, 10), 8.47 (t, 1H, 2', *J* = 6.1 Hz), 8.12 (d, 1H, 3, *J* = 7.9 Hz), 7.89 (s, 1H, 6), 7.57 (d, 1H, 12, *J* = 8.2 Hz), 7.52–7.48 (m, 1H, 1), 7.21–7.17 (m, 1H, 2), 4.90 (t, 2H, 5', 8', *J* = 1.9 Hz), 4.60 (d, 2H, 1', *J* = 6.0 Hz), 4.37 (t, 2H, 6', 7', *J* = 1.9 Hz), 4.20 (s, 5H, 9'–13'), 2.78 (s, 3H, 13)	168.94 (3'), 147.17 (7), 141.14 (8), 140.76 (11), 133.49 (9), 127.78 (3), 127.66 (5), 121.35 (1), 120.95 (4), 119.16 (2), 111.98 (12), 109.47 (6), 76.73 (4'), 69.96 (5', 8'), 69.25 (9'–13'), 68.28 (6', 7'), 44.31 (1'), 20.27 (13)

**32**	3216, 1716, 1622, 1551, 1451, 1422, 1376, 1312, 1278, 1236, 1217, 1105, 960, 842, 801, 741, 641, 605, 505,476	11.41 (s, 1H, 10), 8.14 (d, 1H, 7, *J* = 5.3 Hz,), 8.07–8.05 (m, 2H, 3', 3), 7.79 (d, 1H, 6, *J* = 5.3 Hz), 7.07 (d, 1H, 12, *J* = 2.4 Hz), 6.90 (dd, 1H, 2, *J* = 8.7, 2.2 Hz), 4.83 (t, 2H, 6', 9', *J* = 2.0 Hz), 4.35 (t, 2H, 7', 8', *J* = 1.9 Hz), 4.23 (t, 2H, 1', *J* = 5.7 Hz), 4.13 (s, 5H, 10'–14'), 3.63 (q, 2H, 2', *J* = 5.7 Hz), 2.71 (s, 3H, 13)	169.36 (4'), 159.31 (1), 141.89 (8), 141.28 (11), 137.74 (7), 134.55 (9), 127.17 (5), 122.69 (3), 114.98 (4), 111.93 (6), 109.22 (2), 95.38 (12), 76.31 (5'), 70.01 (6', 9'), 69.40 (10'–14'), 68.22 (7', 8'), 66.40 (1'), 38.49 (2'), 20.33 (13)
**33**	3623, 3467, 3085, 3050, 2962, 2928, 1711, 1649, 1622, 1531, 1503, 1444, 1408, 1375, 1340, 1300, 1249, 1229, 1195, 1177, 1138, 1123, 1107, 1041, 1016, 914, 819, 801, 634, 598, 558	8.17 (d, 1H, 7, *J* = 5.1 Hz), 8.10–8.07 (m, 2H, 3, 3'), 7.88 (d, 1H, 6, *J* = 5.1 Hz), 7.32 (d, 1H, 12, *J* = 2.2 Hz), 6.88 (dd, 1H, 2, *J* = 8.5, 2.1 Hz), 4.69 (t, 2H, 6', 9', *J* = 1.9 Hz), 4.66 (t, 2H, 1', *J* = 7.1 Hz), 4.34 (t, 2H, 7', 8', *J* = 1.9 Hz), 4.07 (s, 5H, 10'–14'), 3.91 (s, 3H, 14), 3.60 (q, 2H, 2', *J* = 6.7 Hz), 3.04 (s, 3H, 13)	169.75 (4'), 160.52(1), 143.00 (8), 140.66 (11), 137.82 (7), 134.67 (9), 128.43 (5), 122.39 (3), 114.28 (4), 112.26 (6), 109.20 (2), 93.78 (12), 76.40 (5'), 69.93 (6', 9'), 69.32 (10'–14'), 68.08 (7', 8'), 55.50 (14), 43.56 (1'), 39.10 (2'), 23.14 (13)

Table S10. Analytical and MS data for harmicenes **34–37**

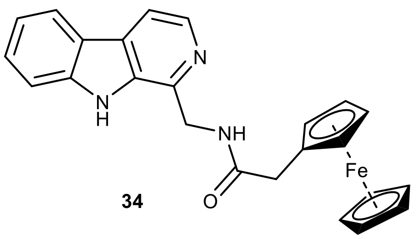

34

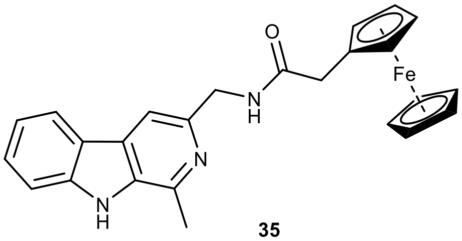

35

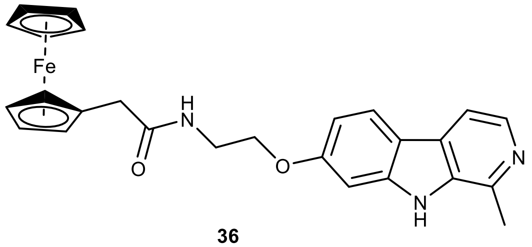

36

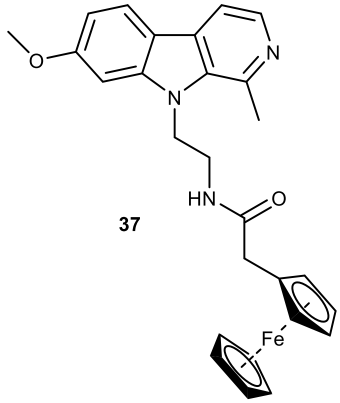

37

Compd.	Yield (%)	t_t (°C)	Molecular formula	M_r	MS (*m/z*)	CHN
**34**	34	222–224.5	$C_{24}H_{21}FeN_3O$	423.30	424.1 $(M+1)^+$	C, 68.10; H, 5.00; N, 9.93
**35**	39	247.5–250	$C_{25}H_{23}FeN_3O$	437.32	438.0 $(M+1)^+$	C, 68.66; H, 5.30; N, 9.61
**36**	58	176–177.5	$C_{26}H_{25}FeN_3O_2$	467.35	468.1 $(M+1)^+$	C, 66.82; H, 5.39; N, 8.99
**37**	69	170.5–172	$C_{27}H_{27}FeN_3O_2$	481.38	482.2 $(M+1)^+$	C, 67.37; H, 5.65; N, 8.73

Table S11. IR, ^{1}H and ^{13}C NMR spectroscopic data for harmicenes **34**–**37**

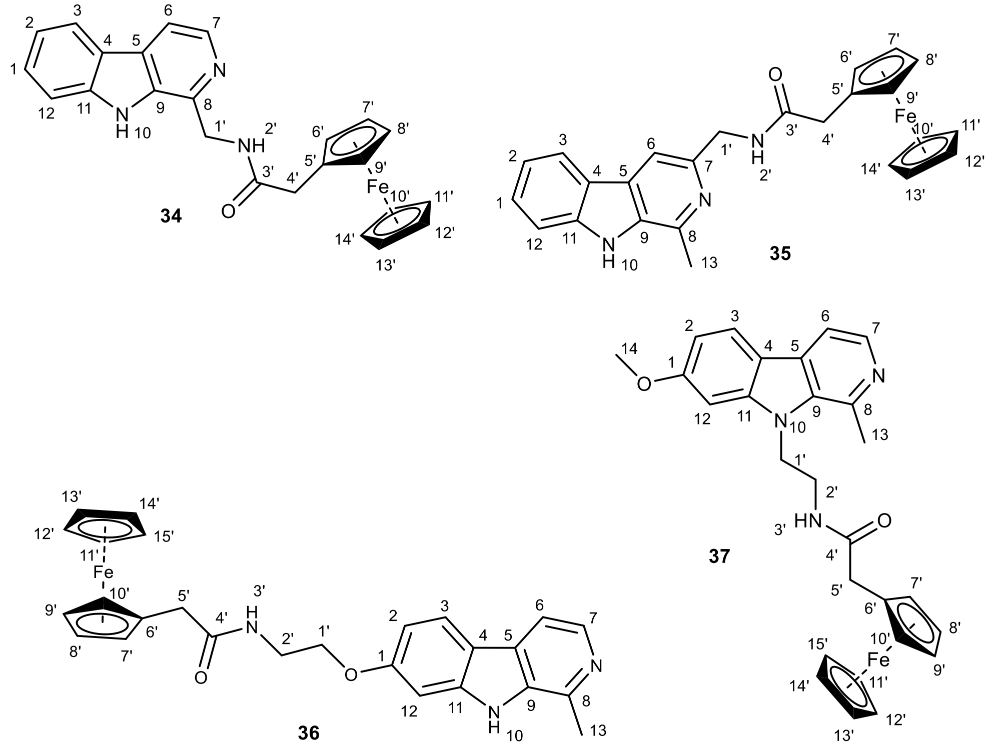

Compd.	IR (ATR, ν/cm^{-1})	^{1}H NMR (DMSO-d_6, δ ppm)	^{13}C NMR (DMSO-d_6, δ ppm)
**34**	3333, 3219, 3168, 3099, 2995, 2898, 1651, 1568, 1521, 1502, 1445, 1436, 1413, 1361, 1321, 1285, 1248, 1151, 1106, 1045, 1026, 999, 878, 820, 740, 677, 595, 564, 504	11.50 (s, 1H, 10), 8.55 (t, 1H, 2', *J* = 5.4 Hz), 8.29 (d, 1H, 7, *J* = 5.2 Hz), 8.22 (d, 1H, 3, *J* = 7.8 Hz), 8.04 (d, 1H, 6, *J* = 5.2 Hz), 7.63 (d, 1H, 12, *J* = 8.2 Hz), 7.55 (t, 1H, 1, *J* = 7.6 Hz), 7.25 (t, 1H, 2, *J* = 7.4 Hz), 4.76 (d, 2H, 1', *J* = 5.4 Hz), 4.22 (t, 2H, 6', 9', *J* = 1.9 Hz), 4.05 (m, 7H, 7', 8', 10'–14'), 3.26 (s, 2H, 4')	170.47 (3'), 141.60 (8), 140.36 (11), 137.36 (7), 133.50 (9), 128.12 (3), 127.74 (5), 121.73 (1), 120.87 (4), 119.39 (2), 113.89 (6), 112.05 (12), 82.66 (5'), 68.58 (6', 9'), 68.41 (10'–14'), 67.11 (7', 8'), 41.52 (1'), 36.33 (4')

**35**	3404, 3191, 3081, 2944, 1649, 1621, 1570, 1522, 1471, 1455, 1421, 1312, 1247, 1267, 1136, 1103, 1038, 1023, 997, 925, 826, 803, 753, 741, 693, 621, 584, 543, 500, 482	11.48 (s, 1H, 10), 8.42 (t, 1H, 2', *J* = 5.9 Hz), 8.10 (d, 1H, 3, *J* = 7.8 Hz), 7.73 (s, 1H, 6), 7.56 (d, 1H, 12, *J* = 8.2 Hz), 7.52–7.50 (m, 1H, 1), 7.21 (t, 1H, 2, *J* = 7.4 Hz), 4.46 (d, 2H, 1', *J* = 5.8 Hz), 4.27 (t, 2H, 6', 9', *J* = 1.9 Hz), 4.12 (s, 5H, 10'–14'), 4.10 (t, 2H, 7', 8', *J* = 1.9 Hz), 3.24 (s, 2H, 4'), 2.73 (s, 3H, 13)	169.93 (3'), 146.33 (7), 141.16 (8), 140.71 (11), 133.44 (9), 127.78 (3), 127.66 (5), 121.49 (1), 120.98 (4), 119.11 (2), 111.94 (12), 109.54 (6), 83.05 (5'), 68.63 (6', 9'), 68.45 (10'–14'), 67.15 (7', 8'), 44.28 (1'), 36.72 (4'), 20.28 (13)
**36**	3222, 3055, 2875, 1656, 1630, 1539, 1484, 1434, 1378, 1324, 1299, 1273, 1238, 1174, 1134, 1106, 1073, 1037, 1023, 1002, 961, 924, 871, 814, 776, 738, 661, 634, 590, 567, 484	11.40 (s, 1H, 10), 8.19 (t, 1H, 3', *J* = 5.5 Hz), 8.14 (d, 1H, 7, *J* = 5.3 Hz), 8.05 (d, 1H, 3, *J* = 8.6 Hz), 7.80 (d, 1H, 6, *J* = 5.3 Hz), 7.02 (d, 1H, 12, *J* = 2.2 Hz), 6.85 (dd, 1H, 2, *J* = 8.6, 2.2 Hz), 4.20 (t, 2H, 7', 10', *J* = 1.9 Hz), 4.13 (t, 2H, 1', *J* = 5.5 Hz), 4.11 (s, 5H, 11'–15'), 4.05 (t, 2H, 8', 9', *J* = 1.9 Hz), 3.51 (q, 2H, 2', *J* = 5.6 Hz), 3.16 (s, 2H, 5'), 2.72 (s, 3H, 13)	170.25 (4'), 159.17 (1), 141.84 (8), 141.29 (11), 137.75 (7), 134.56 (9), 127.16 (5), 122.63 (3), 115.00 (4), 111.93 (6), 109.30 (2), 95.40 (12), 82.72 (6'), 68.50 (7', 10'), 68.43 (11'–15'), 67.08 (8', 9'), 66.55 (1'), 38.40 (2'), 36.37 (5'), 20.35 (13)
**37**	3341, 2931, 1655, 1624, 1566, 1503, 1451, 1439, 1410, 1347, 1330, 1300, 1251, 1195, 1172, 1141, 1104, 1048, 1024, 929, 835, 813, 794, 639, 567, 496, 482	8.17 (d, 1H, 7, *J* = 5.1 Hz), 8.09 (d, 1H, 3, *J* = 8.6 Hz), 8.01 (t, 1H, 3', *J* = 6.0 Hz), 7.88 (d, 1H, 6, *J* = 5.1 Hz), 7.27 (d, 1H, 12, *J* = 2.2 Hz), 6.89 (dd, 1H, 2, *J* = 8.6, 2.2 Hz), 4.57 (t, 2H, 1', *J* = 7.1 Hz), 4.13 (t, 2H, 7', 10', *J* = 1.9 Hz), 4.11 (s, 5H, 11'–15'), 4.05 (t, 2H, 8', 9', *J* = 1.8 Hz), 3.92 (s, 3H, 14), 3.45 (q, 2H, 2', *J* = 6.7 Hz), 3.07 (s, 2H, 5'), 2.95 (s, 3H, 13)	170.58 (4'), 160.52 (1), 142.86 (8), 140.61 (11), 137.80 (7), 134.64 (9), 128.43 (5), 122.40 (3), 114.31 (4), 112.25 (6), 109.24 (2), 93.64 (12), 82.12 (6'), 68.72 (7', 10'), 68.46 (11'–15'), 67.21 (8', 9'), 55.52 (14), 43.37 (1'), 38.73 (2'), 36.65 (5'), 23.08 (13)

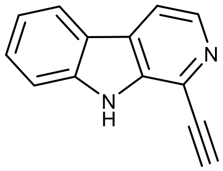
N
H
N

2

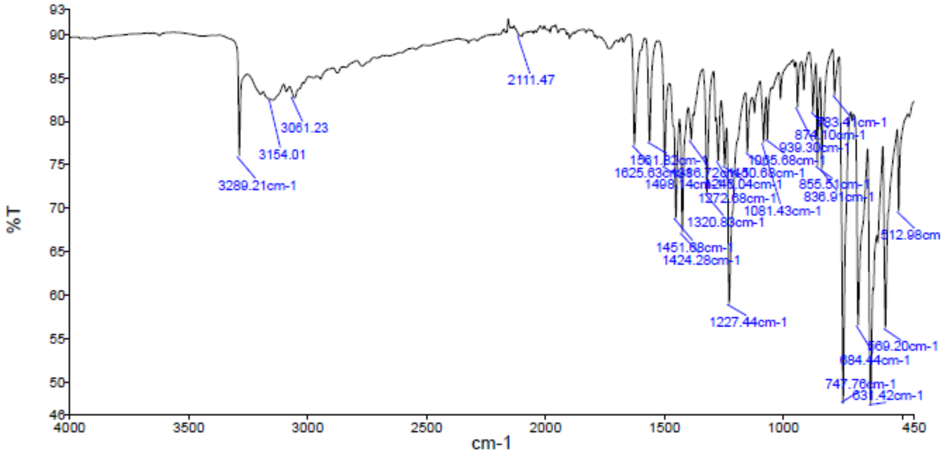
%T
cm-1
3289.21cm-1
3154.01
3061.23
2111.47
1451.68cm-1
1424.28cm-1
1320.83cm-1
1227.44cm-1
1081.43cm-1
939.30cm-1
855.51cm-1
836.91cm-1
512.98cm
669.20cm-1
684.44cm-1
747.76cm-1
631.42cm-1

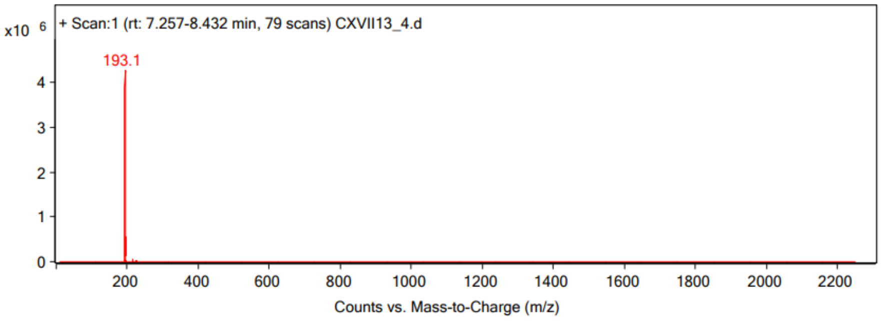
x10 6
+ Scan:1 (rt: 7.257-8.432 min, 79 scans) CXVII13_4.d
193.1
Counts vs. Mass-to-Charge (m/z)

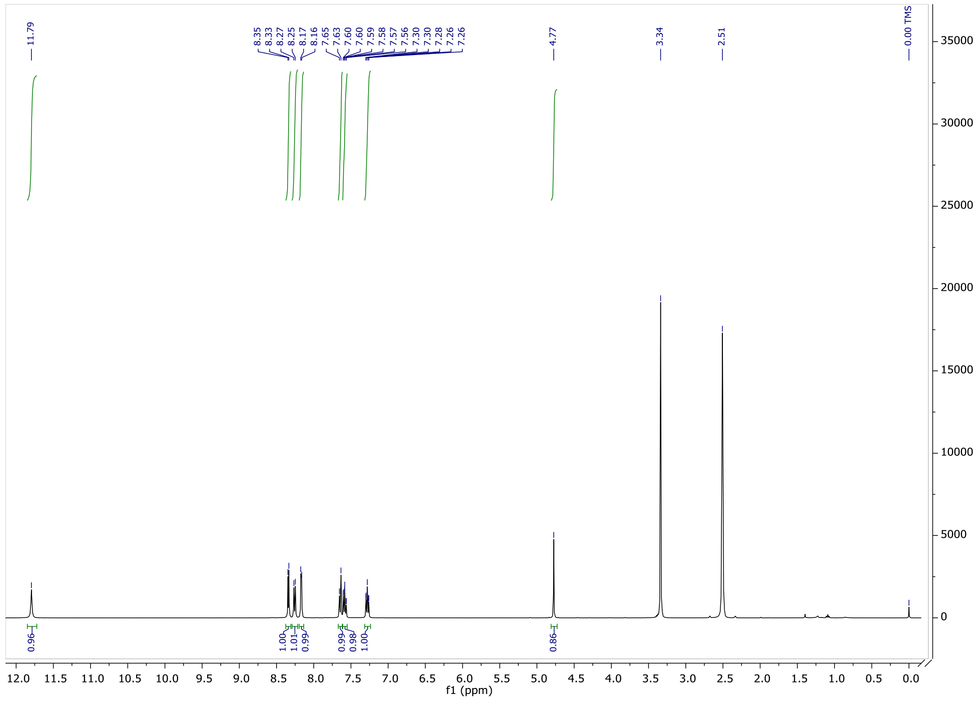
11.79
8.35
8.33
8.27
8.25
8.17
8.16
7.65
7.63
7.60
7.60
7.59
7.58
7.57
7.56
7.30
7.30
7.28
7.26
7.26
4.77
3.34
2.51
0.00 TMS
0.96
1.00
1.01
0.99
0.99
0.98
1.00
0.86
f1 (ppm)

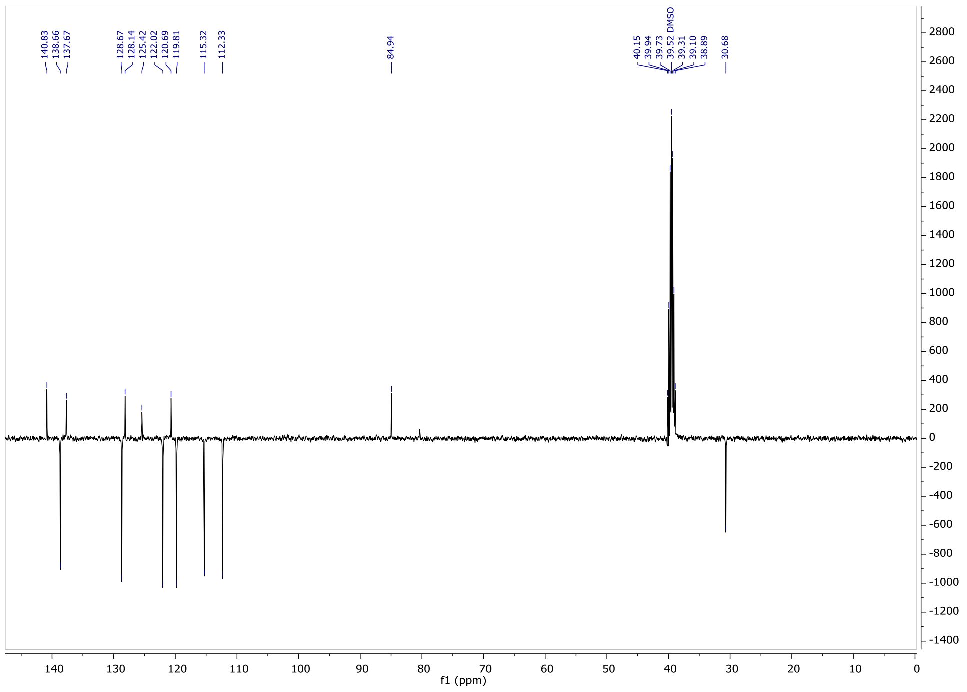
140.83
138.66
137.67
128.67
128.14
125.42
122.02
120.69
119.81
115.32
112.33
84.94
40.15
39.94
39.73
39.52 DMSO
39.31
39.10
38.89
30.68
f1 (ppm)

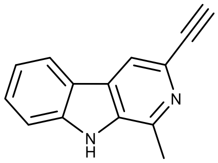

9

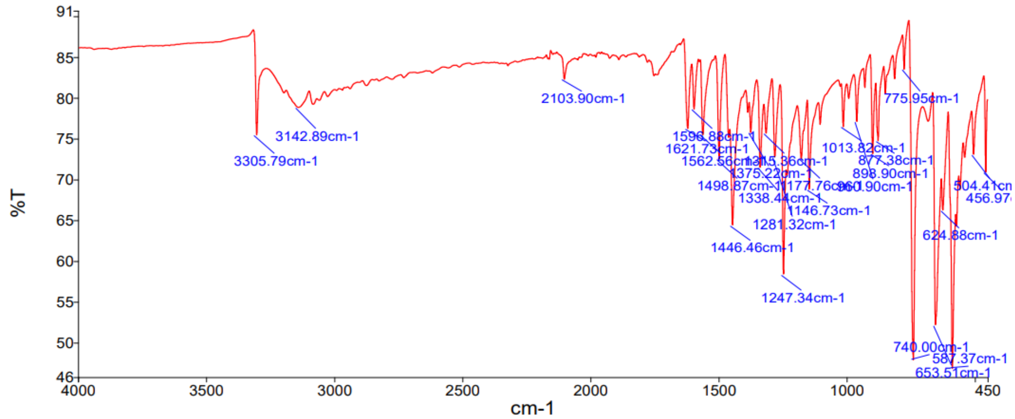
%T
cm-1
3305.79cm-1
3142.89cm-1
2103.90cm-1
1446.46cm-1
1247.34cm-1
775.95cm-1
740.00cm-1
587.37cm-1
653.51cm-1
624.88cm-1

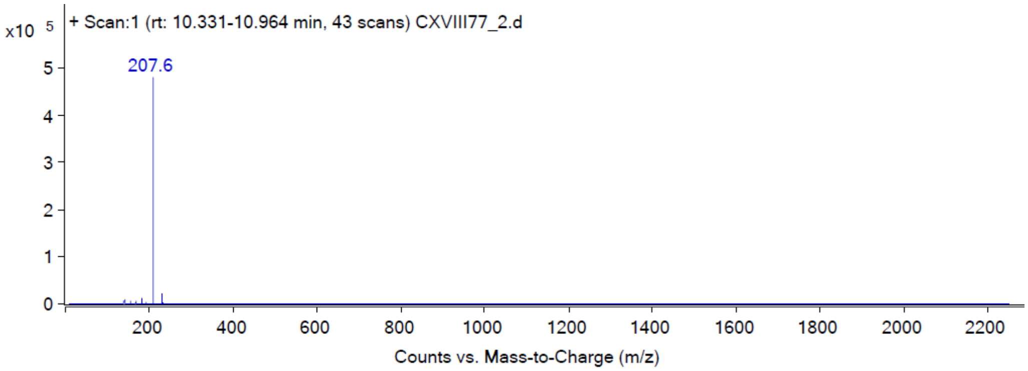
+ Scan:1 (rt: 10.331-10.964 min, 43 scans) CXVIII77_2.d
207.6
Counts vs. Mass-to-Charge (m/z)

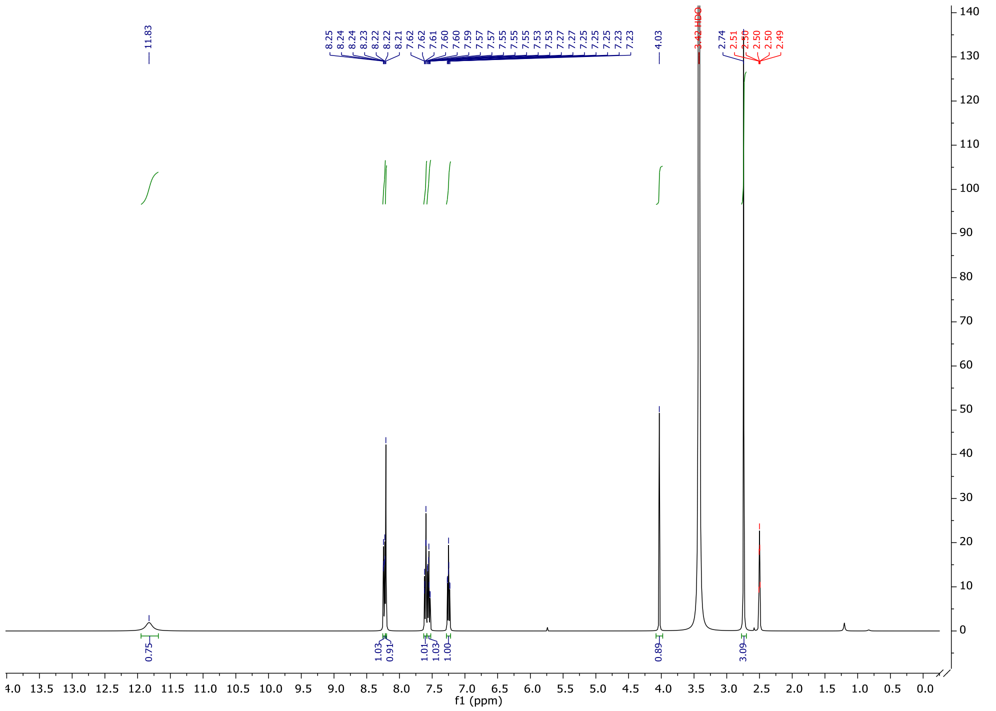

11.83
8.25 8.24 8.24 8.23 8.22 8.22 8.21 7.62 7.62 7.61 7.60 7.60 7.59 7.57 7.57 7.55 7.55 7.55 7.53 7.53 7.53 7.27 7.27 7.25 7.25 7.25 7.23 7.23
4.03
3.42 HDO
2.74 2.51 2.50 2.50 2.50 2.49
0.75
1.03 0.91
1.01 1.03 1.00
0.89
3.09
f1 (ppm)

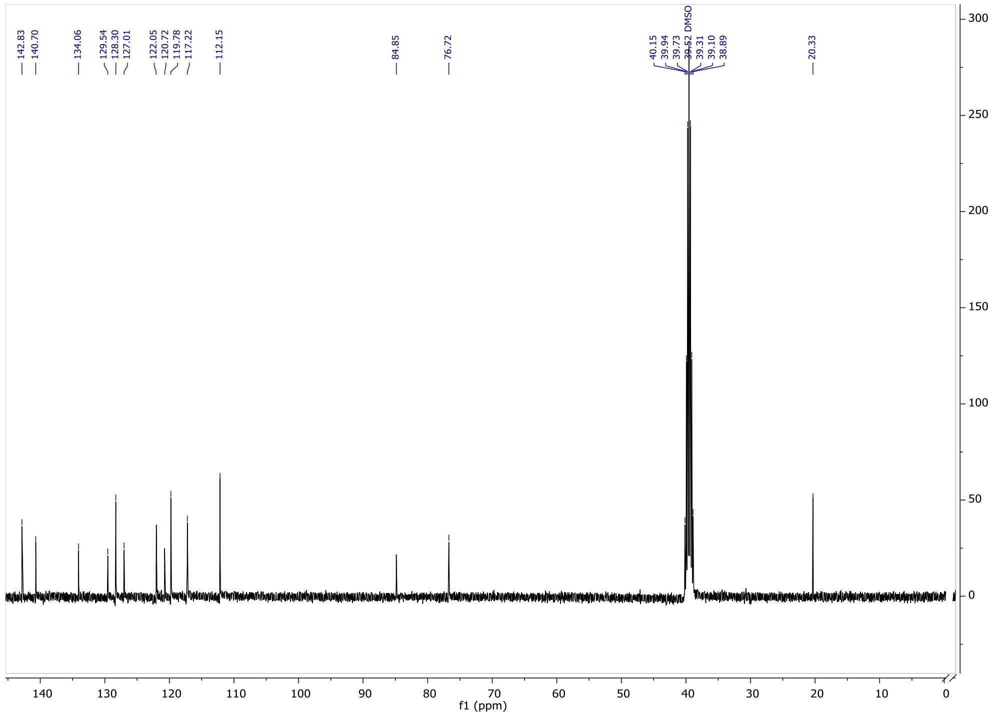

142.83 140.70 134.06 129.54 128.30 127.01 122.05 120.72 119.78 117.22 112.15
84.85
76.72
40.15 39.94 39.73 39.52 DMSO 39.31 39.10 38.89
20.33
f1 (ppm)

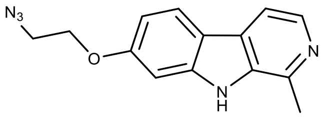
N3
O
N
N
H

15

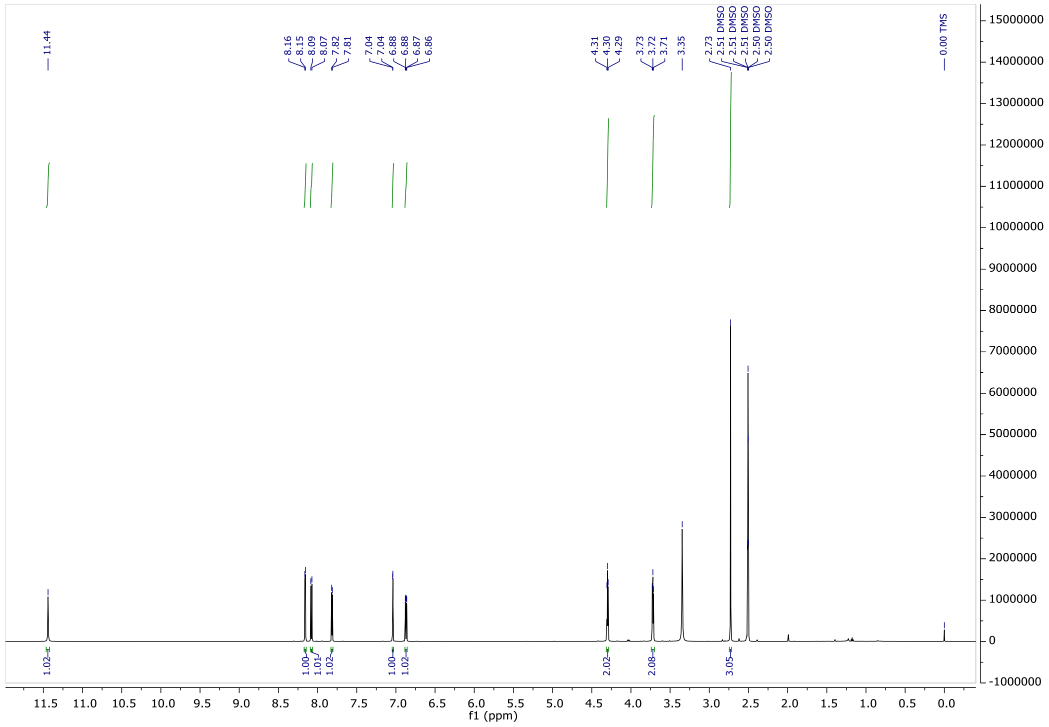
11.44
8.16
8.15
8.09
8.07
7.82
7.81
7.04
7.04
6.88
6.88
6.87
6.86
4.31
4.30
4.29
3.73
3.72
3.71
3.35
2.73
2.51 DMSO
2.51 DMSO
2.51 DMSO
2.50 DMSO
2.50 DMSO
0.00 TMS
1.02
1.00
1.01
1.02
1.00
1.02
2.02
2.08
3.05
f1 (ppm)

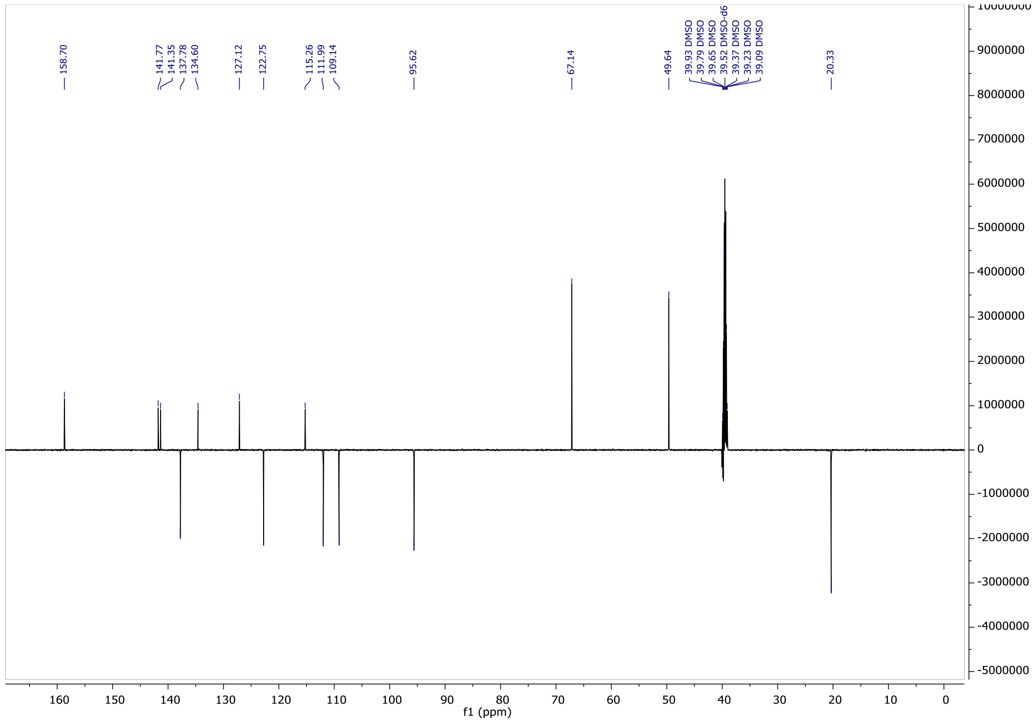
158.70
141.77
141.35
137.78
134.60
127.12
122.75
115.26
111.99
109.14
95.62
67.14
49.64
39.93 DMSO
39.79 DMSO
39.65 DMSO
39.52 DMSO-d6
39.37 DMSO
39.23 DMSO
39.09 DMSO
20.33
f1 (ppm)

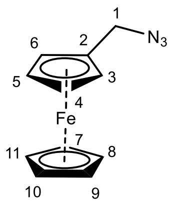

1
N3
2
6
5
3
4
Fe
7
11
8
10
9

19

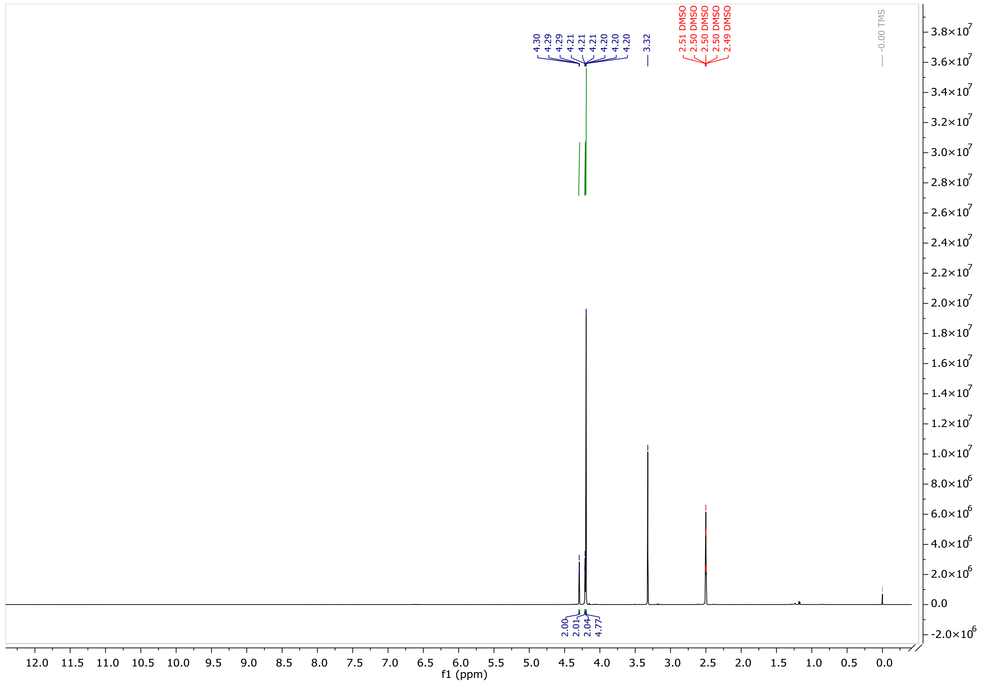

4.30
4.29
4.29
4.21
4.21
4.21
4.20
4.20
4.20
3.32
2.51 DMSO
2.50 DMSO
2.50 DMSO
2.50 DMSO
2.49 DMSO
-0.00 TMS
2.00
2.01
2.04
4.77
f1 (ppm)

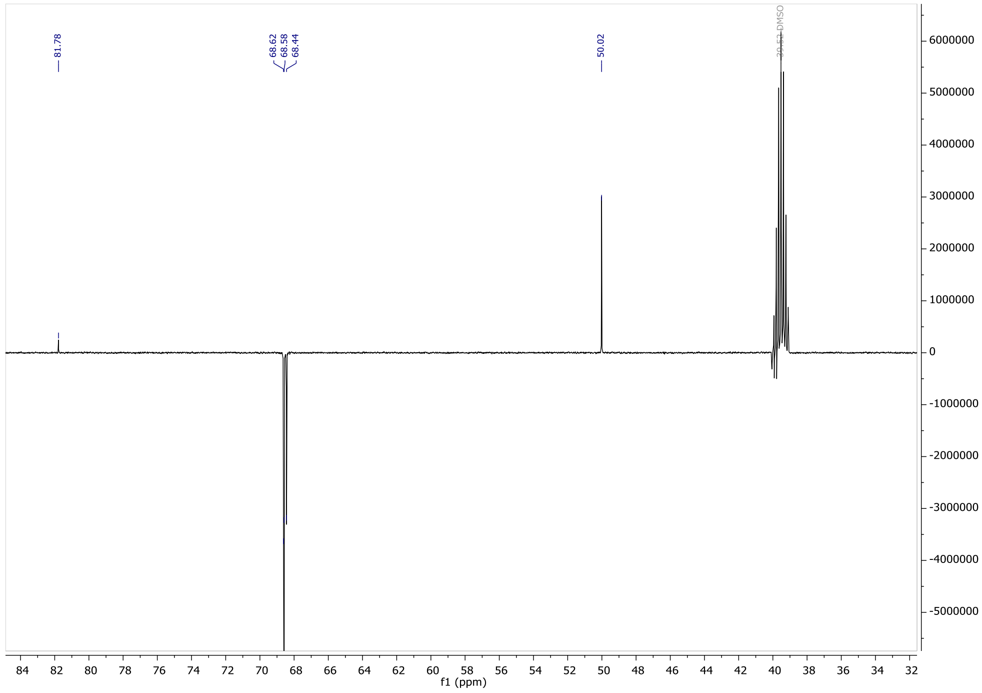

81.78
68.62
68.58
68.44
50.02
f1 (ppm)

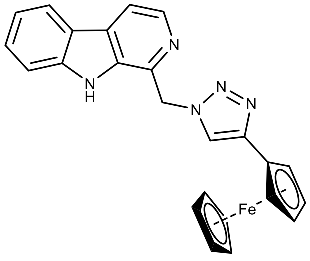

20

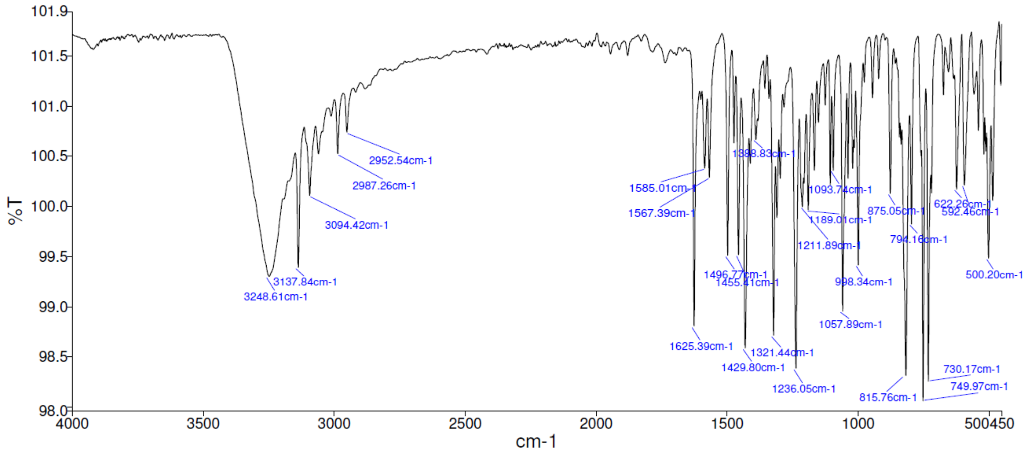

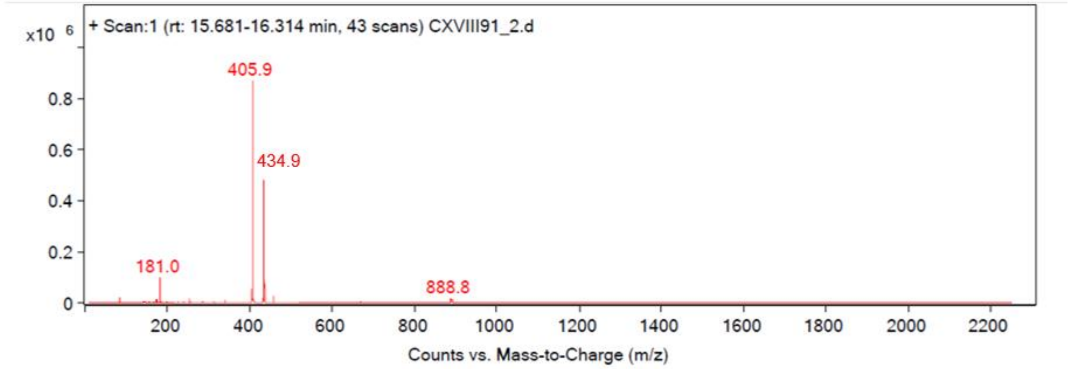

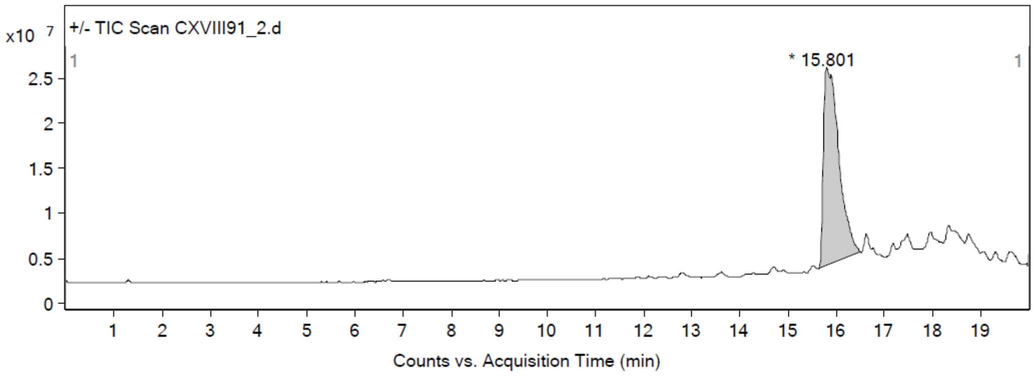

Integration Peak List

Peak	Start	RT	End	Height	Area	Area %
1	15.62	15.801	16.495	21919671.61	489627897.53	100

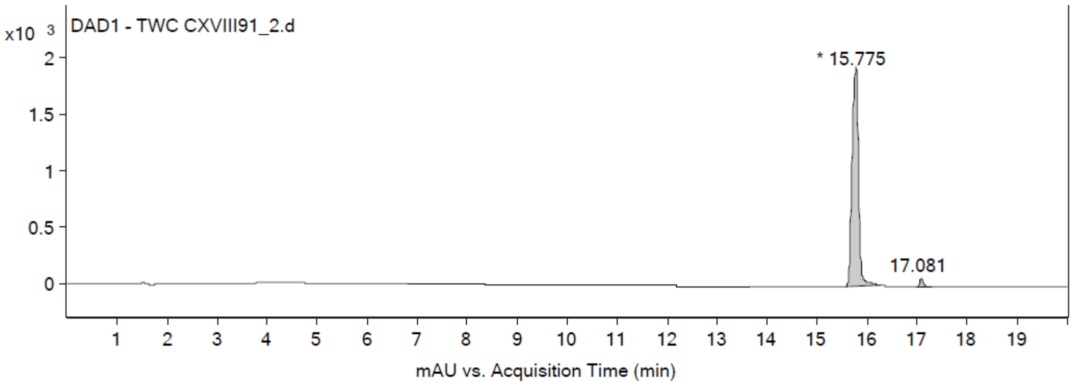

Integration Peak List

Peak	Start	RT	End	Height	Area	Area %
1	15.568	15.775	16.281	1945.65	17575.54	100
2	17.002	17.081	17.281	69.71	327.96	1.87

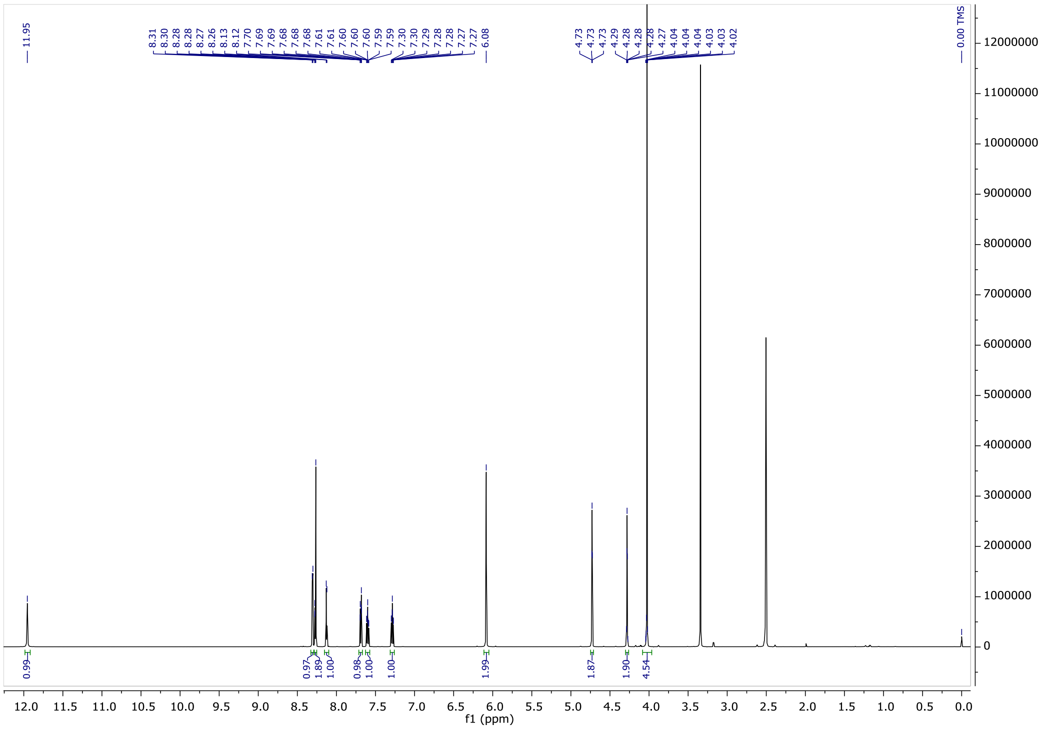

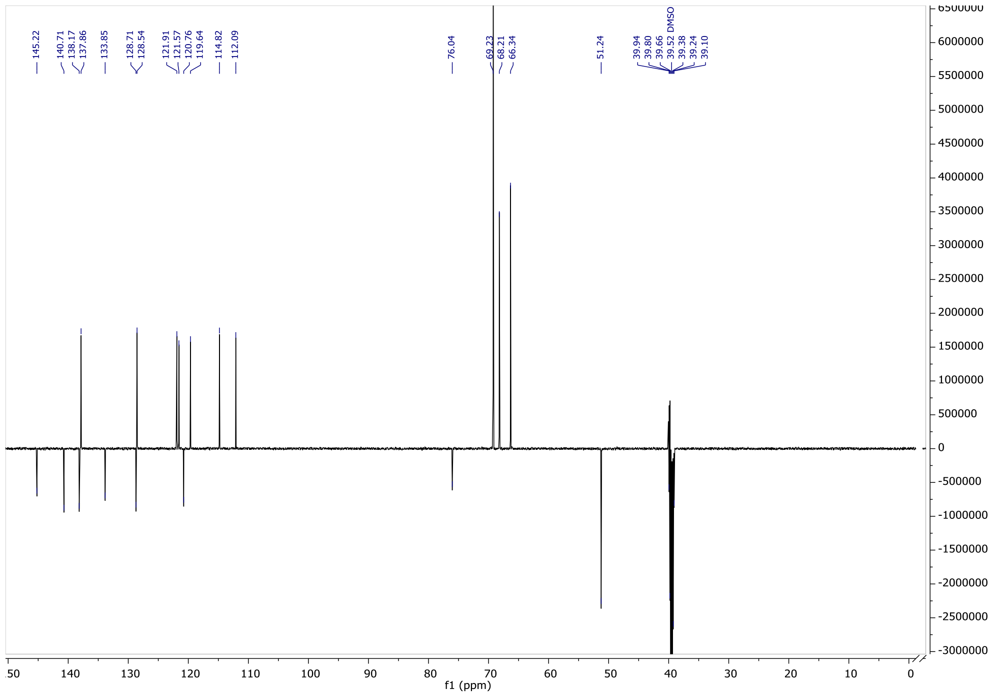
145.22
140.71
138.17
137.86
133.85
128.71
128.54
121.91
121.57
120.76
119.64
114.82
112.09
76.04
69.23
68.21
66.34
51.24
39.94
39.80
39.66
39.52 DMSO
39.38
39.24
39.10
f1 (ppm)

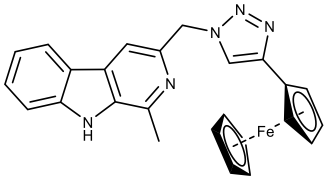
N
N
N
N
N
H
Fe

21

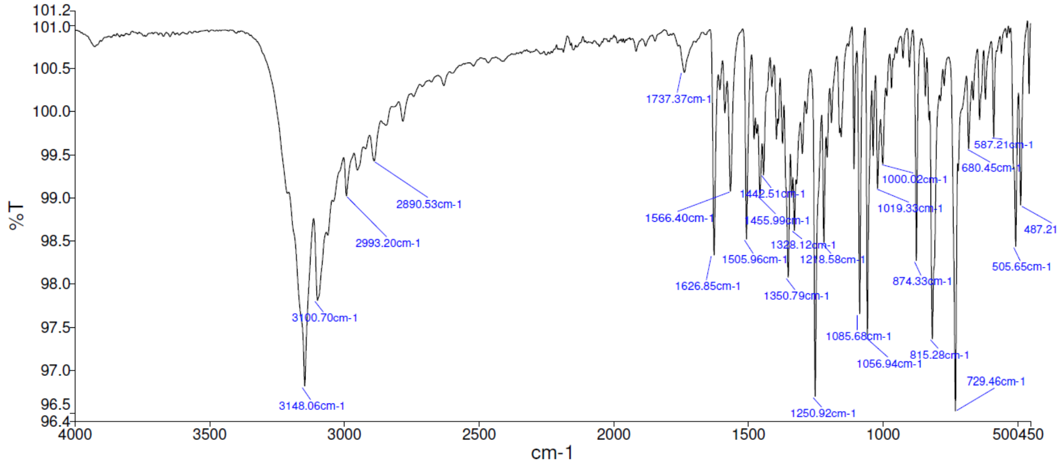
%T
cm-1
1737.37cm-1
2890.53cm-1
2993.20cm-1
3100.70cm-1
3148.06cm-1
1566.40cm-1
1442.51cm-1
1455.99cm-1
1328.12cm-1
1505.96cm-1
1218.58cm-1
1626.85cm-1
1350.79cm-1
1000.02cm-1
1019.33cm-1
874.33cm-1
1085.68cm-1
1056.94cm-1
815.28cm-1
729.46cm-1
1250.92cm-1
587.21cm-1
680.45cm-1
487.21
505.65cm-1

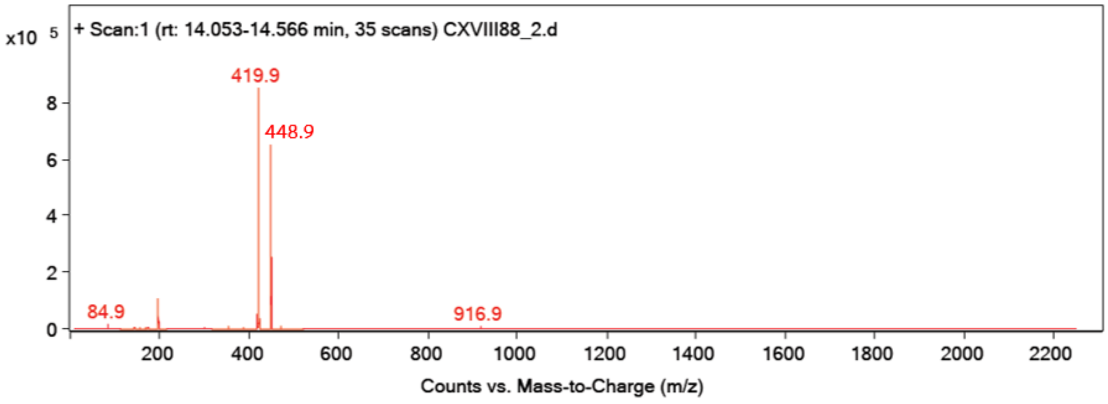

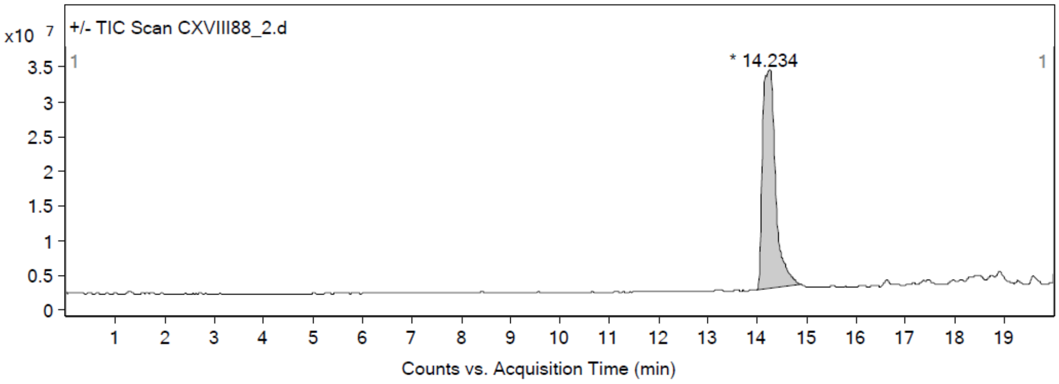

Integration Peak List

Peak	Start	RT	End	Height	Area	Area %
1	13.978	14.234	14.897	31661829.12	575842089.03	100

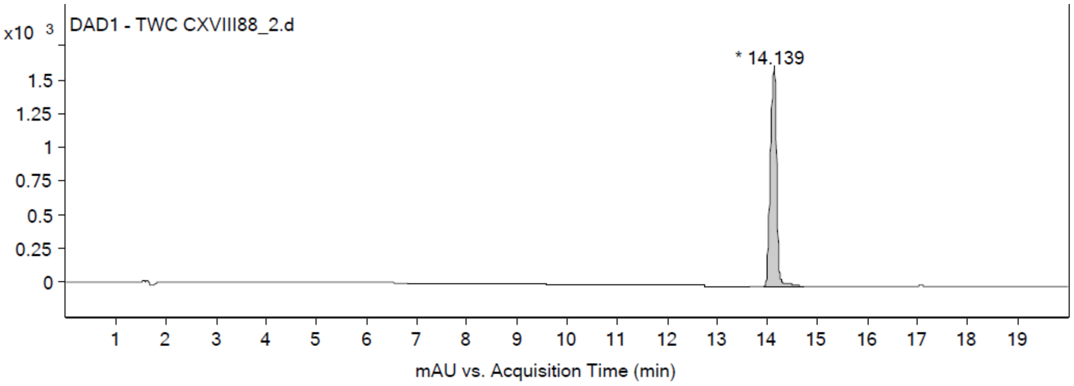

Integration Peak List

Peak	Start	RT	End	Height	Area	Area %
1	13.936	14.139	14.726	1625.52	14001.55	100

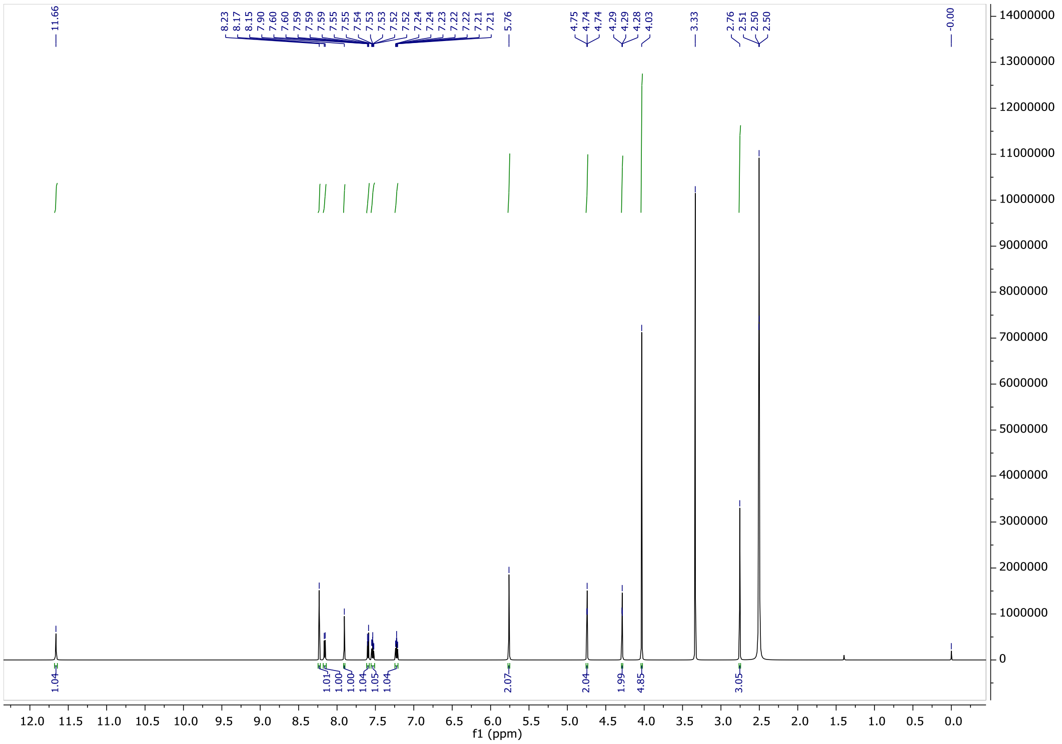

11.66
8.23 8.17 8.15 7.90 7.60 7.60 7.59 7.59 7.59 7.55 7.55 7.54 7.53 7.53 7.52 7.52 7.24 7.24 7.23 7.22 7.22 7.21 7.21
5.76
4.75 4.74 4.74 4.29 4.29 4.28 4.03
3.33
2.76 2.51 2.50 2.50
-0.00
1.04 1.01 1.00 1.00 1.04 1.05 1.04 2.07 2.04 1.99 4.85 3.05
f1 (ppm)

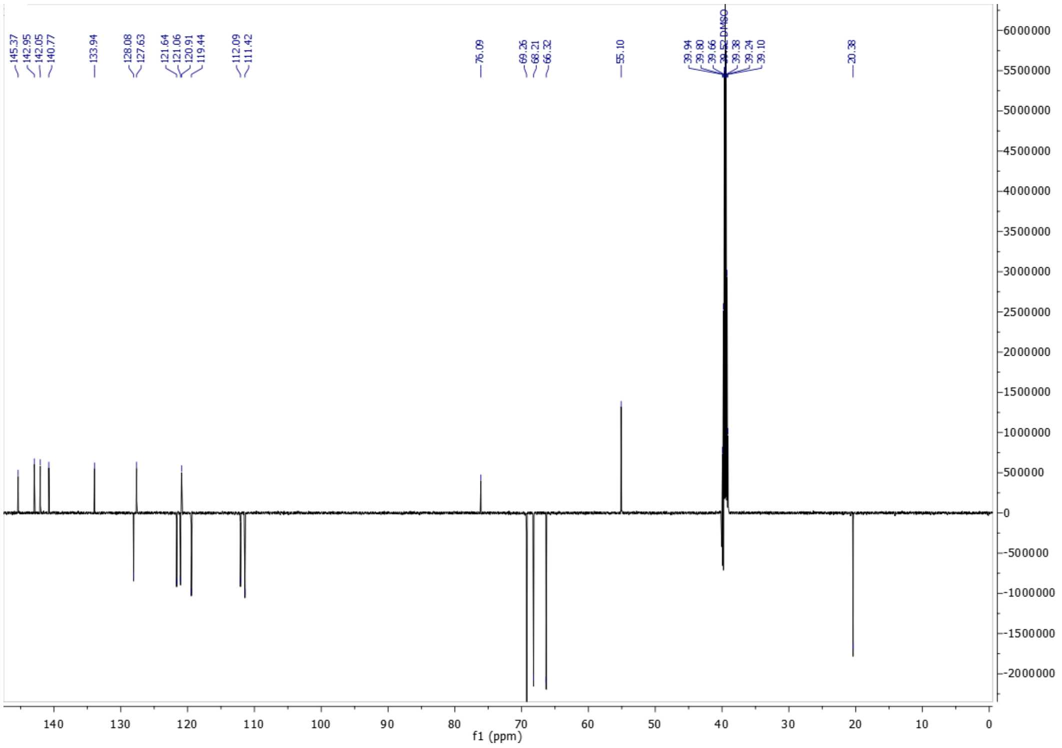

145.37 142.95 142.05 140.77 133.94 128.08 127.63 121.64 121.06 120.91 119.44 112.09 111.42
76.09
69.26 68.21 66.32
55.10
39.94 39.80 39.66 39.52 DMSO 39.38 39.24 39.10
20.38
f1 (ppm)

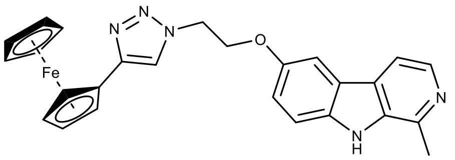

22

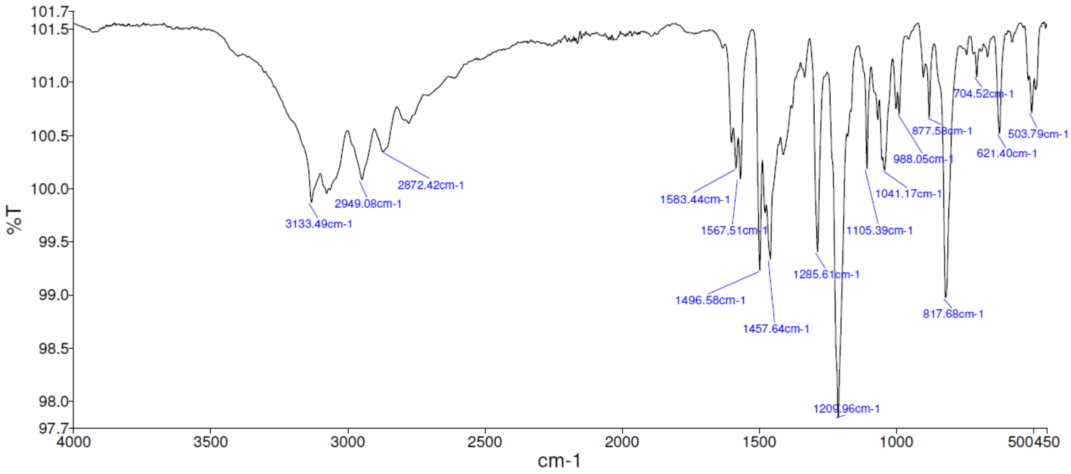

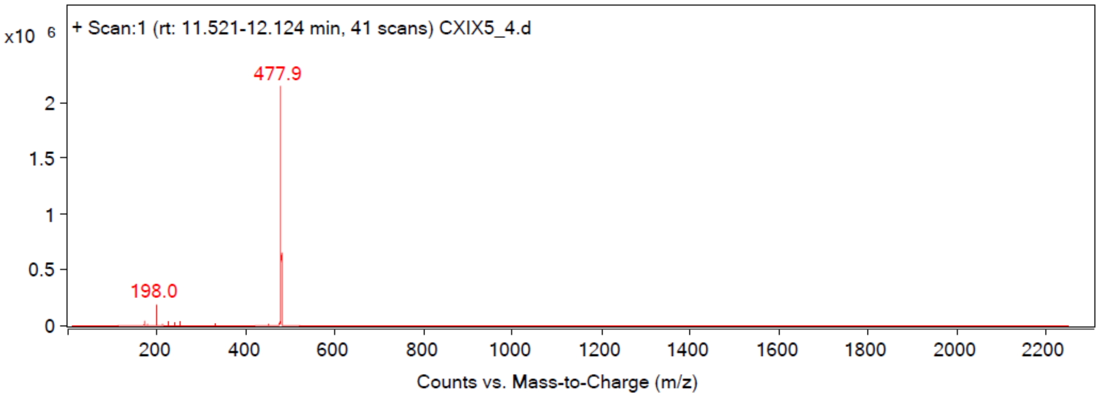

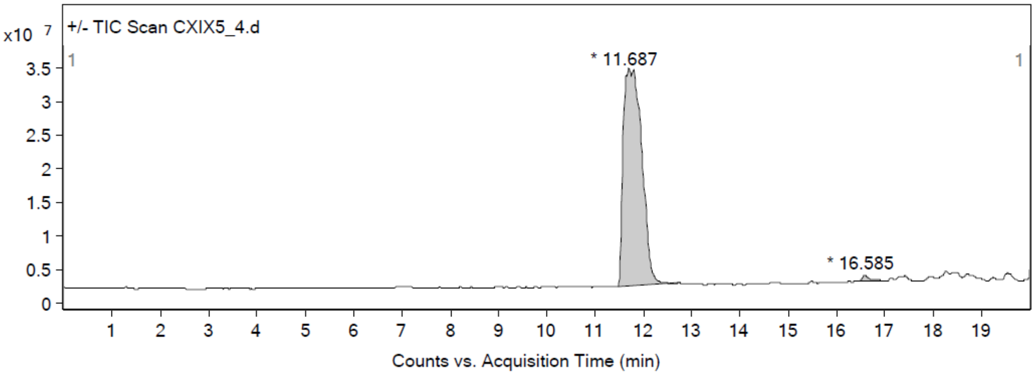

Integration Peak List

Peak	Start	RT	End	Height	Area	Area %
1	11.476	11.687	12.757	32331990.89	849627736.64	100
2	16.434	16.585	16.947	911139.02	10049820.27	1.18

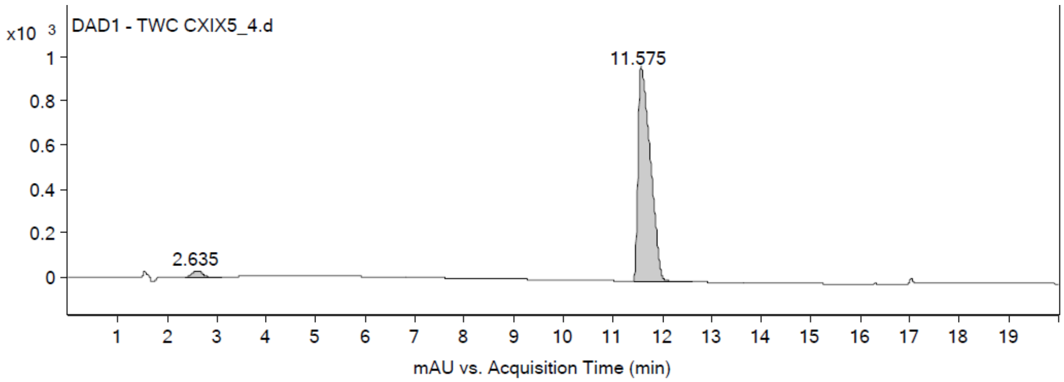

Integration Peak List

Peak	Start	RT	End	Height	Area	Area %
1	2.369	2.635	3.081	26.47	374.29	2.22
2	11.416	11.575	12.586	977.51	16897.38	100

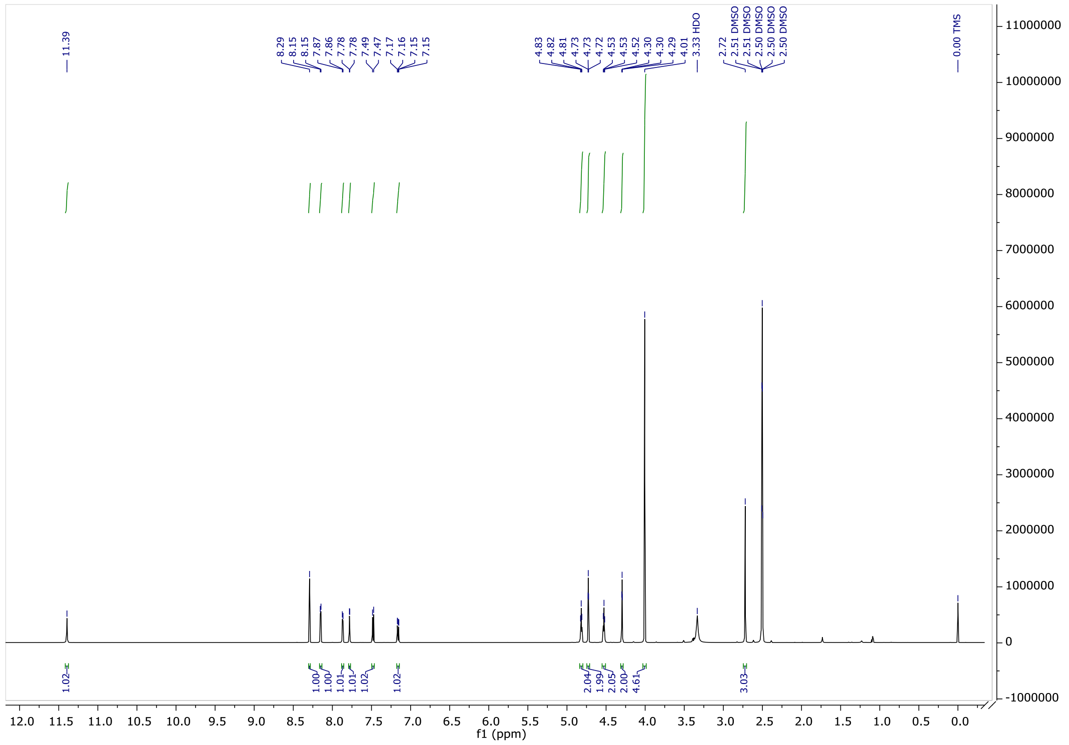

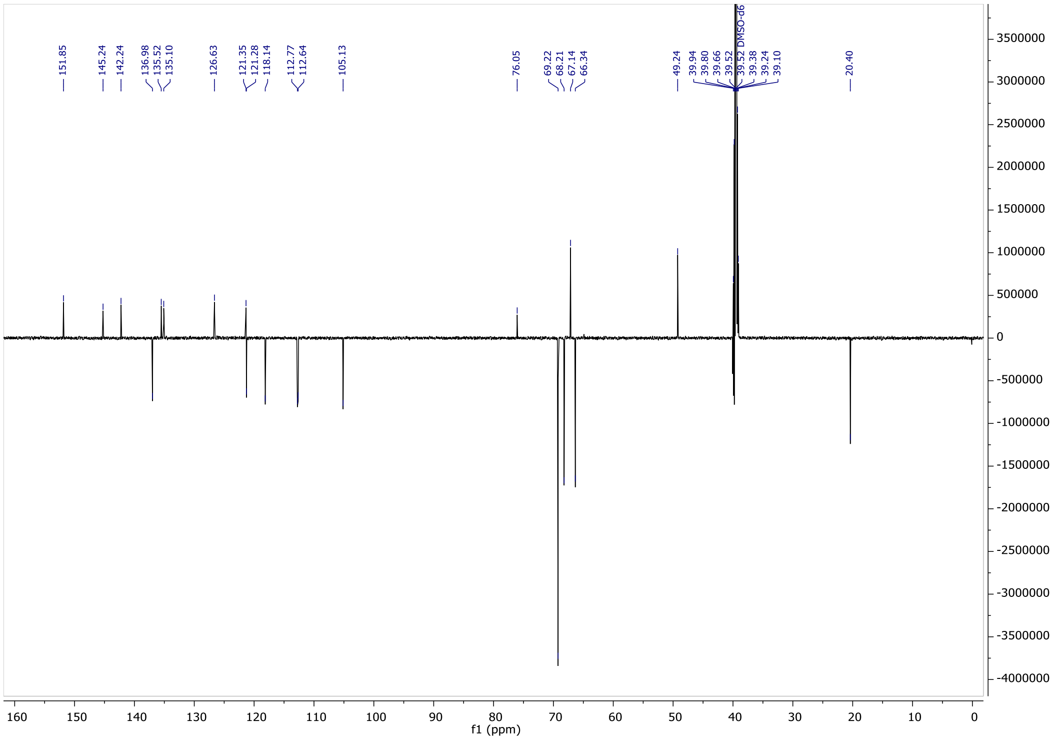
151.85
145.24
142.24
136.98
135.52
135.10
126.63
121.35
121.28
118.14
112.77
112.64
105.13
76.05
69.22
68.21
67.14
66.34
49.24
39.94
39.80
39.66
39.52
39.52 DMSO-d6
39.38
39.24
39.10
20.40
f1 (ppm)

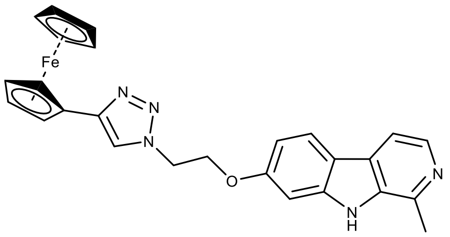
Fe
N
N
N
O
N
N
H

23

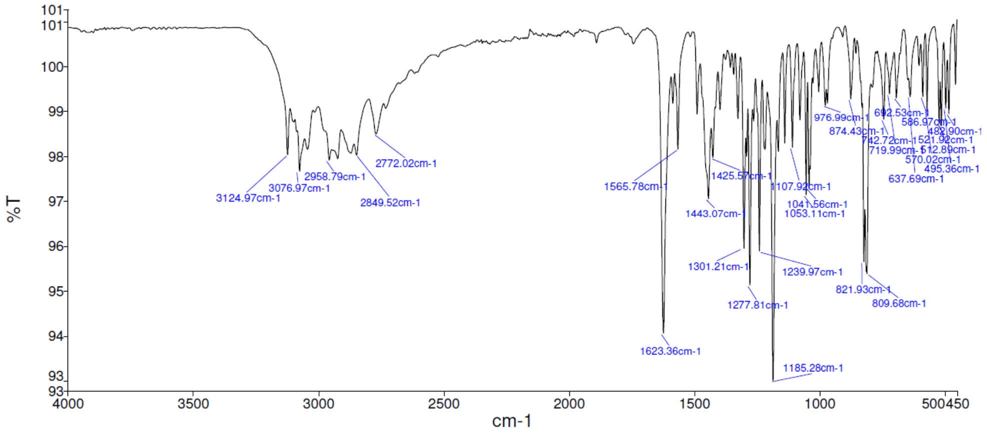
%T
cm-1
3124.97cm-1
3076.97cm-1
2958.79cm-1
2849.52cm-1
2772.02cm-1
1565.78cm-1
1623.36cm-1
1443.07cm-1
1425.57cm-1
1301.21cm-1
1277.81cm-1
1239.97cm-1
1185.28cm-1
1107.92cm-1
1041.56cm-1
1053.11cm-1
976.99cm-1
874.43cm-1
821.93cm-1
809.68cm-1
719.99cm-1
570.02cm-1
495.36cm-1
637.69cm-1

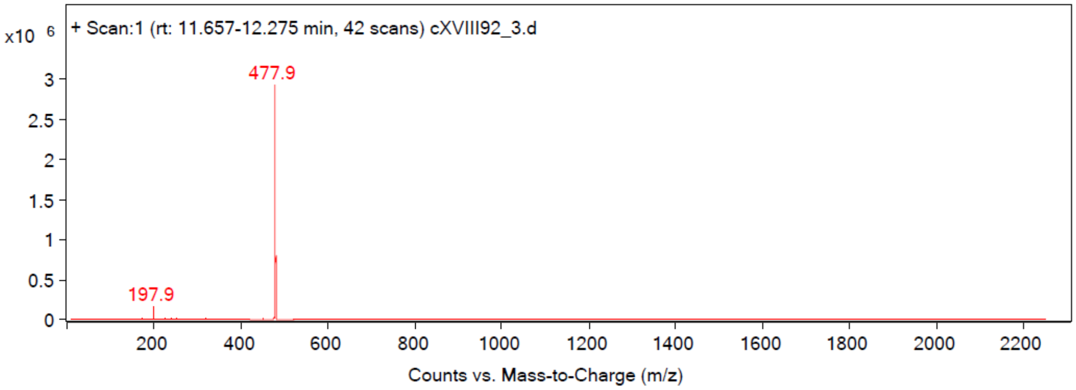

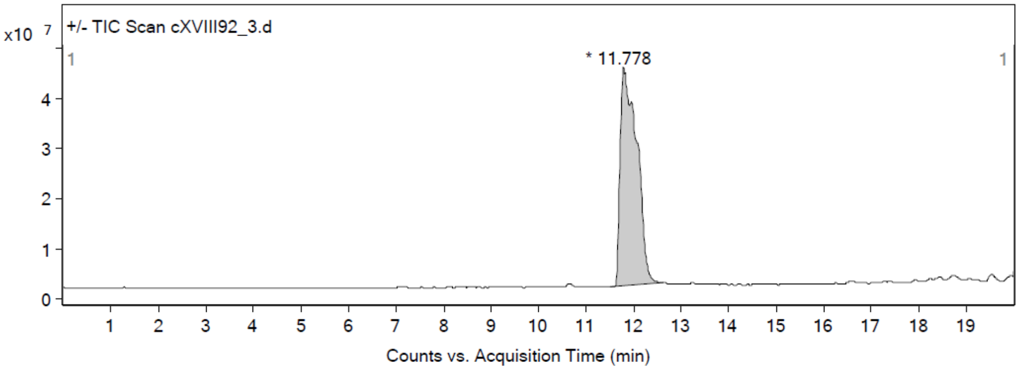

Integration Peak List

Peak	Start	RT	End	Height	Area	Area %
1	11.521	11.778	12.621	43527887.01	1069966983.92	100

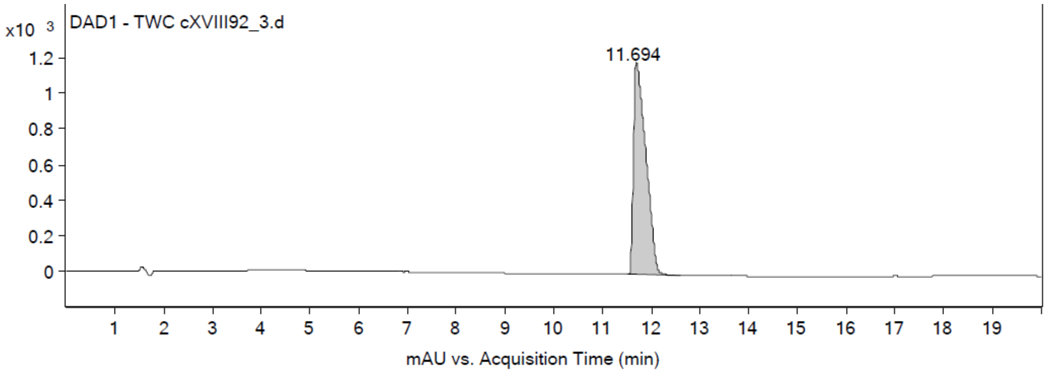

Integration Peak List

Peak	Start	RT	End	Height	Area	Area %
1	11.527	11.694	12.601	1194.83	21125.4	100

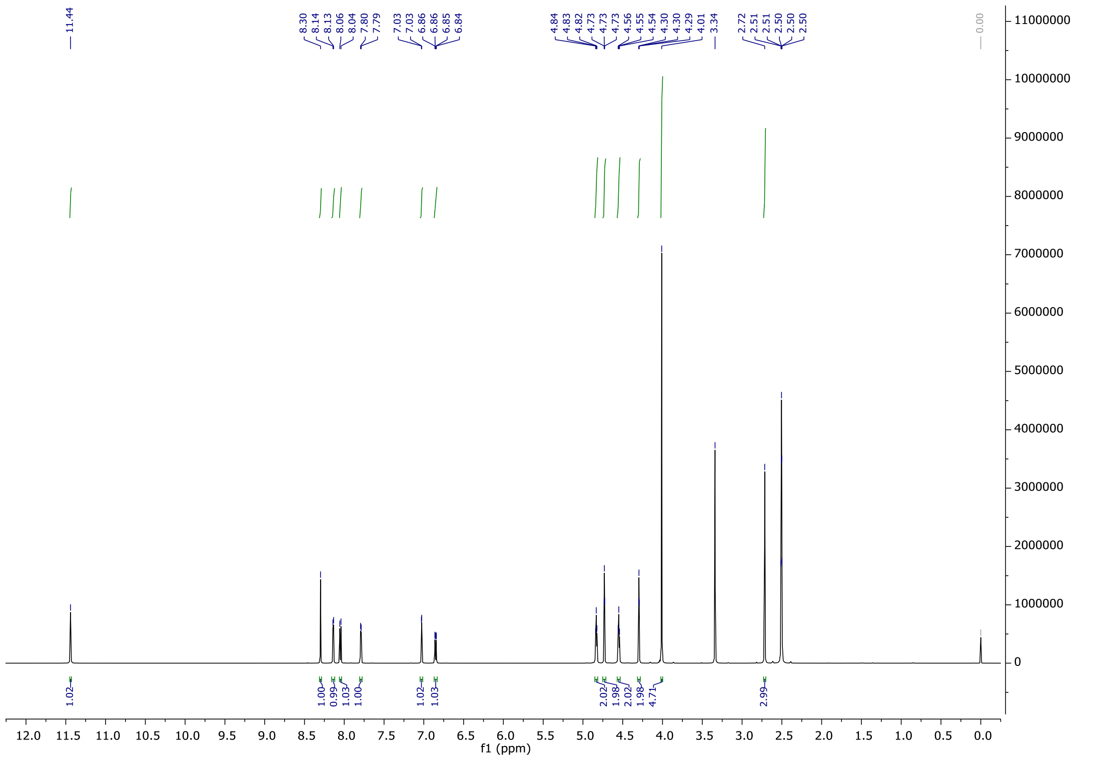

11.44
8.30
8.14
8.13
8.06
8.04
7.80
7.79
7.03
7.03
6.86
6.86
6.85
6.84
4.84
4.83
4.82
4.73
4.73
4.73
4.56
4.55
4.54
4.30
4.30
4.29
4.01
3.34
2.72
2.51
2.51
2.50
2.50
2.50
0.00
1.02
1.00
0.99
1.03
1.00
1.02
1.03
2.02
1.98
2.02
1.98
4.71
2.99
f1 (ppm)

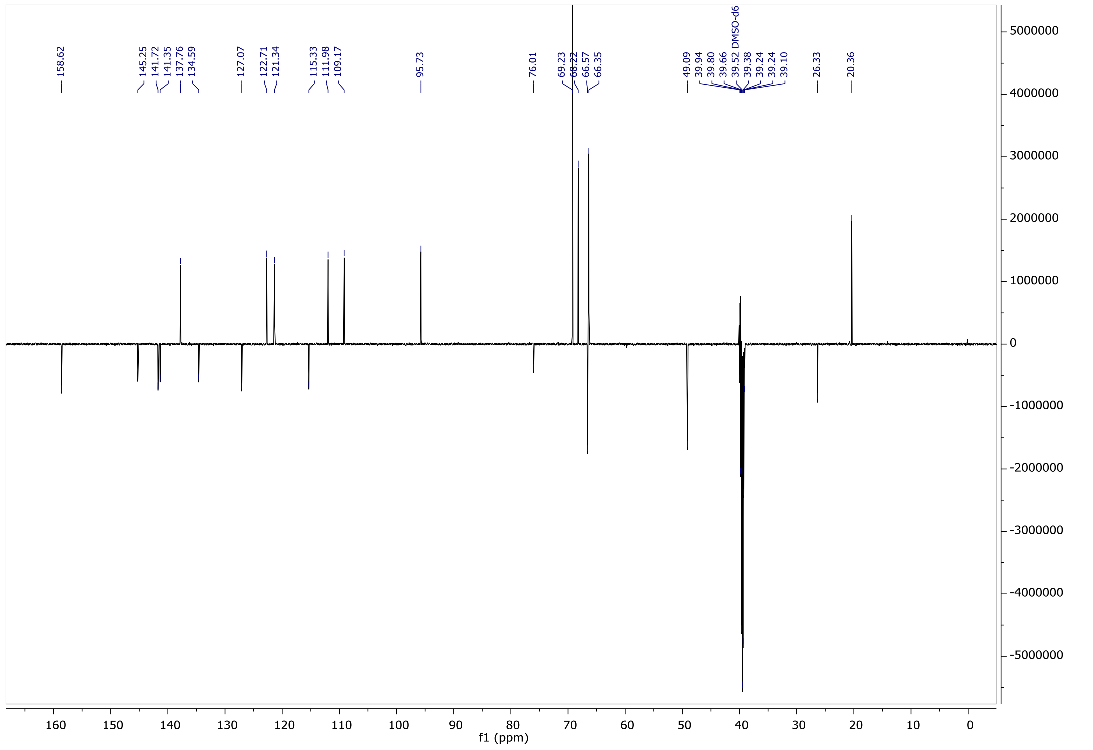

158.62
145.25
141.72
141.35
137.76
134.59
127.07
122.71
121.34
115.33
111.98
109.17
95.73
76.01
69.23
68.22
66.57
66.35
49.09
39.94
39.80
39.66
39.52 DMSO-d6
39.38
39.24
39.10
26.33
20.36
f1 (ppm)

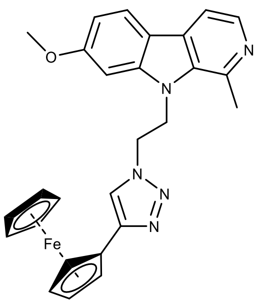

24

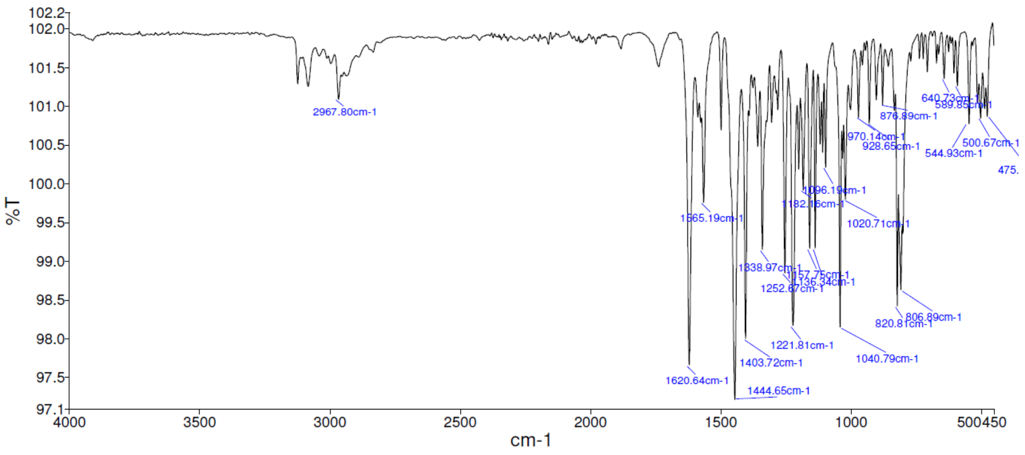

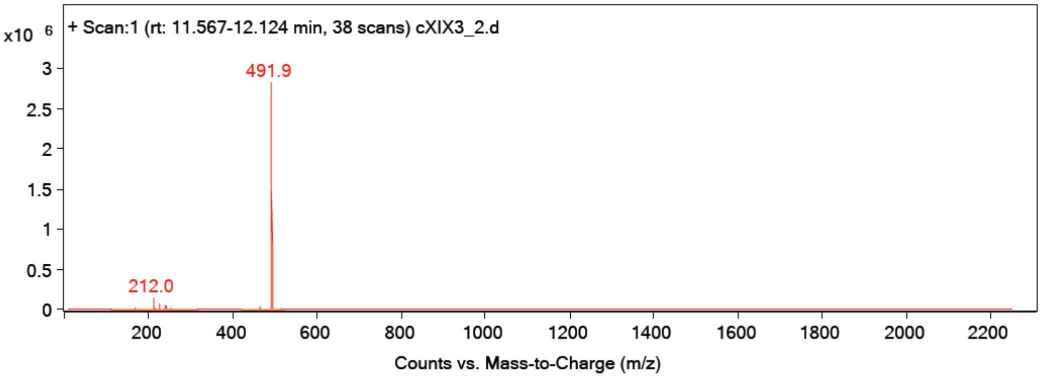

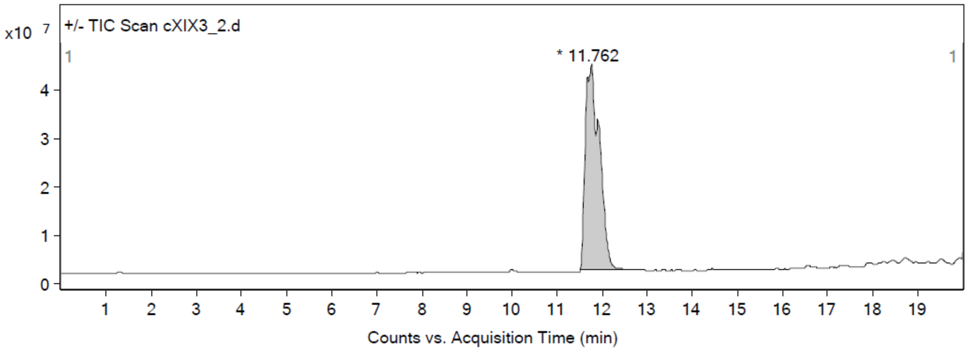

Integration Peak List

Peak	Start	RT	End	Height	Area	Area %
1	11.521	11.762	12.456	42407508.75	919756502.02	100

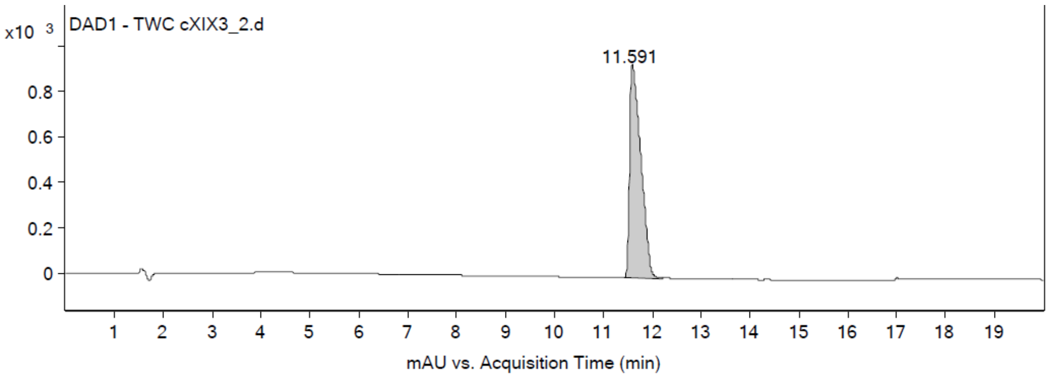

Integration Peak List

Peak	Start	RT	End	Height	Area	Area %
1	11.431	11.591	12.218	933.81	14867.52	100

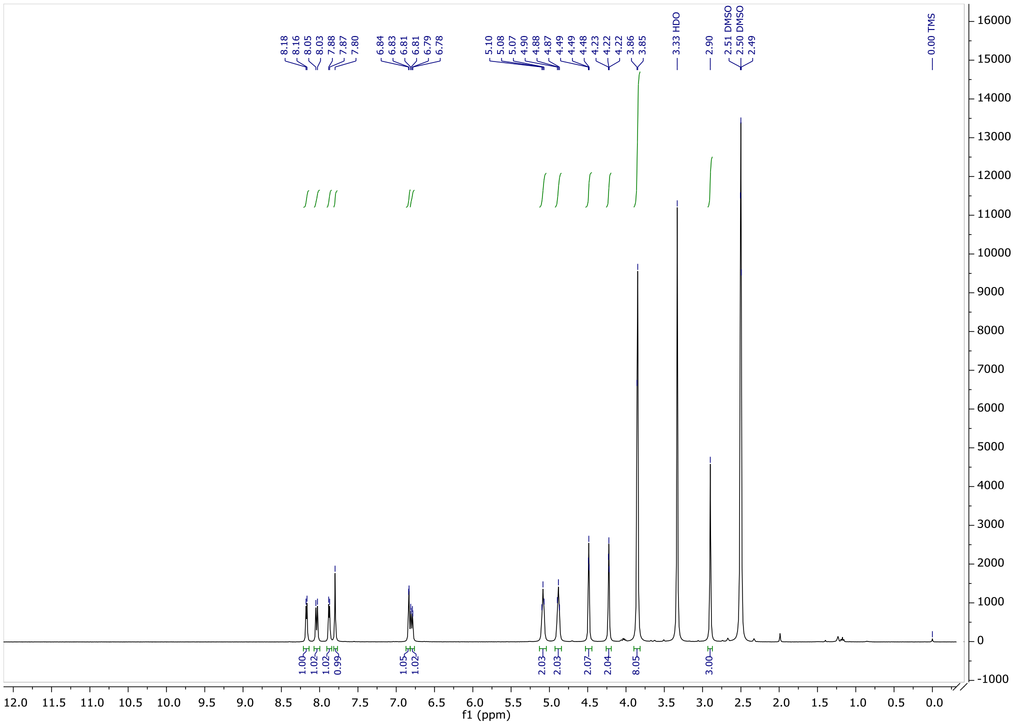

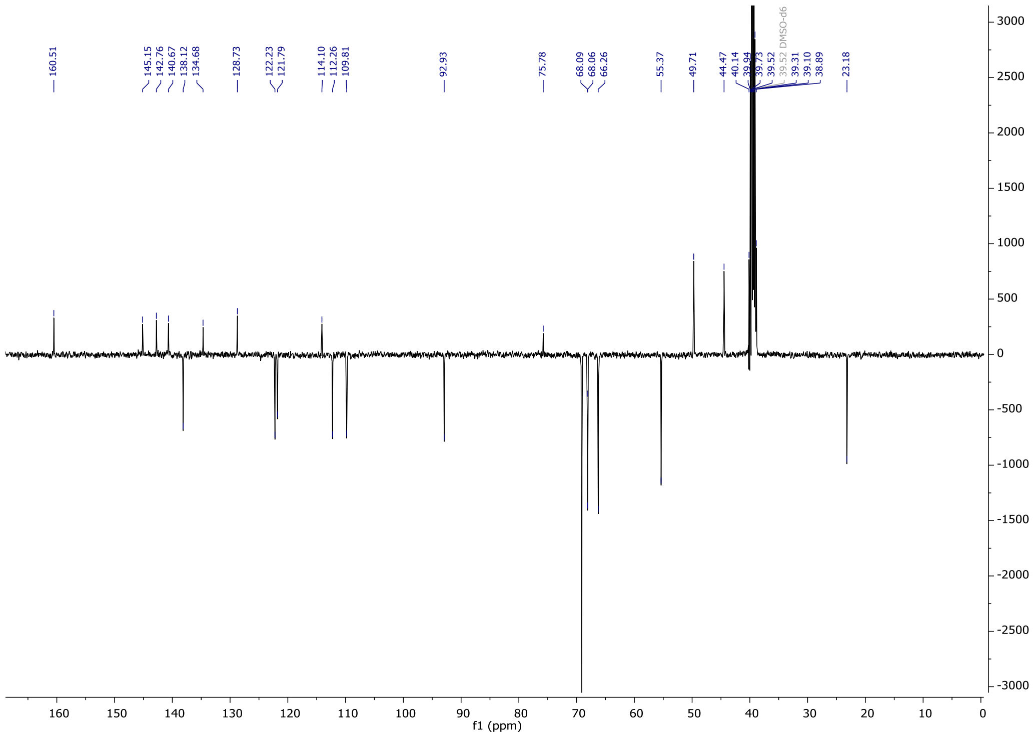
160.51
145.15
142.76
140.67
138.12
134.68
128.73
122.23
121.79
114.10
112.26
109.81
92.93
75.78
68.09
68.06
66.26
55.37
49.71
44.47
40.14
39.94
39.73
39.52
39.52 DMSO-d6
39.31
39.10
38.89
23.18
f1 (ppm)

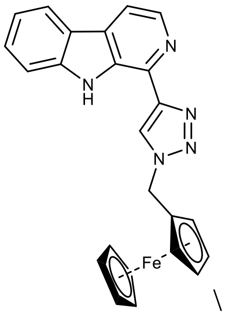

25

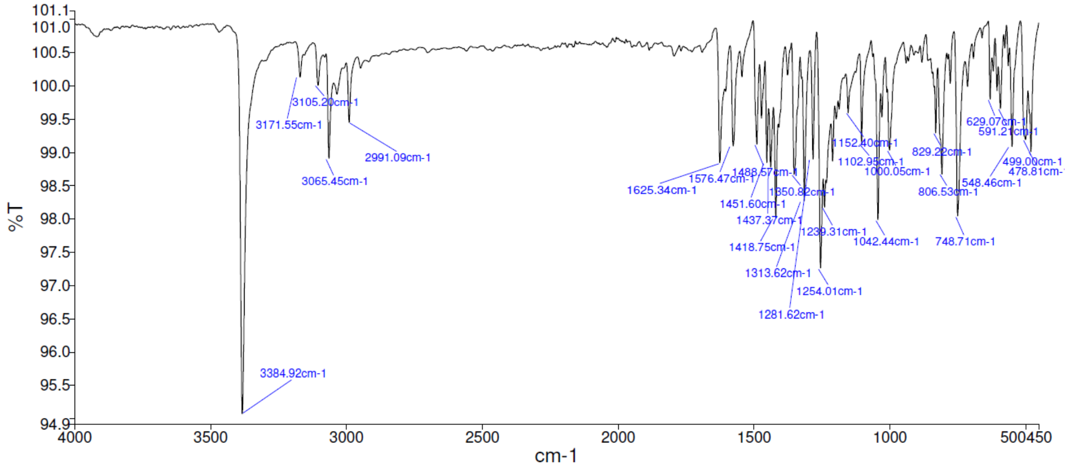
%T
cm-1
3384.92cm-1
3171.55cm-1
3105.20cm-1
3065.45cm-1
2991.09cm-1
1625.34cm-1
1576.47cm-1
1488.57cm-1
1451.60cm-1
1437.37cm-1
1418.75cm-1
1350.82cm-1
1313.62cm-1
1281.62cm-1
1254.01cm-1
1239.31cm-1
1152.40cm-1
1102.95cm-1
1042.44cm-1
1000.05cm-1
829.22cm-1
806.53cm-1
748.71cm-1
629.07cm-1
591.21cm-1
548.46cm-1
499.09cm-1
478.81cm-

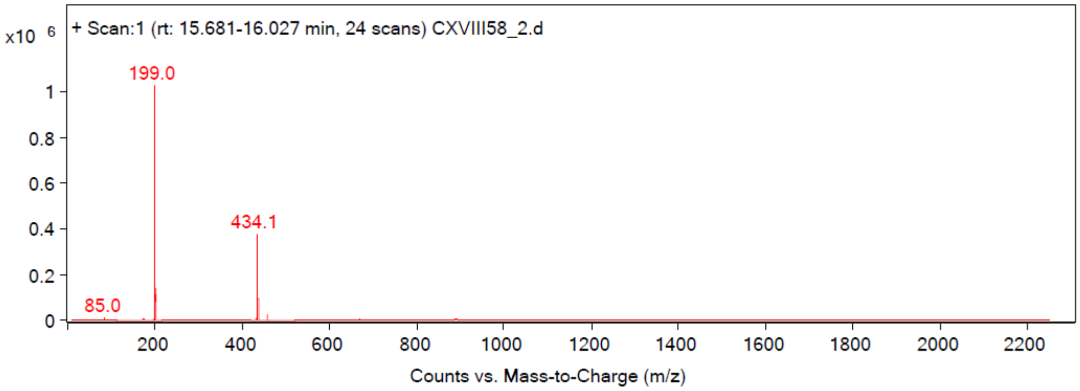

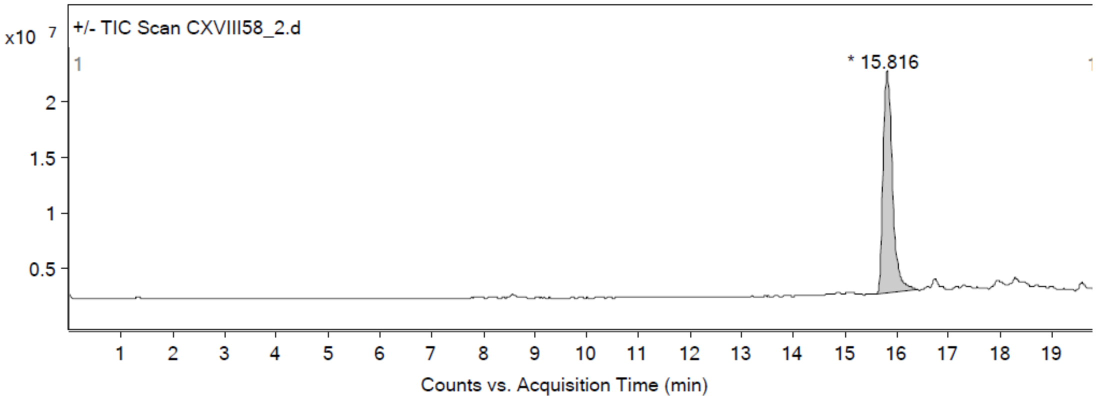

Integration Peak List

Peak	Start	RT	End	Height	Area	Area %
1	15.605	15.816	16.404	19997711.6	259614271.16	100

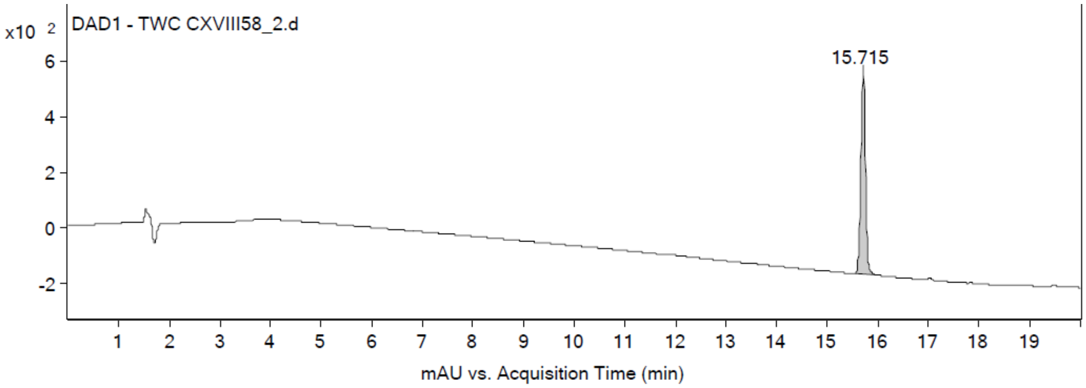

Integration Peak List

Peak	Start	RT	End	Height	Area	Area %
1	15.555	15.715	16.002	747.4	4724.58	100

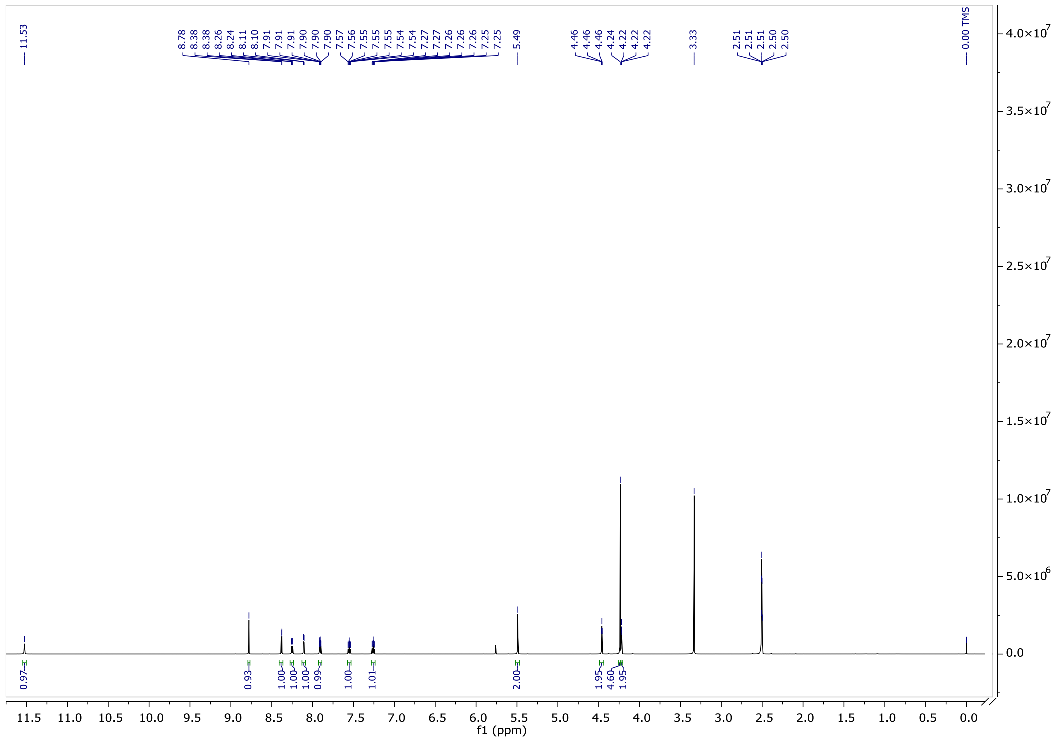
11.53
8.78 8.38 8.38 8.26 8.24 8.11 8.10 7.91 7.91 7.91 7.90 7.90 7.57 7.56 7.55 7.55 7.55 7.54 7.54 7.27 7.27 7.26 7.26 7.26 7.25 7.25
5.49
4.46 4.46 4.46 4.24 4.22 4.22 4.22
3.33
2.51 2.51 2.51 2.50 2.50
0.00 TMS
0.97 0.93 1.00 1.00 1.00 0.99 1.00 1.01 2.00 1.95 4.60 1.95
f1 (ppm)

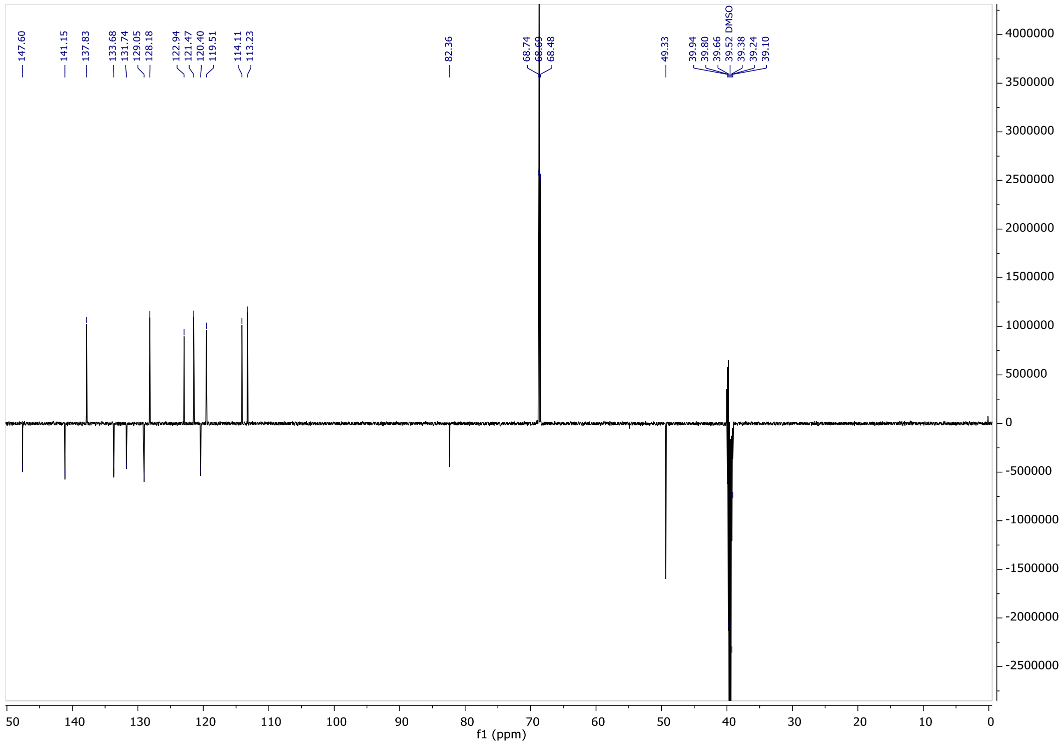
147.60 141.15 137.83 133.68 131.74 129.05 128.18 122.94 121.47 120.40 119.51 114.11 113.23
82.36
68.74 68.69 68.48
49.33
39.94 39.80 39.66 39.52 DMSO 39.38 39.24 39.10
f1 (ppm)

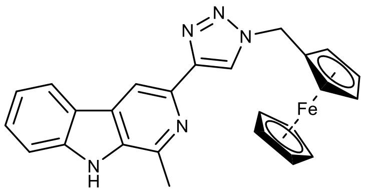

26

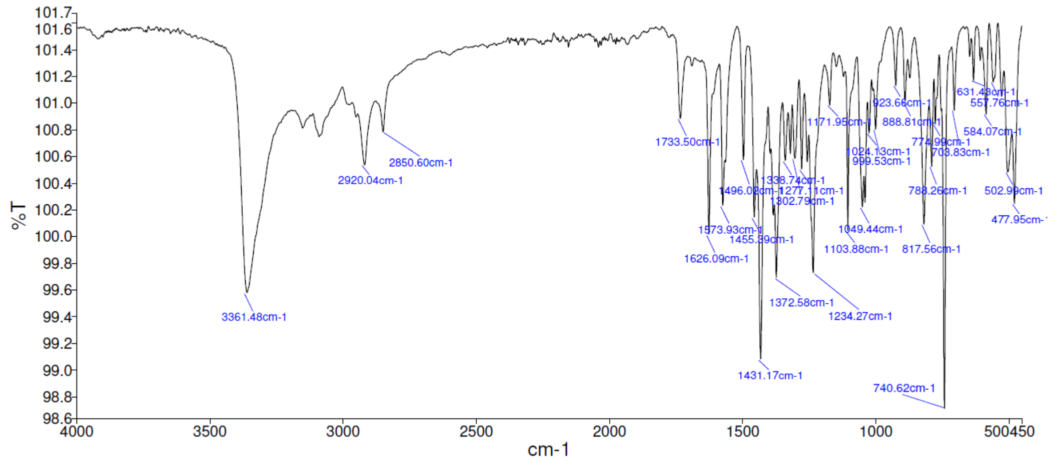

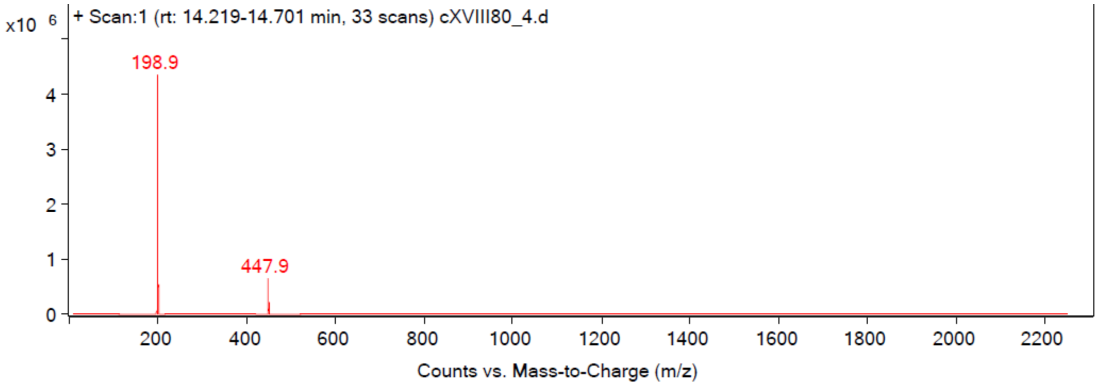

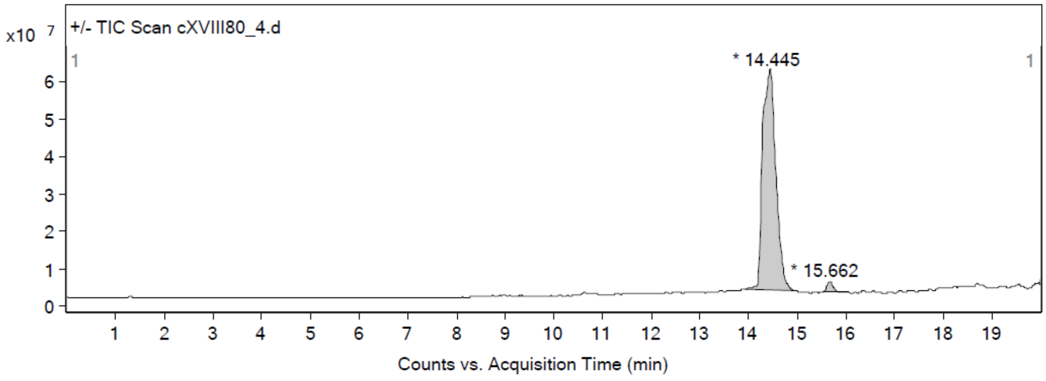

Integration Peak List

Peak	Start	RT	End	Height	Area	Area %
1	13.918	14.445	14.957	59020670.24	1134308297.78	100
2	15.545	15.662	16.057	2695116.31	25830575.15	2.28

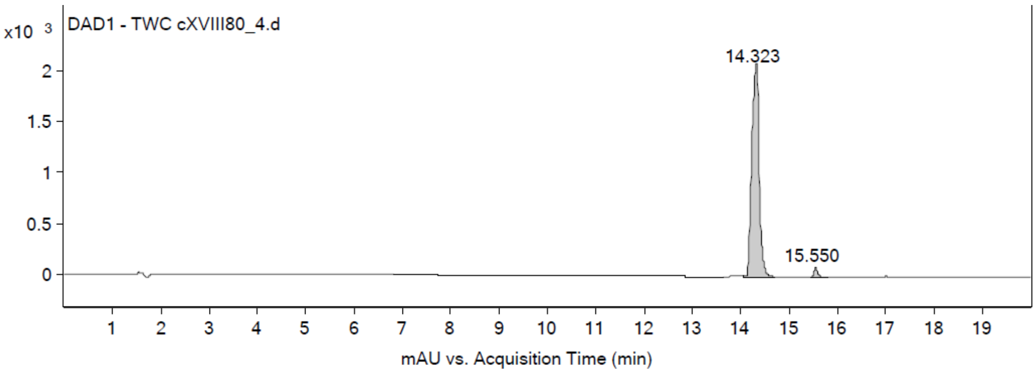

Integration Peak List

Peak	Start	RT	End	Height	Area	Area %
1	14.036	14.323	14.69	2083.3	22246.88	100
2	15.445	15.55	15.796	96.9	477.93	2.15

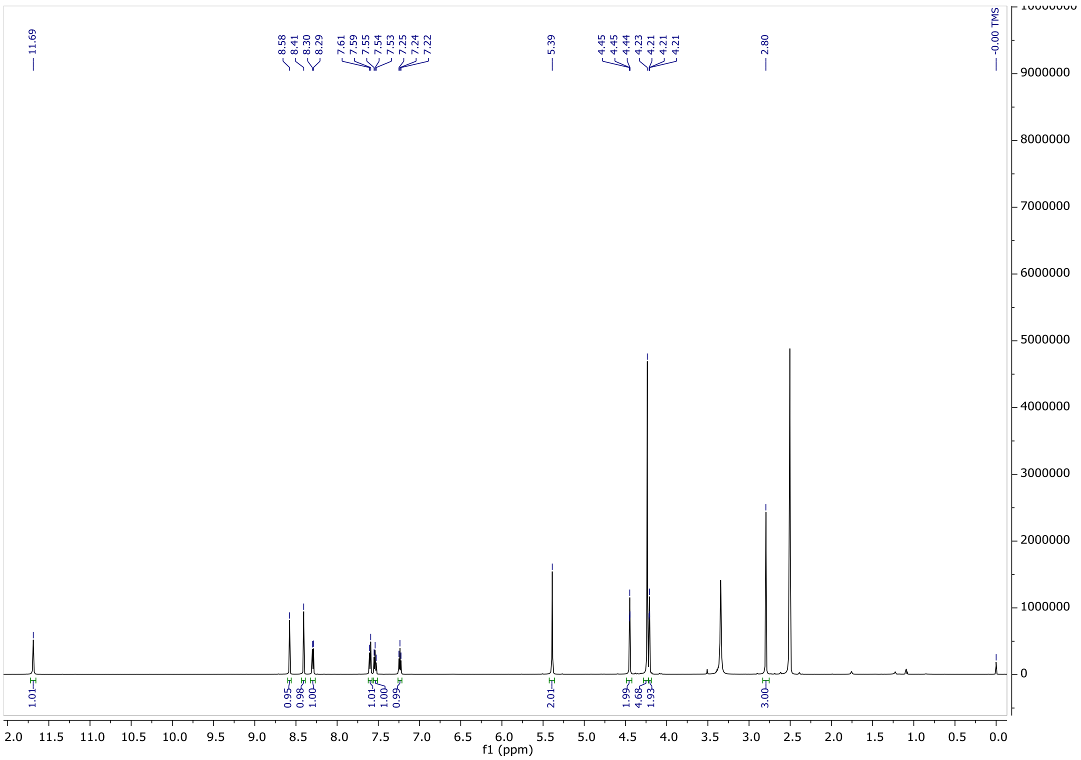

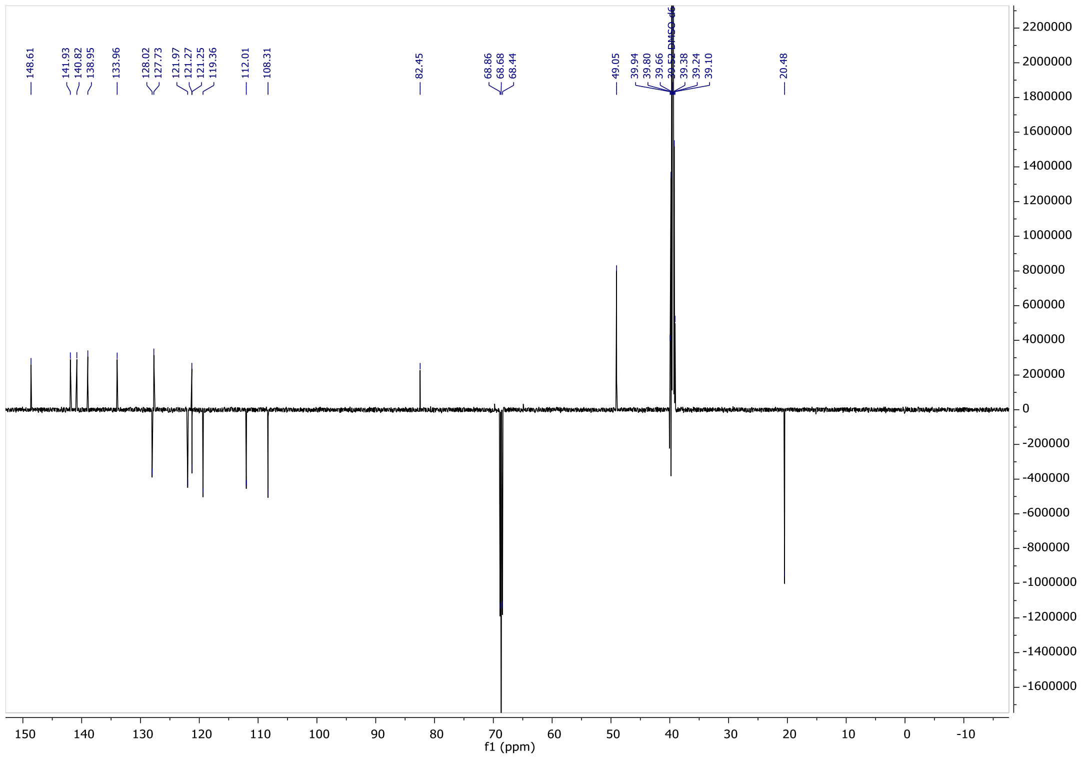
148.61
141.93
140.82
138.95
133.96
128.02
127.73
121.97
121.27
121.25
119.36
112.01
108.31
82.45
68.86
68.68
68.44
49.05
39.94
39.80
39.66
39.52 DMSO-d6
39.38
39.24
39.10
20.48
f1 (ppm)

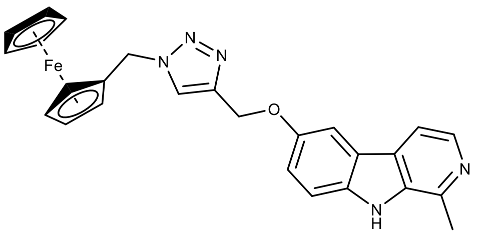

27

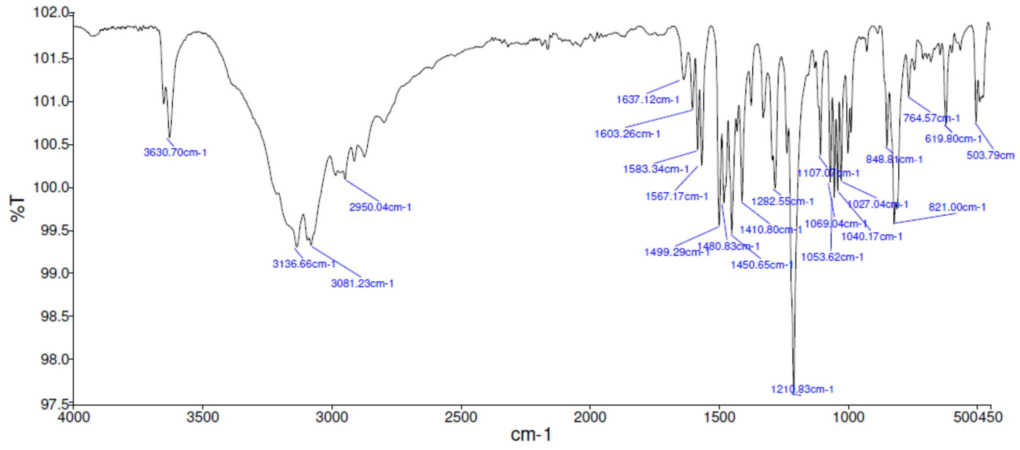
%T
cm-1
3630.70cm-1
3136.66cm-1
3081.23cm-1
2950.04cm-1
1637.12cm-1
1603.26cm-1
1583.34cm-1
1567.17cm-1
1499.29cm-1
1480.83cm-1
1450.65cm-1
1410.80cm-1
1282.55cm-1
1210.83cm-1
1107.07cm-1
1069.04cm-1
1053.62cm-1
1040.17cm-1
1027.04cm-1
848.81cm-1
821.00cm-1
764.57cm-1
619.80cm-1
503.79cm

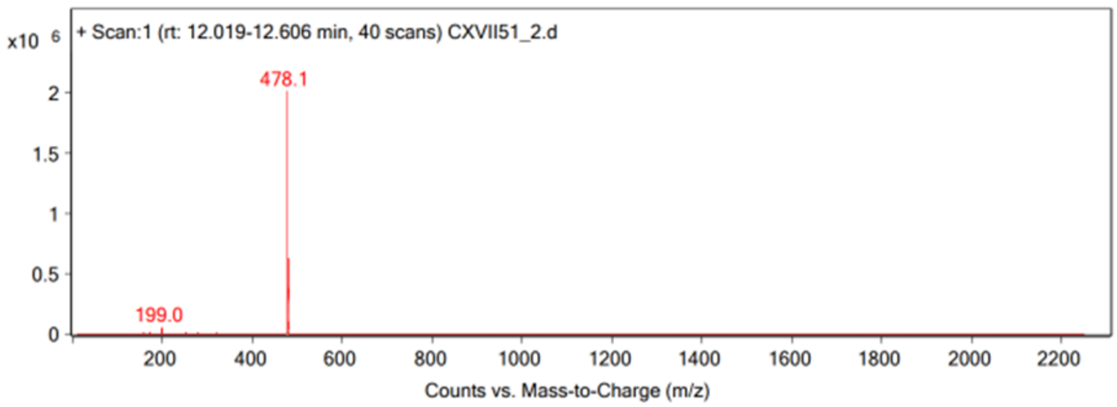

x10 6
+ Scan:1 (rt: 12.019-12.606 min, 40 scans) CXVII51_2.d
478.1
199.0
Counts vs. Mass-to-Charge (m/z)

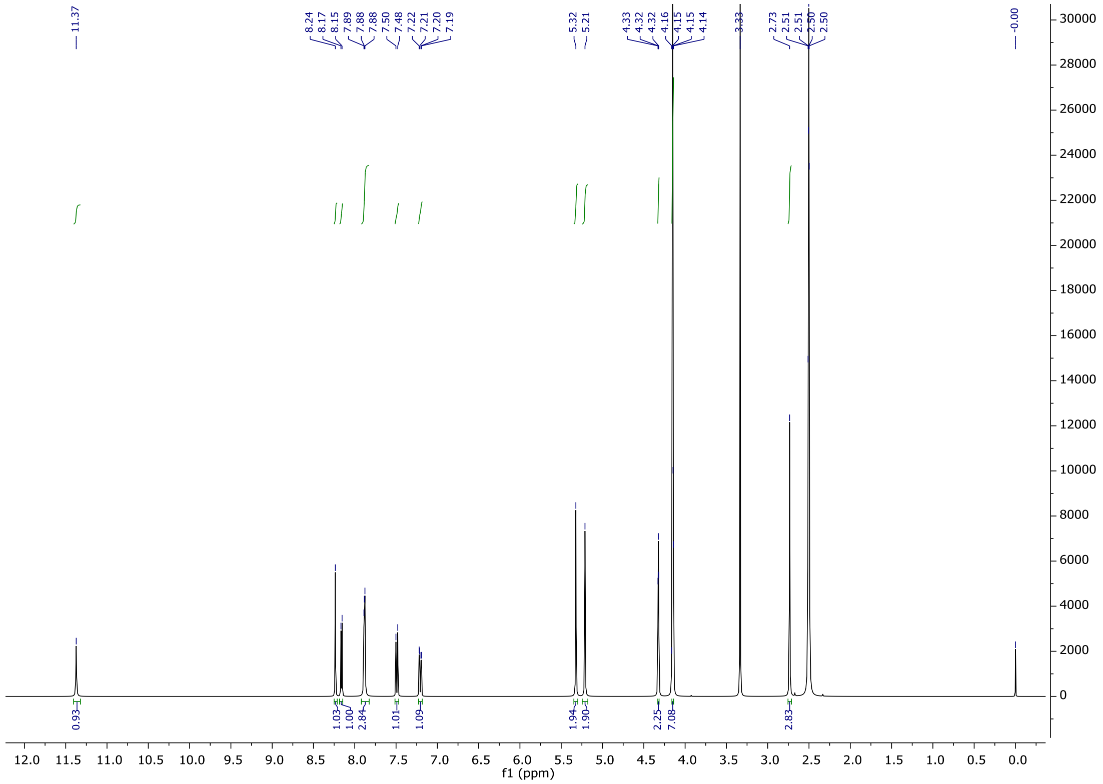

11.37
8.24 8.17 8.15 7.89 7.88 7.88 7.50 7.48 7.22 7.21 7.20 7.19
5.32 5.21
4.33 4.32 4.32 4.16 4.15 4.15 4.14
3.33
2.73 2.51 2.51 2.50 2.50
-0.00
0.93 1.03 1.00 2.84 1.01 1.09 1.94 1.90 2.25 7.08 2.83
f1 (ppm)

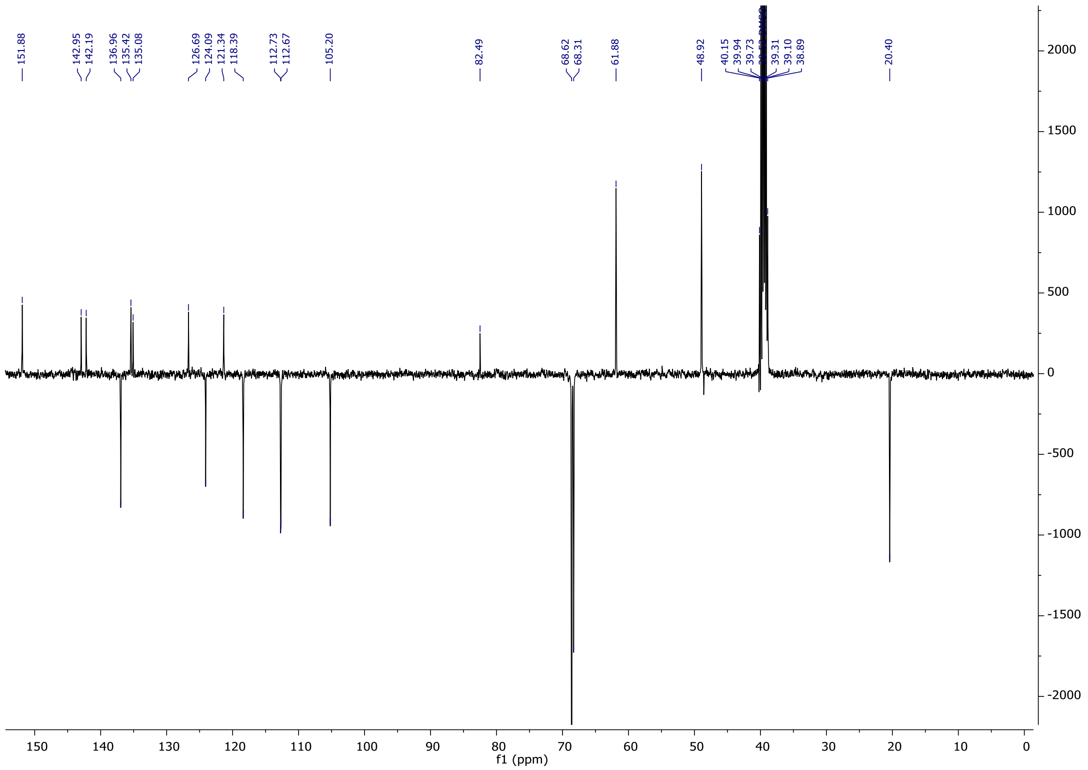

151.88
142.95
142.19
136.96
135.42
135.08
126.69
124.09
121.34
118.39
112.73
112.67
105.20
82.49
68.62
68.31
61.88
48.92
40.15
39.94
39.73
39.31
39.10
38.89
20.40
2000
1500
1000
500
0
-500
-1000
-1500
-2000
150
140
130
120
110
100
90
80
70
60
50
40
30
20
10
0
f1 (ppm)

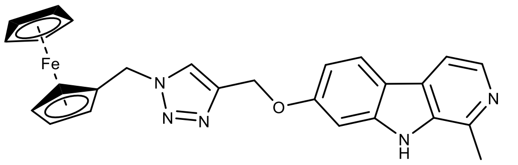

Fe
N
N
N
O
N
N
H

28

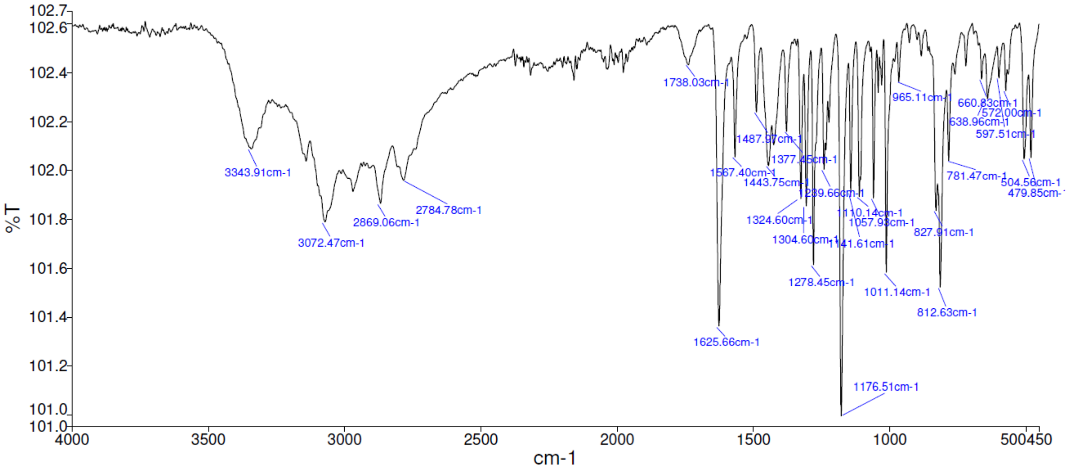

102.7
102.6
102.4
102.2
102.0
101.8
101.6
101.4
101.2
101.0
101.0
%T
4000
3500
3000
2500
2000
1500
1000
500450
cm-1
3343.91cm-1
3072.47cm-1
2869.06cm-1
2784.78cm-1
1738.03cm-1
1625.66cm-1
1487.97cm-1
1443.75cm-1
1377.45cm-1
1324.60cm-1
1304.60cm-1
1278.45cm-1
1239.66cm-1
1176.51cm-1
1141.61cm-1
1110.14cm-1
1057.93cm-1
1011.14cm-1
965.11cm-1
827.91cm-1
812.63cm-1
781.47cm-1
660.83cm-1
638.96cm-1
597.51cm-1
572.00cm-1
504.56cm-1

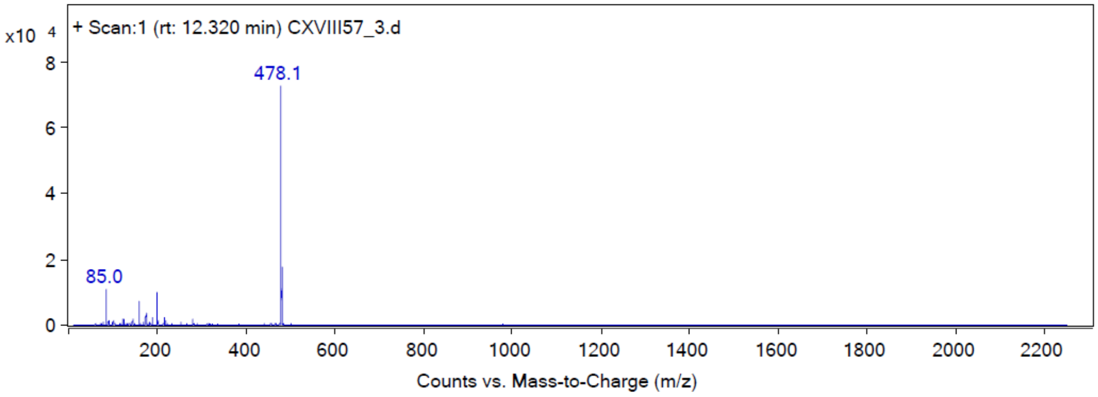

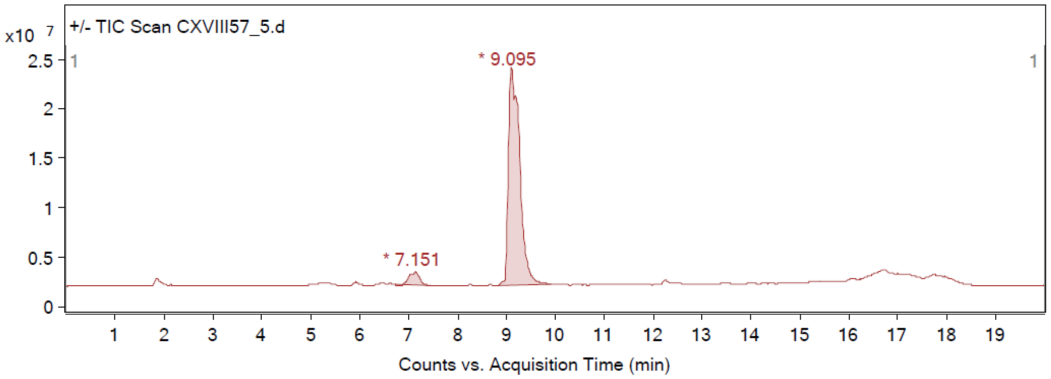

Integration Peak List

Peak	Start	RT	End	Height	Area	Area %
1	6.729	7.151	7.483	1334525.72	19487940.07	5.34
2	8.794	9.095	9.909	21941635.24	364759531.81	100

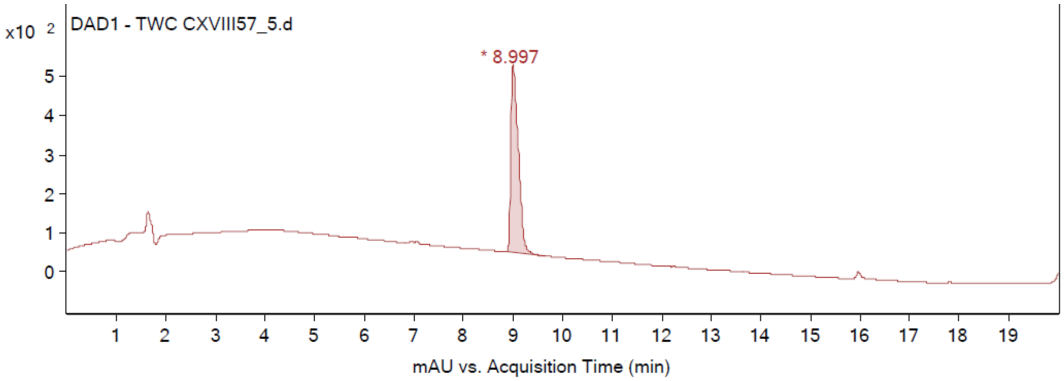

Integration Peak List

Peak	Start	RT	End	Height	Area	Area %
1	8.864	8.997	9.644	479.79	5004.85	100

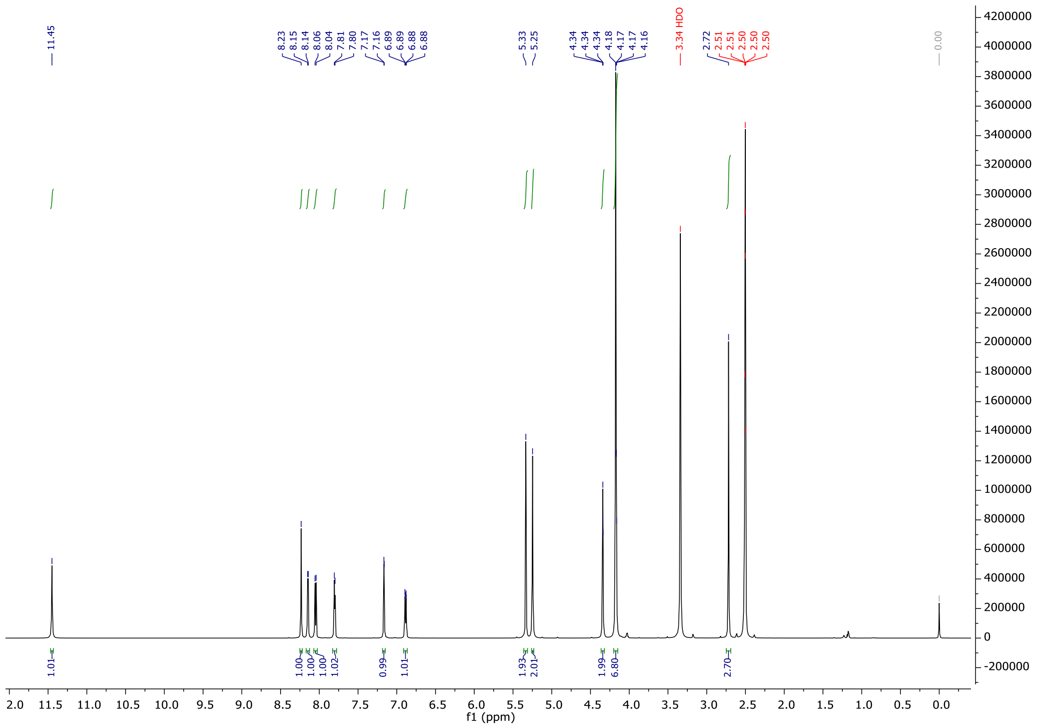
11.45
8.23 8.15 8.14 8.06 8.04 7.81 7.80 7.17 7.16 6.89 6.89 6.88 6.88
5.33 5.25
4.34 4.34 4.34 4.18 4.17 4.17 4.16
3.34 HDO
2.72 2.51 2.51 2.50 2.50 2.50
0.00
1.01
1.00 1.00 1.00 1.02
0.99 1.01
1.93 2.01
1.99 6.80
2.70
f1 (ppm)

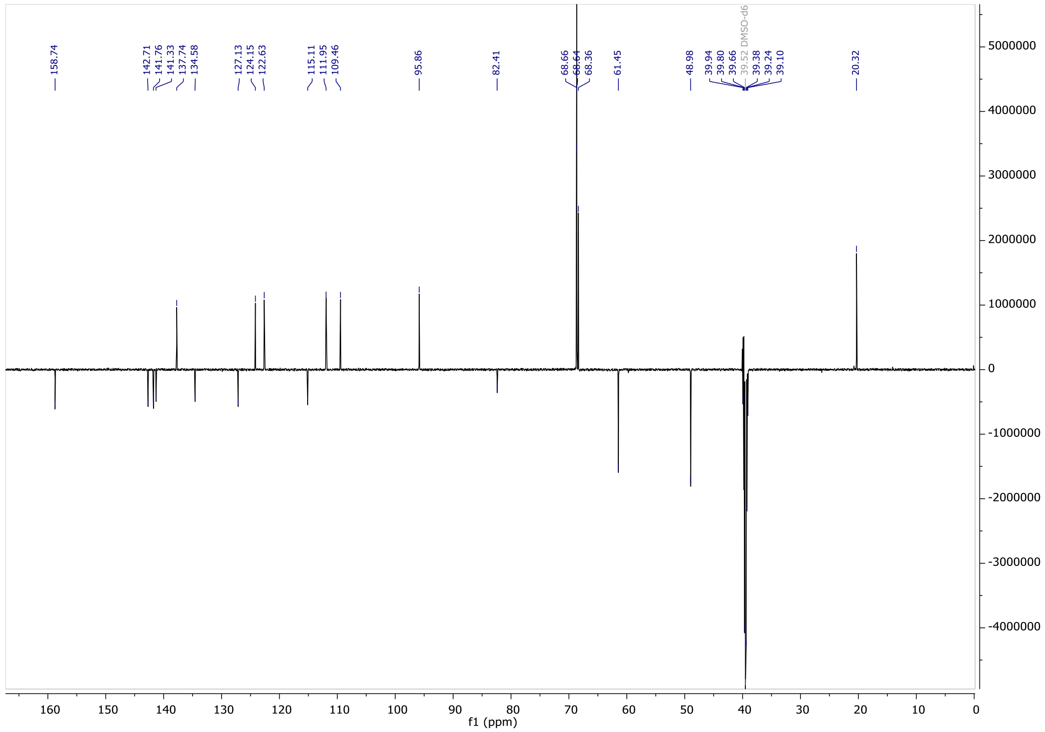
158.74
142.71 141.76 141.33 137.74 134.58
127.13 124.15 122.63
115.11 111.95 109.46
95.86
82.41
68.66 68.64 68.36
61.45
48.98
39.94 39.80 39.66 39.52 DMSO-d6 39.38 39.24 39.10
20.32
f1 (ppm)

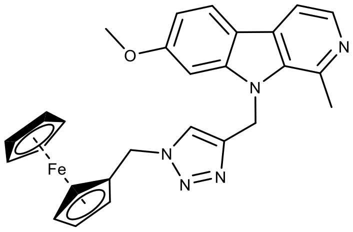

29

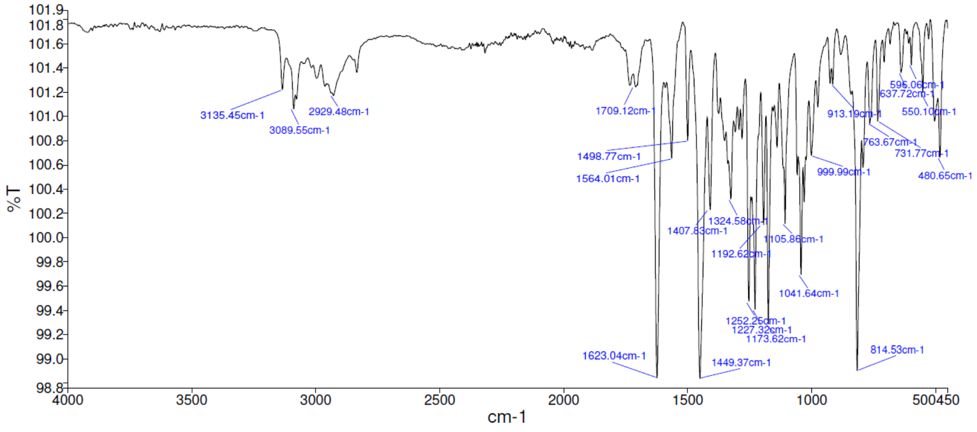

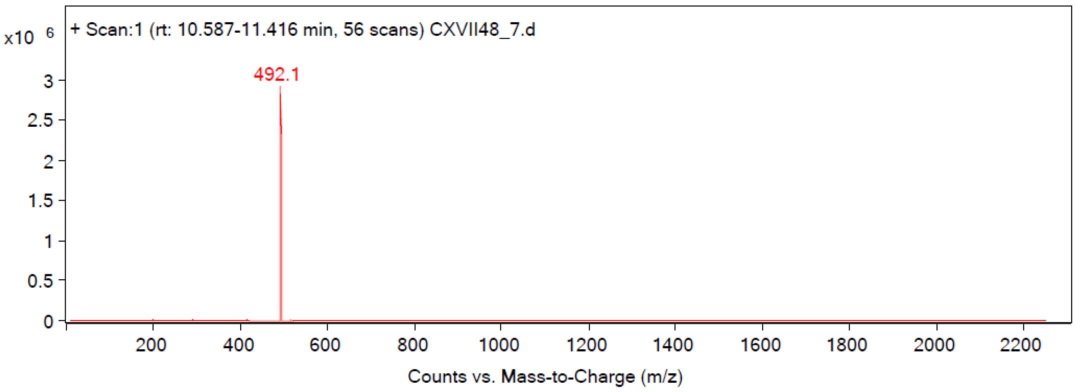

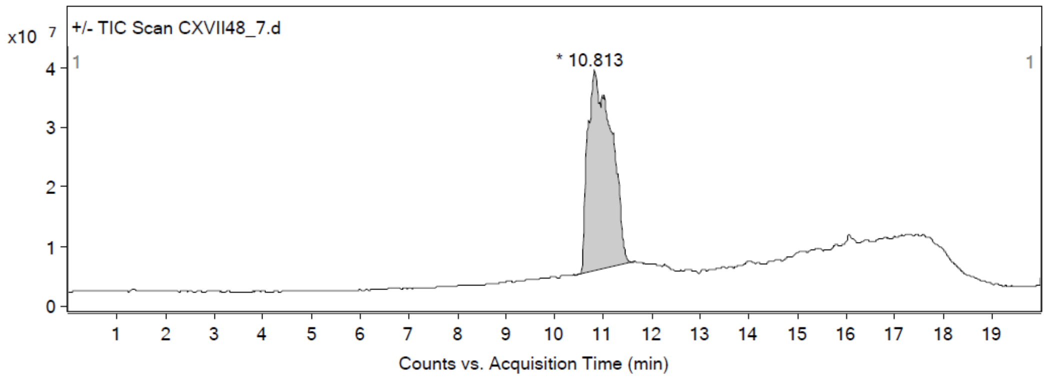

Integration Peak List

Peak	Start	RT	End	Height	Area	Area %
1	10.406	10.813	11.642	33720931.72	1130409678.26	100

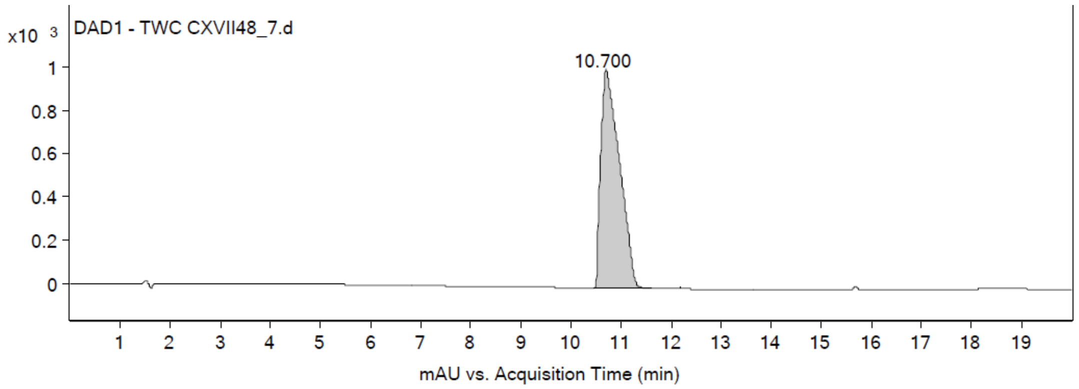

Integration Peak List

Peak	Start	RT	End	Height	Area	Area %
1	10.466	10.7	11.606	1011.94	26136.72	100

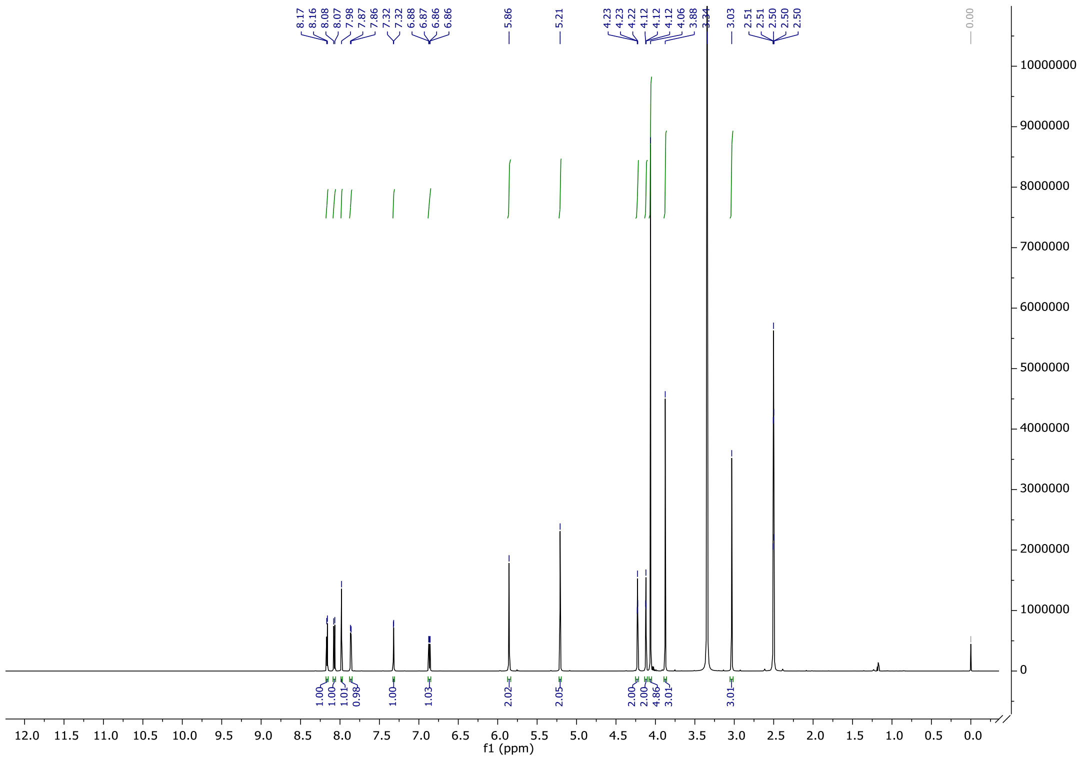

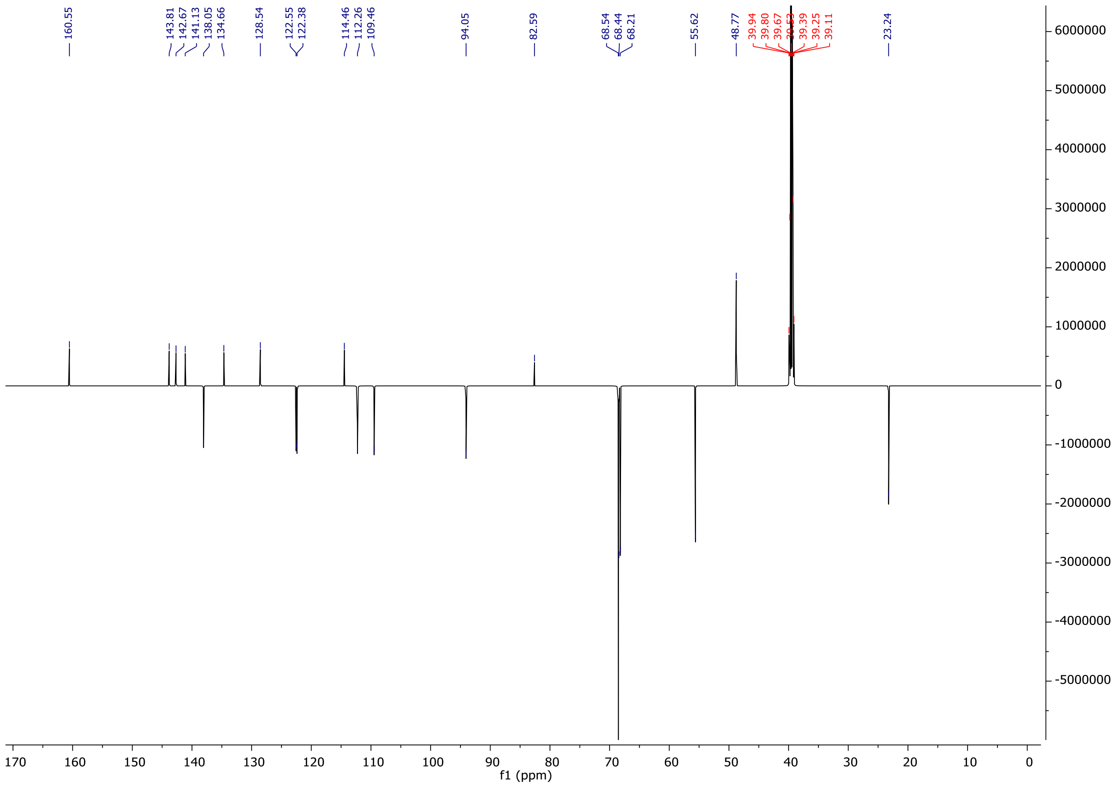
160.55
143.81
142.67
141.13
138.05
134.66
128.54
122.55
122.38
114.46
112.26
109.46
94.05
82.59
68.54
68.44
68.21
55.62
48.77
39.94
39.80
39.67
39.39
39.25
39.11
23.24
6000000
5000000
4000000
3000000
2000000
1000000
0
-1000000
-2000000
-3000000
-4000000
-5000000
170 160 150 140 130 120 110 100 90 80 70 60 50 40 30 20 10 0
f1 (ppm)

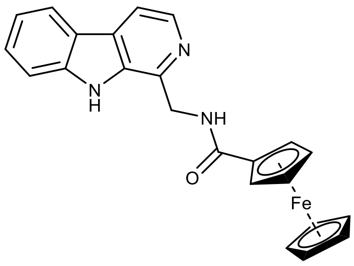
N
H
NH
O
Fe

30

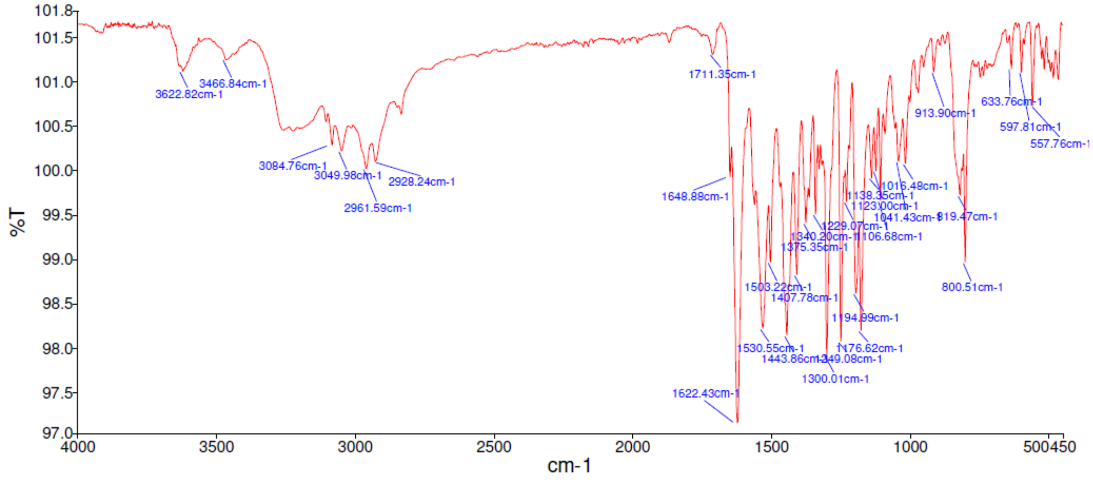
%T
cm-1
4000 3500 3000 2500 2000 1500 1000 500450
3622.82cm-1
3466.84cm-1
3084.76cm-1
3049.98cm-1
2961.59cm-1
2928.24cm-1
1711.35cm-1
1648.88cm-1
1622.43cm-1
1530.55cm-1
1503.22cm-1
1407.78cm-1
1300.01cm-1
1194.99cm-1
1176.62cm-1
913.90cm-1
800.51cm-1
633.76cm-1
597.81cm-1

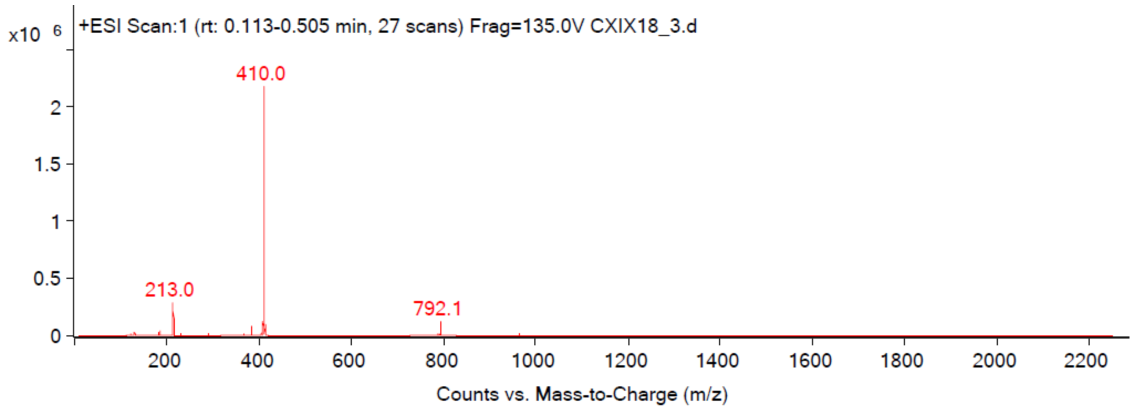
+ESI Scan:1 (rt: 0.113-0.505 min, 27 scans) Frag=135.0V CXIX18_3.d
410.0
213.0
792.1
Counts vs. Mass-to-Charge (m/z)

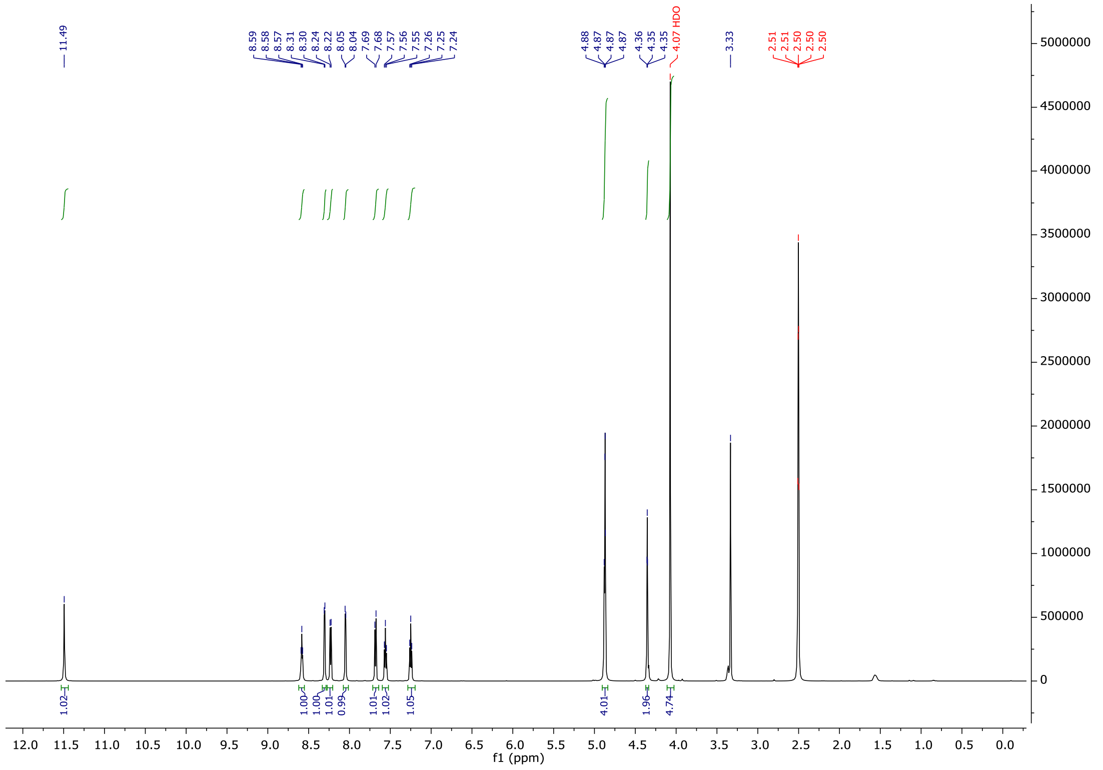
11.49
8.59
8.58
8.57
8.31
8.30
8.24
8.22
8.05
8.04
7.69
7.68
7.57
7.56
7.55
7.26
7.25
7.24
4.88
4.87
4.87
4.87
4.36
4.35
4.35
4.07 HDO
3.33
2.51
2.51
2.50
2.50
2.50
f1 (ppm)

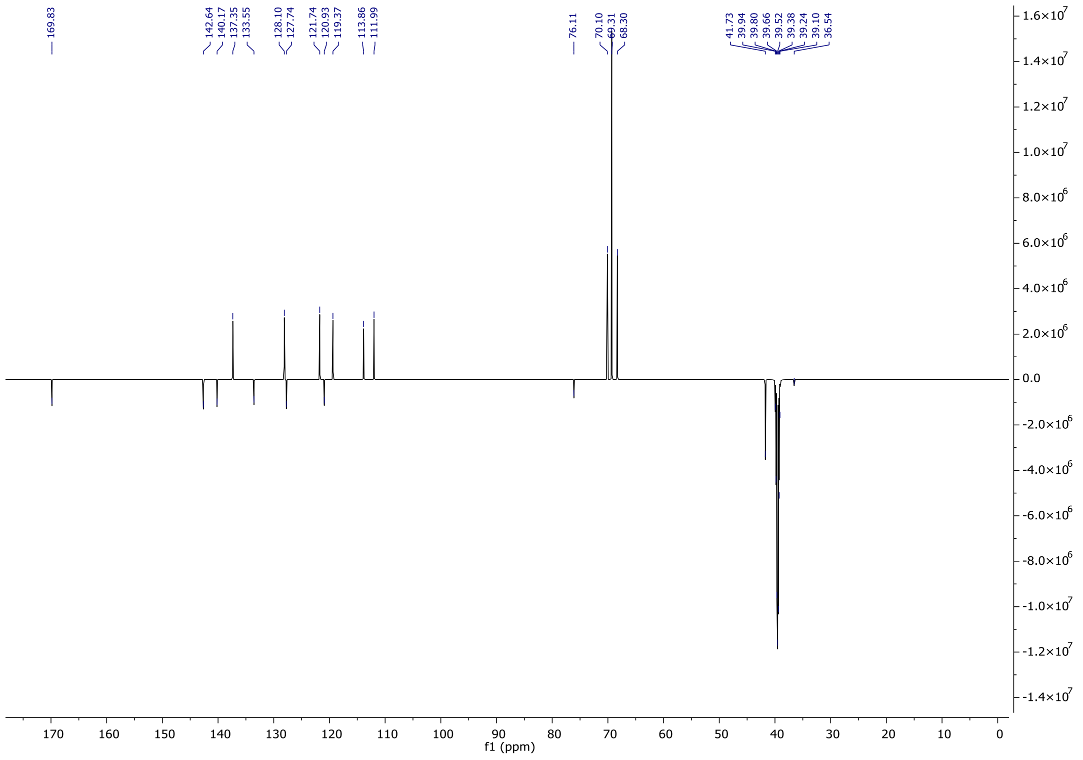
169.83
142.64
140.17
137.35
133.55
128.10
127.74
121.74
120.93
119.37
113.86
111.99
76.11
70.10
69.31
68.30
41.73
39.94
39.80
39.66
39.52
39.38
39.24
39.10
36.54
f1 (ppm)

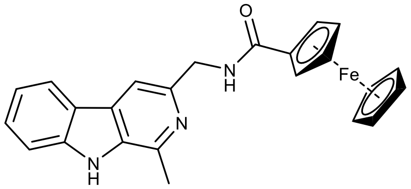
O
N
H
Fe
N
N
H

31

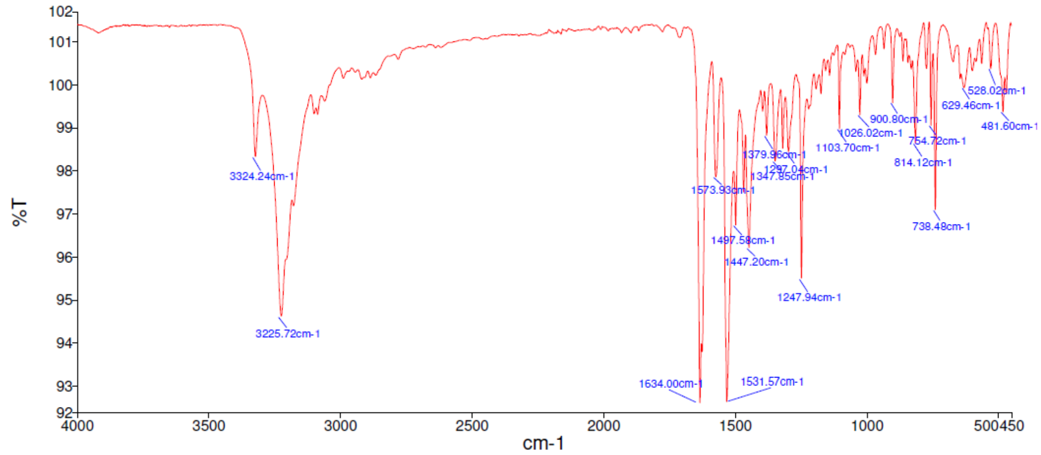
%T
cm-1
3324.24cm-1
3225.72cm-1
1634.00cm-1
1531.57cm-1
1573.93cm-1
1497.58cm-1
1447.20cm-1
1379.96cm-1
1347.85cm-1
1297.04cm-1
1247.94cm-1
1103.70cm-1
1026.02cm-1
900.80cm-1
814.12cm-1
754.72cm-1
738.48cm-1
629.46cm-1
528.02cm-1
481.60cm-1

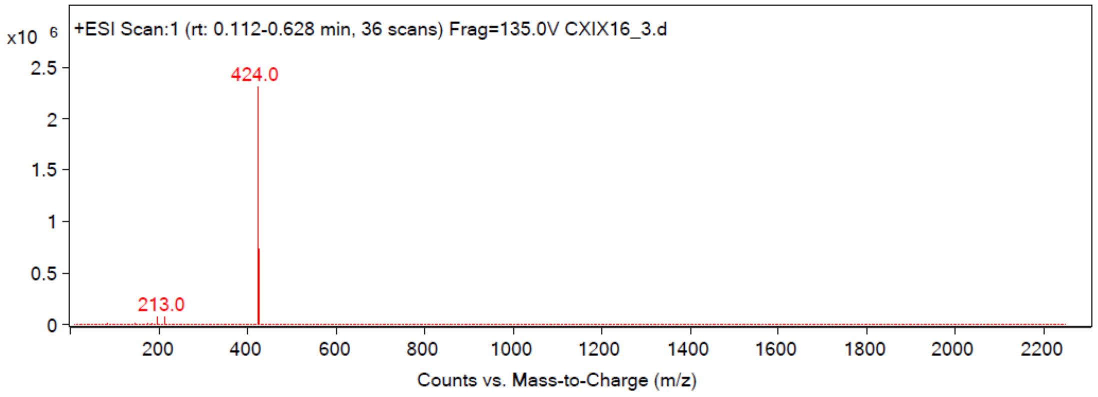
+ESI Scan:1 (rt: 0.112-0.628 min, 36 scans) Frag=135.0V CXIX16_3.d
424.0
213.0
Counts vs. Mass-to-Charge (m/z)

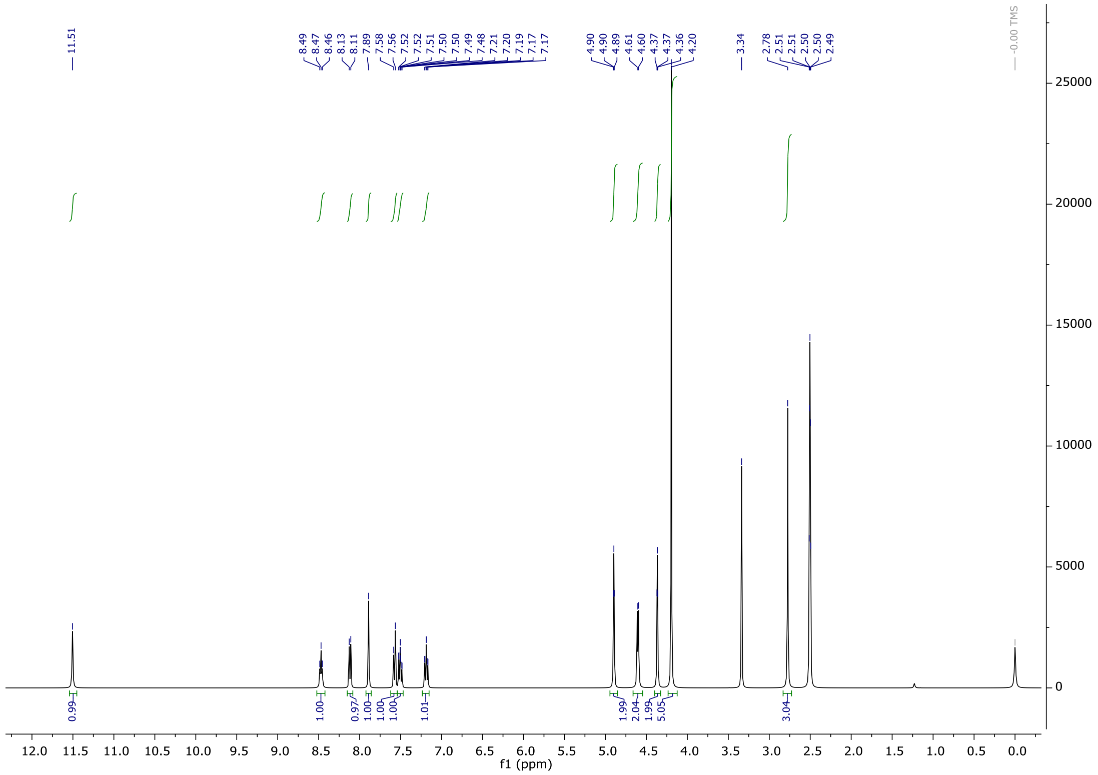
11.51
8.49 8.47 8.46 8.13 8.11 7.89 7.58 7.56 7.52 7.52 7.51 7.50 7.50 7.49 7.48 7.21 7.20 7.19 7.17 7.17
4.90 4.90 4.89 4.61 4.60 4.37 4.37 4.36 4.20
3.34
2.78 2.51 2.51 2.50 2.50 2.49
0.00 TMS
0.99 1.00 0.97 1.00 1.00 1.00 1.01 1.99 2.04 1.99 5.05 3.04
f1 (ppm)

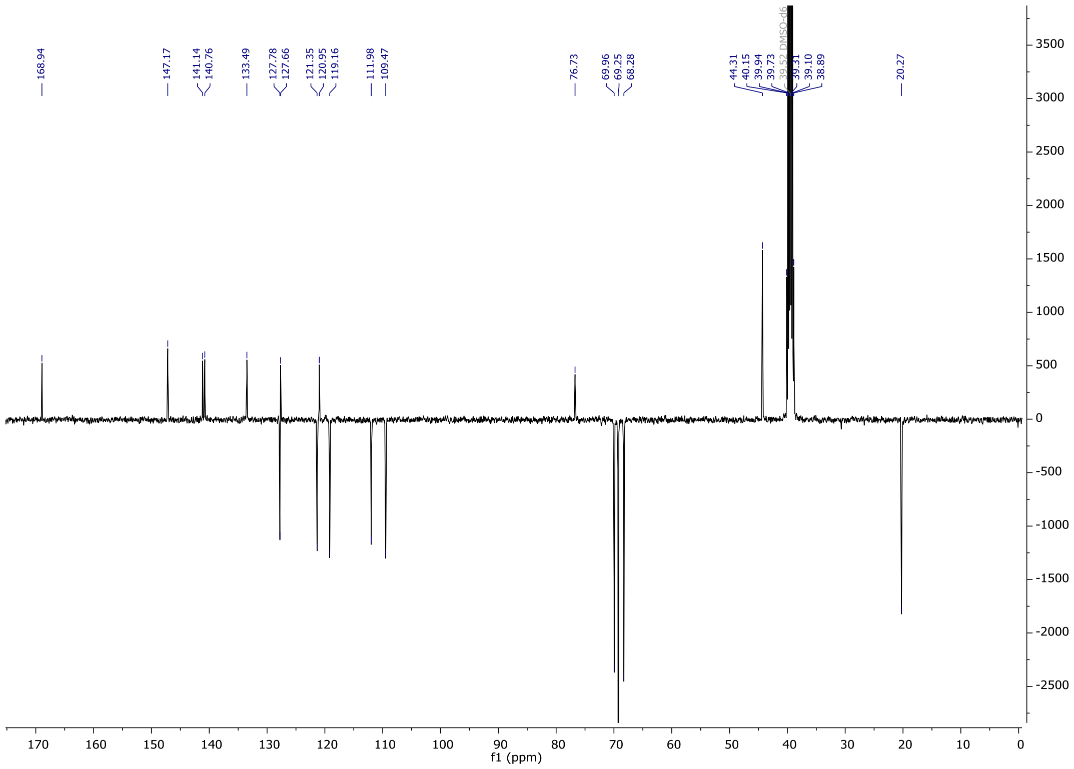
168.94
147.17
141.14
140.76
133.49
127.78
127.66
121.35
120.95
119.16
111.98
109.47
76.73
69.96
69.25
68.28
44.31
40.15
39.94
39.73
39.52 DMSO-d6
39.31
39.10
38.89
20.27
f1 (ppm)

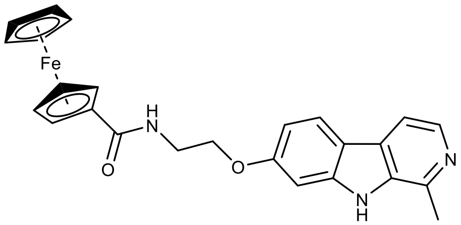
Fe
H
N
O
O
N
N
H

32

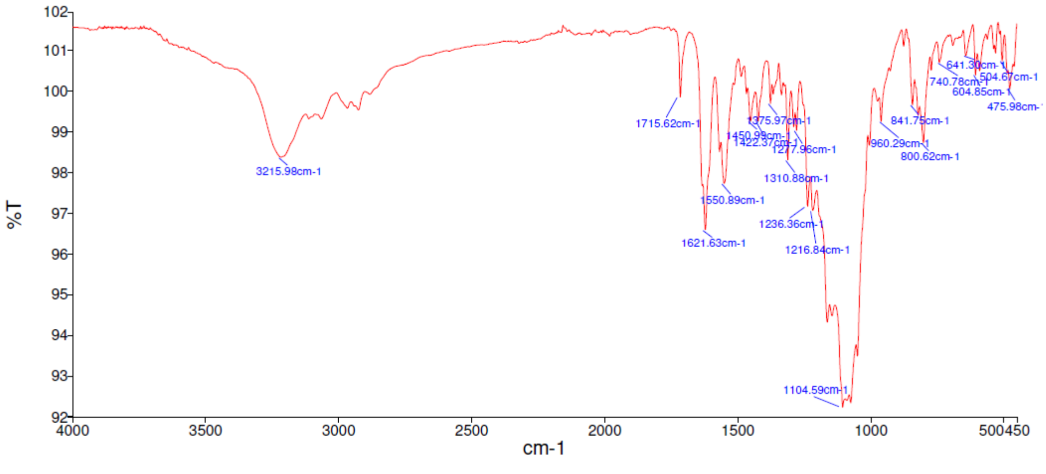
%T
cm-1
3215.98cm-1
1715.62cm-1
1621.63cm-1
1550.89cm-1
1450.99cm-1
1422.37cm-1
1375.97cm-1
1310.88cm-1
1277.96cm-1
1236.36cm-1
1216.84cm-1
1104.59cm-1
960.29cm-1
841.75cm-1
800.62cm-1
740.78cm-1
641.30cm-1
604.85cm-1
504.67cm-1

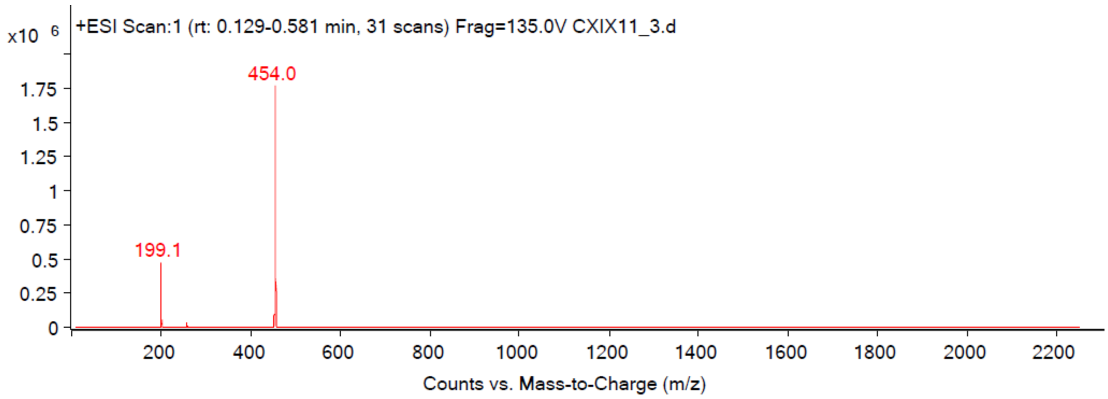
+ESI Scan:1 (rt: 0.129-0.581 min, 31 scans) Frag=135.0V CXIX11_3.d
x10 6
454.0
199.1
Counts vs. Mass-to-Charge (m/z)

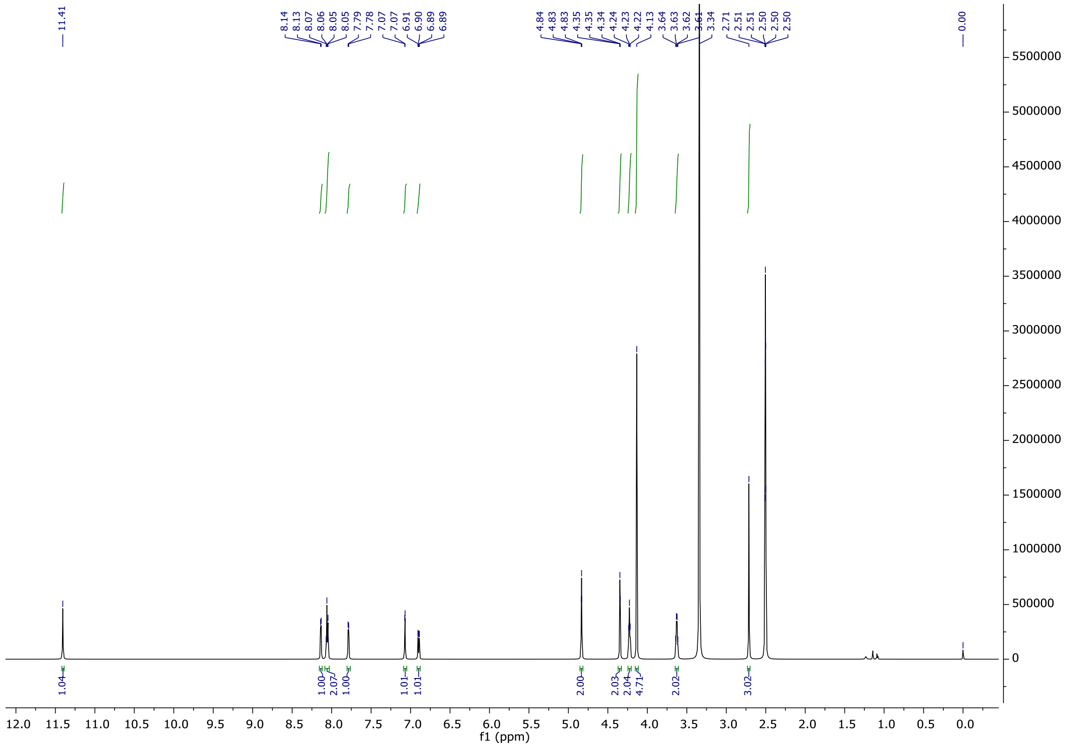
11.41
8.14 8.13 8.07 8.06 8.05 8.05 7.79 7.78 7.07 7.07 6.91 6.90 6.89 6.89
4.84 4.83 4.83 4.35 4.35 4.34 4.24 4.23 4.22 4.13 3.64 3.63 3.62 3.61 3.34 2.71 2.51 2.51 2.50 2.50 2.50
0.00
1.04 1.00 2.07 1.00 1.01 1.01 2.00 2.03 2.04 4.71 2.02 3.02
f1 (ppm)

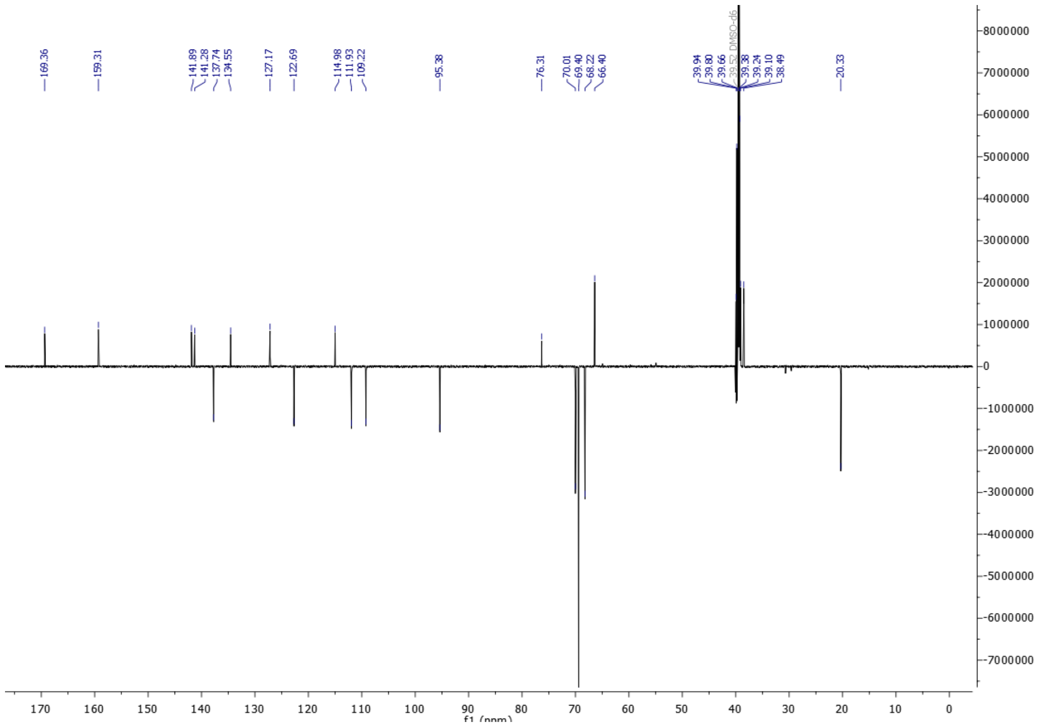
169.36
159.31
141.89
141.28
137.74
134.55
127.17
122.69
114.98
111.93
109.22
95.38
76.31
70.01
69.40
68.22
66.40
39.94
39.80
39.66
39.52 DMSO-d6
39.38
39.24
39.10
38.49
20.33
f1 (ppm)

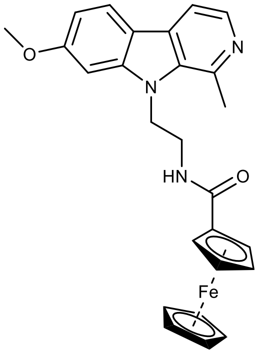
O
N
N
HN
O
Fe

33

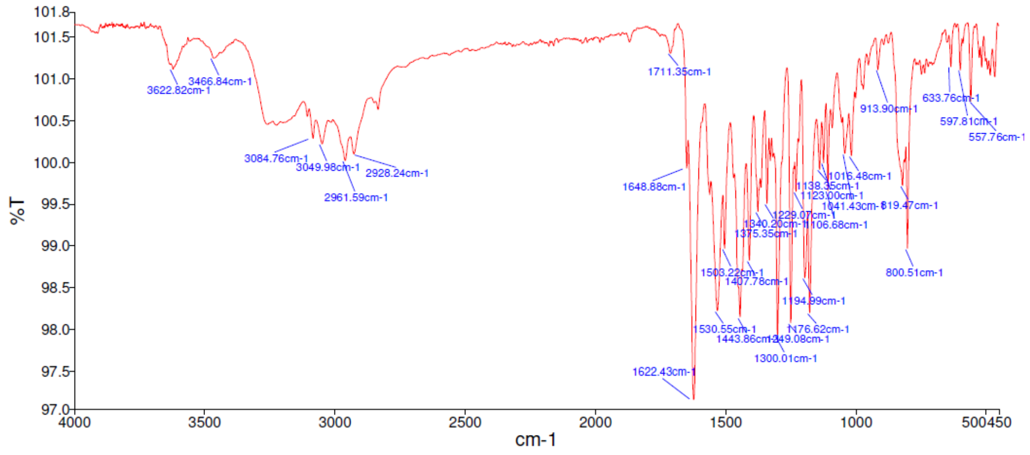
%T
cm-1
3622.82cm-1
3466.84cm-1
3084.76cm-1
3049.98cm-1
2961.59cm-1
2928.24cm-1
1711.35cm-1
1648.88cm-1
1622.43cm-1
800.51cm-1
913.90cm-1
633.76cm-1
597.81cm-1
557.76cm-1

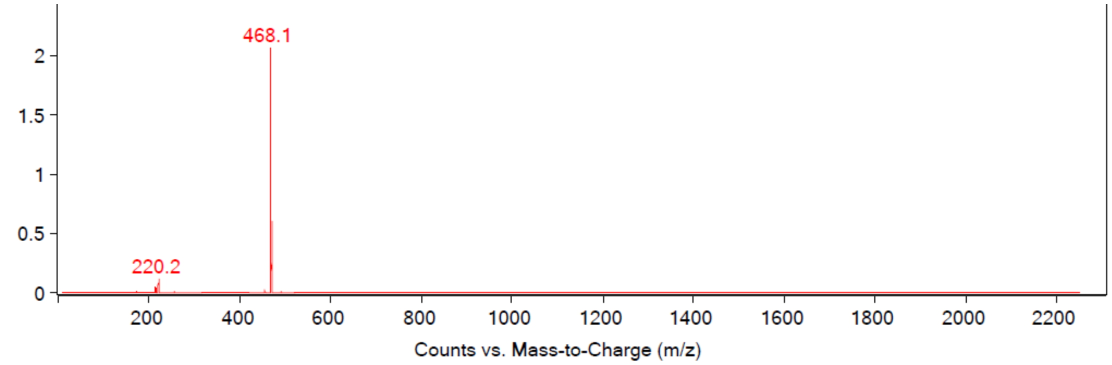

468.1
220.2
Counts vs. Mass-to-Charge (m/z)

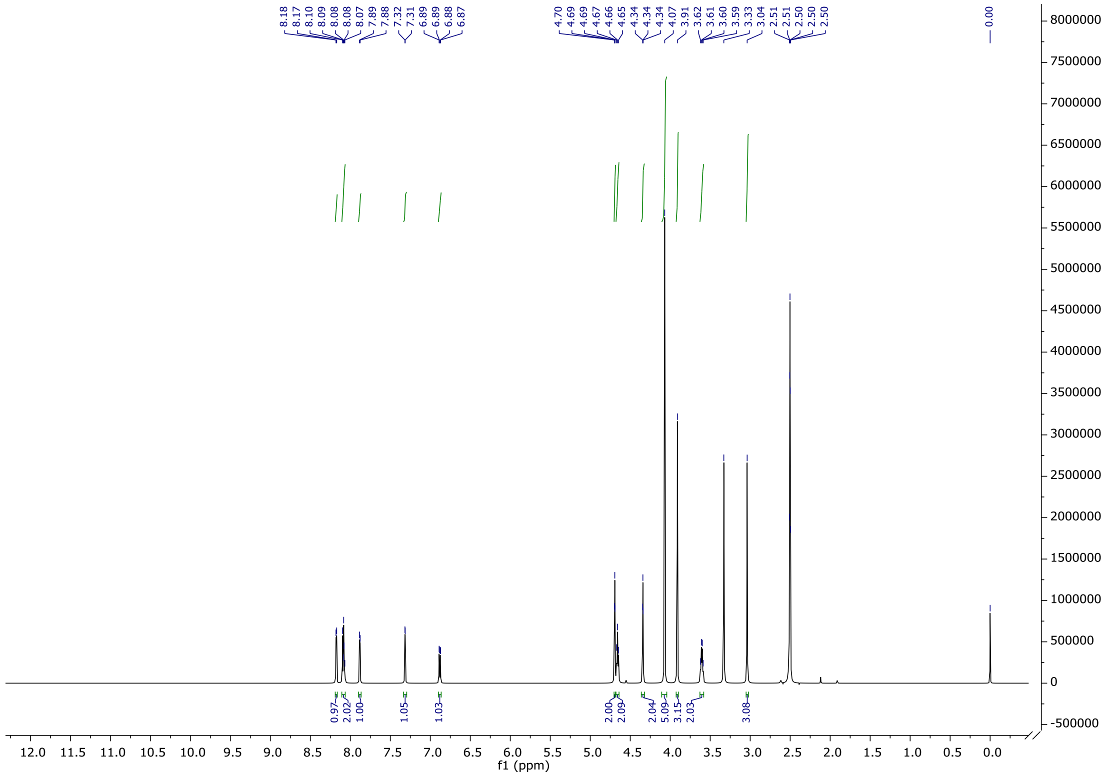

8.18
8.17
8.10
8.09
8.08
8.08
8.07
7.89
7.88
7.32
7.31
6.89
6.88
6.87
4.70
4.69
4.67
4.66
4.65
4.34
4.34
4.34
4.07
3.91
3.62
3.61
3.60
3.59
3.33
3.04
2.51
2.51
2.50
2.50
2.50
0.00
0.97
2.02
1.00
1.05
1.03
2.00
2.09
2.04
5.09
3.15
2.03
3.08
f1 (ppm)

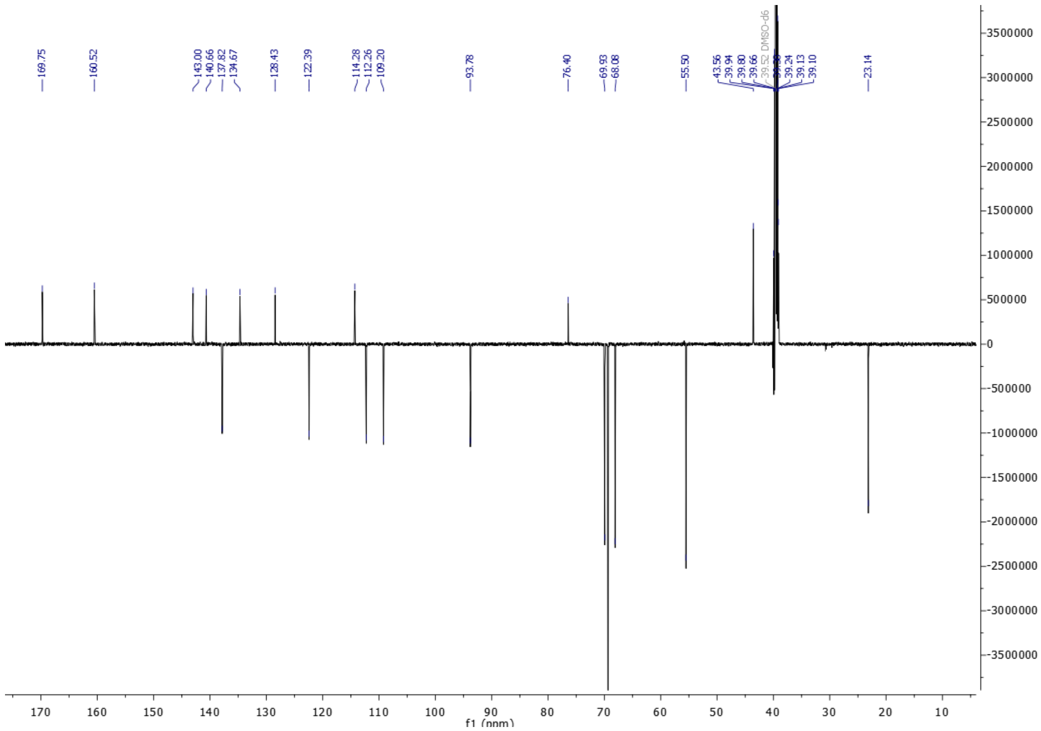

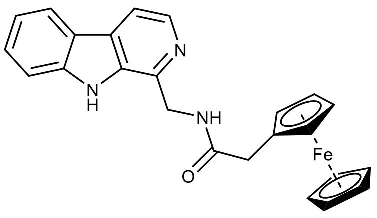

34

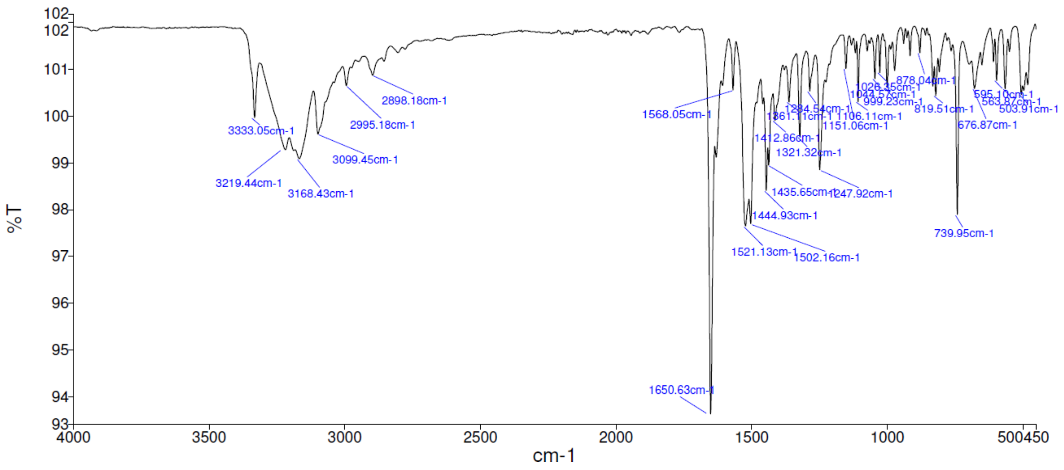

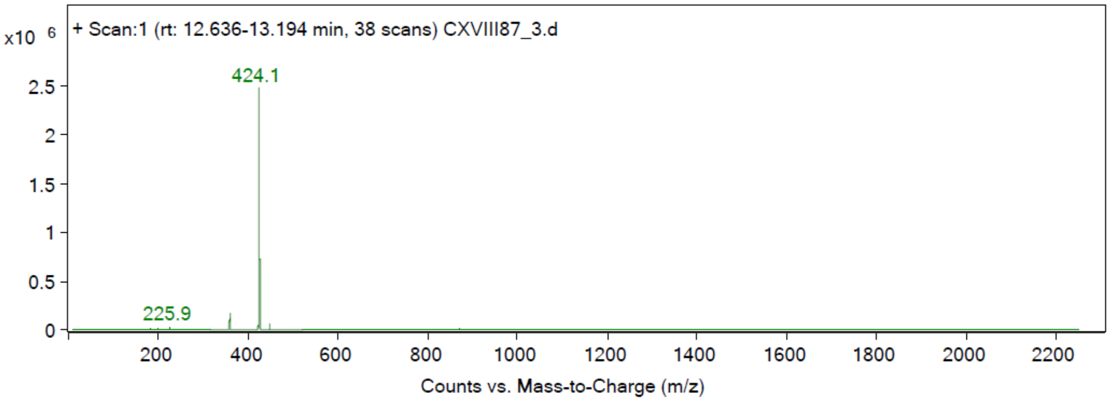

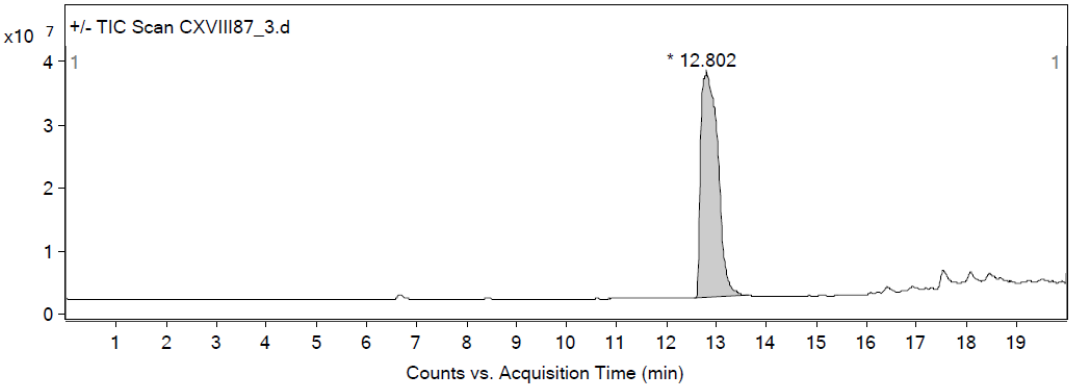

Integration Peak List

Peak	Start	RT	End	Height	Area	Area %
1	12.546	12.802	13.631	36091192.23	844377614.54	100

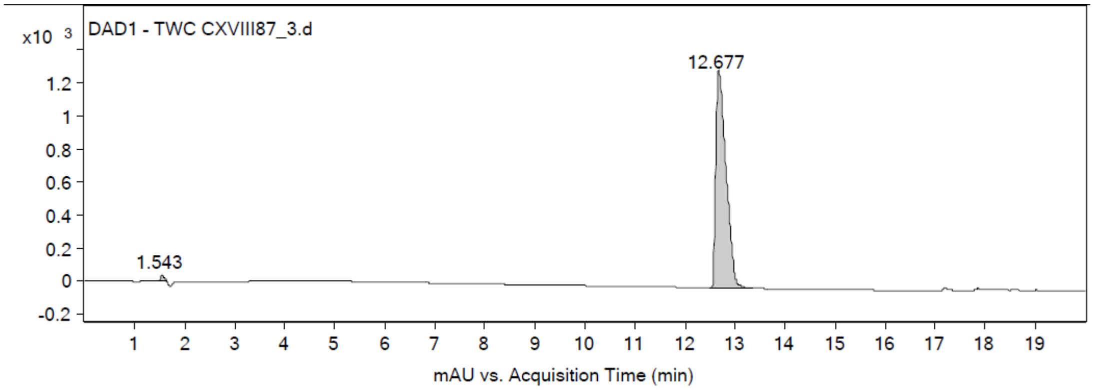

Integration Peak List

Peak	Start	RT	End	Height	Area	Area %
1	1.45	1.543	1.639	38.73	190.72	1.01
2	12.517	12.677	13.358	1319.93	18842.47	100

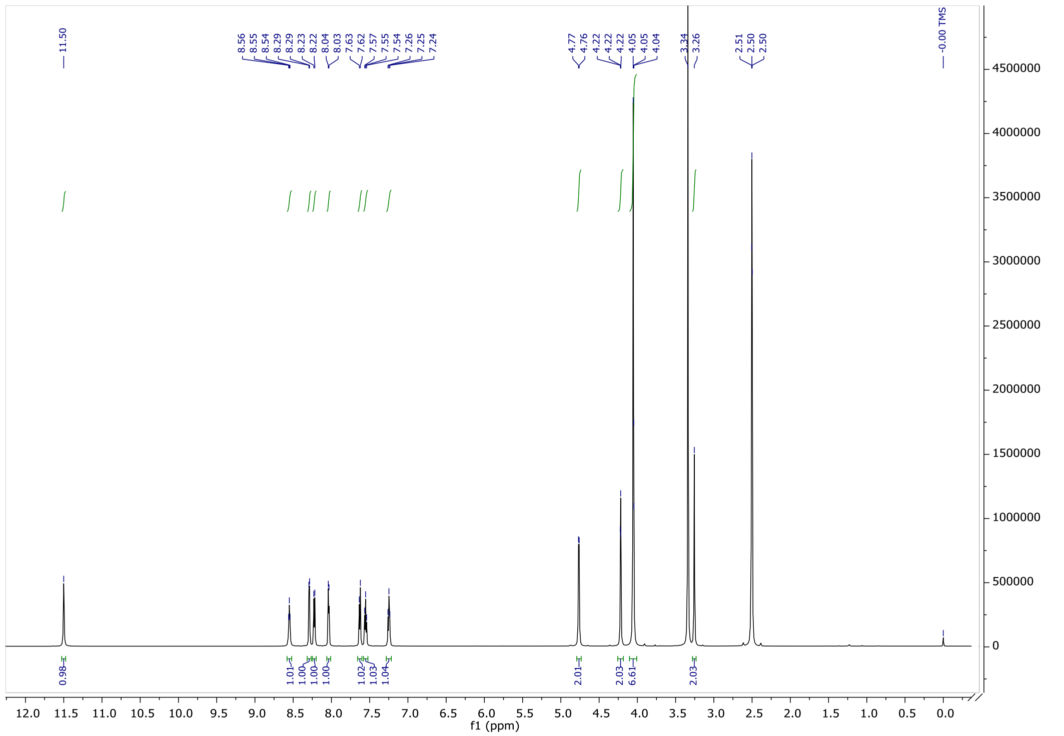

11.50
8.56
8.55
8.54
8.29
8.29
8.23
8.22
8.04
8.03
7.63
7.62
7.57
7.55
7.54
7.26
7.25
7.24
4.77
4.76
4.22
4.22
4.22
4.22
4.05
4.05
4.04
3.34
3.26
2.51
2.50
2.50
-0.00 TMS
0.98
1.01
1.00
1.00
1.00
1.02
1.03
1.04
2.01
2.03
6.61
2.03
f1 (ppm)

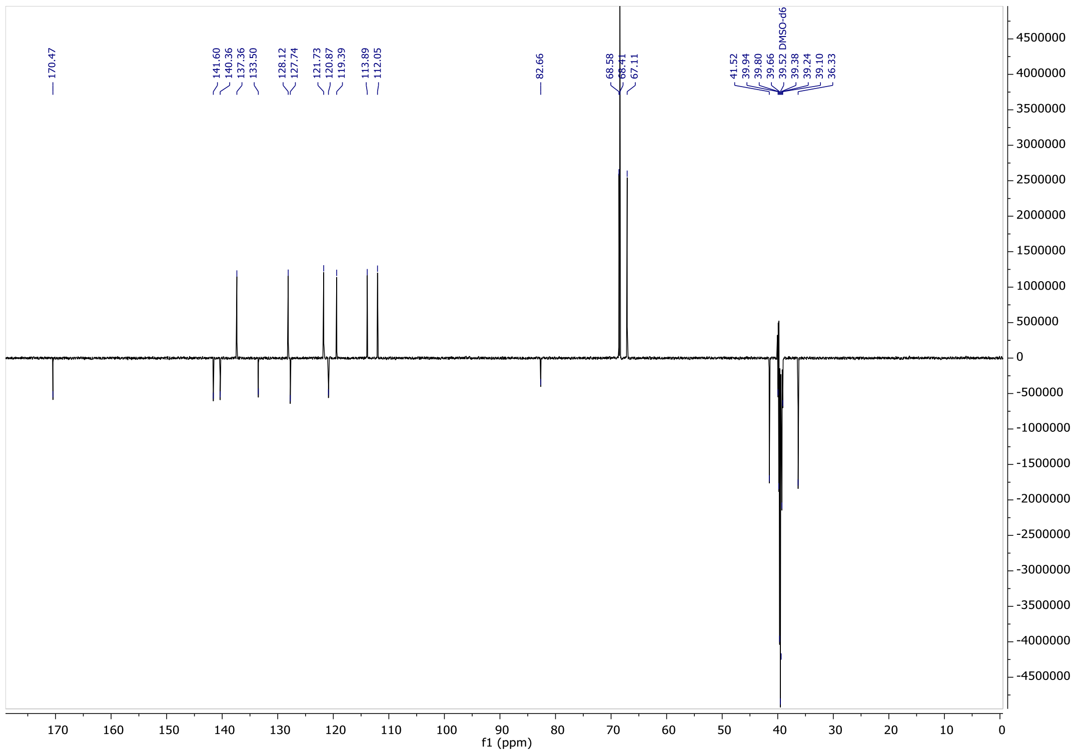

170.47
141.60
140.36
137.36
133.50
128.12
127.74
121.73
120.87
119.39
113.89
112.05
82.66
68.58
68.41
67.11
41.52
39.94
39.80
39.66
39.52 DMSO-d6
39.38
39.24
39.10
36.33
f1 (ppm)

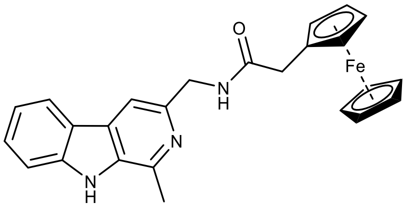

35

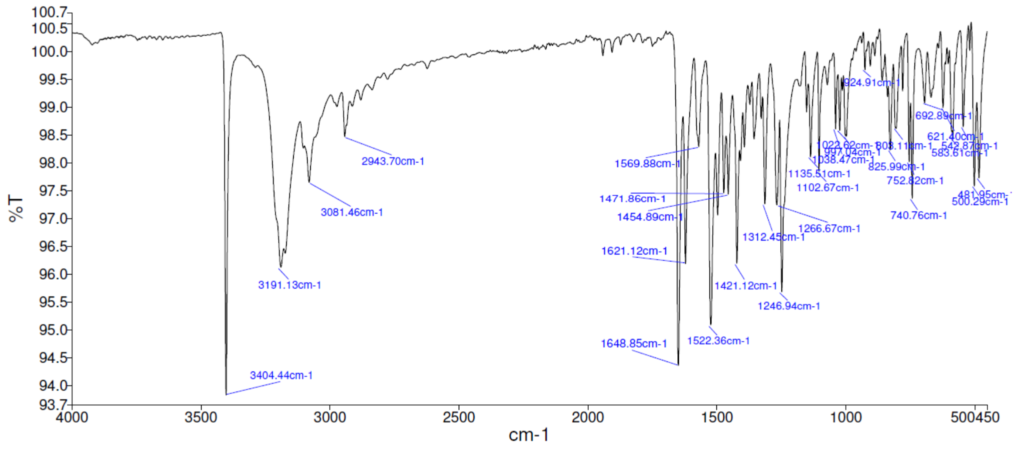

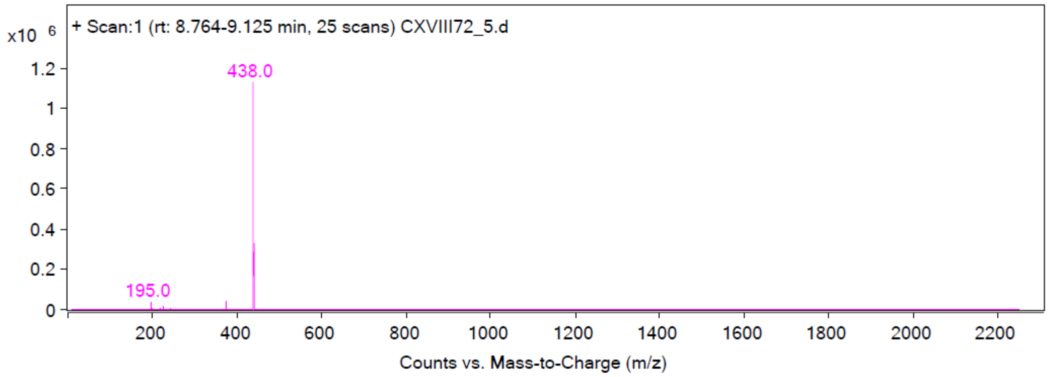

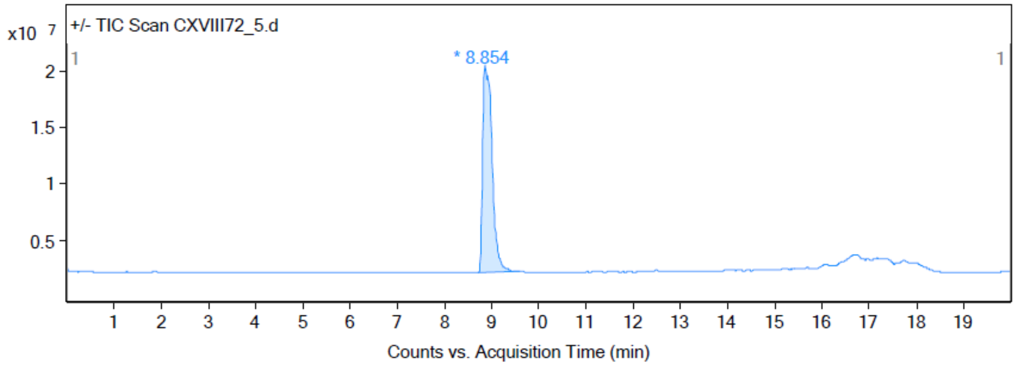

Integration Peak List

Peak	Start	RT	End	Height	Area	Area %
1	8.703	8.854	9.577	18358298.28	248380233.19	100

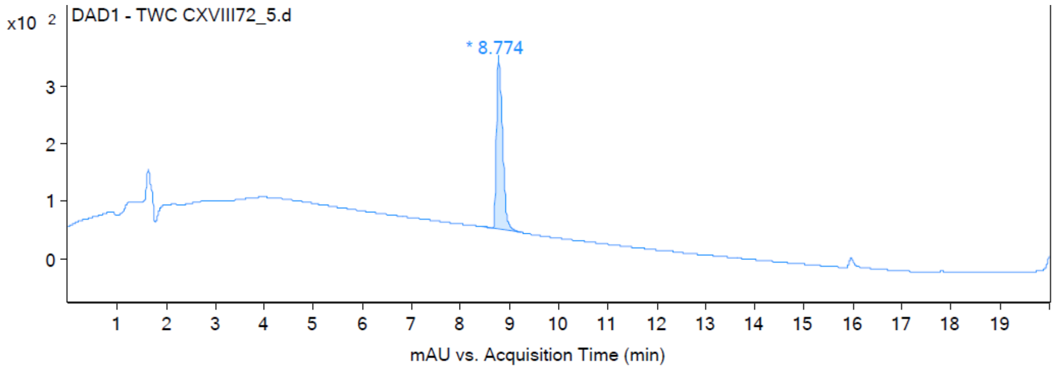

Integration Peak List

Peak	Start	RT	End	Height	Area	Area %
1	8.42	8.774	9.234	301.5	2556	100

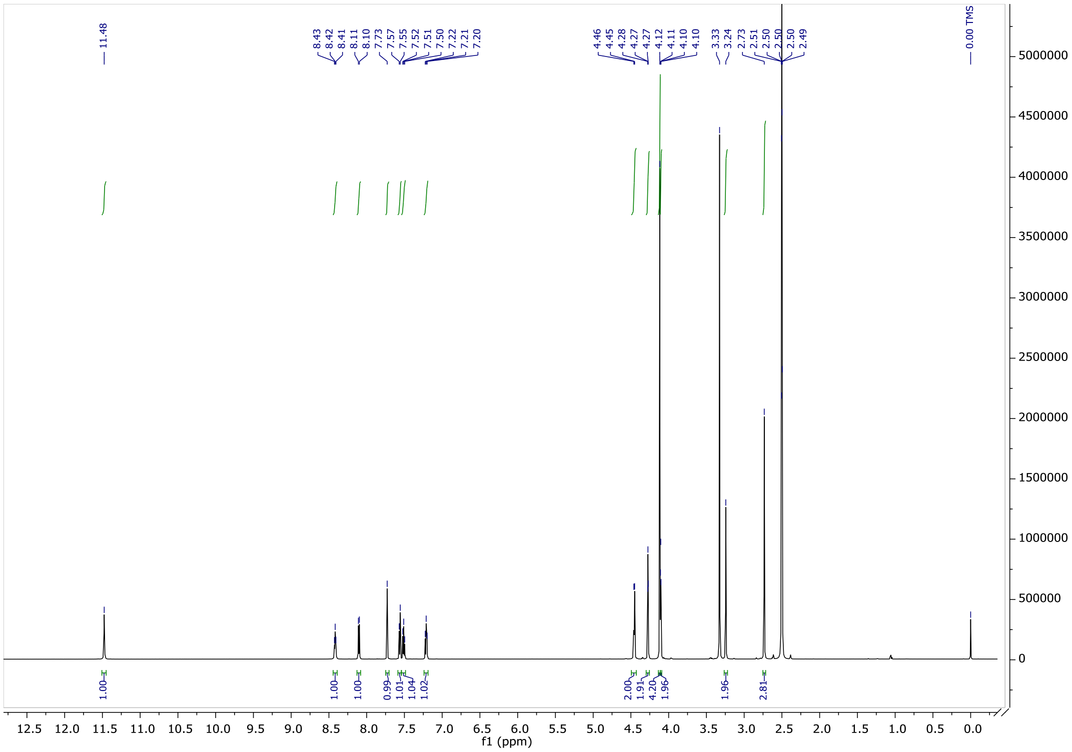

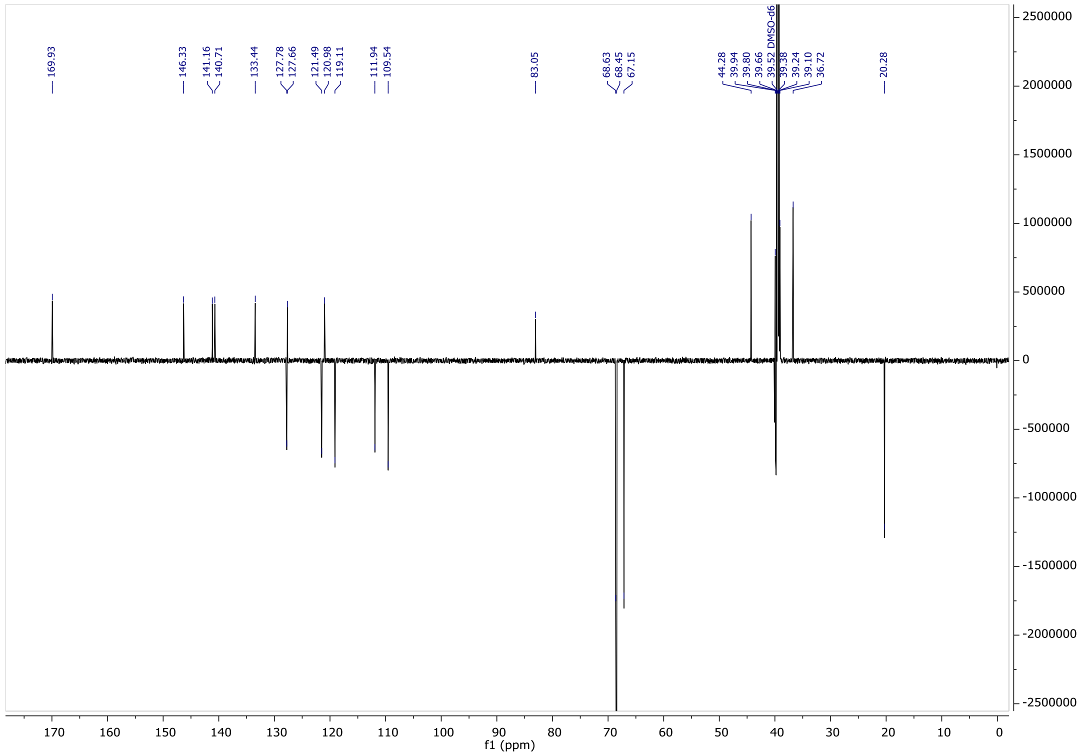
169.93
146.33
141.16
140.71
133.44
127.78
127.66
121.49
120.98
119.11
111.94
109.54
83.05
68.63
68.45
67.15
44.28
39.94
39.80
39.66
39.52 DMSO-d6
39.38
39.24
39.10
36.72
20.28
f1 (ppm)

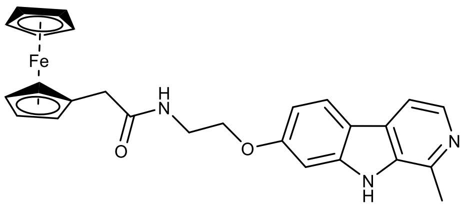
Fe
H
N
O
O
N
N
H

36

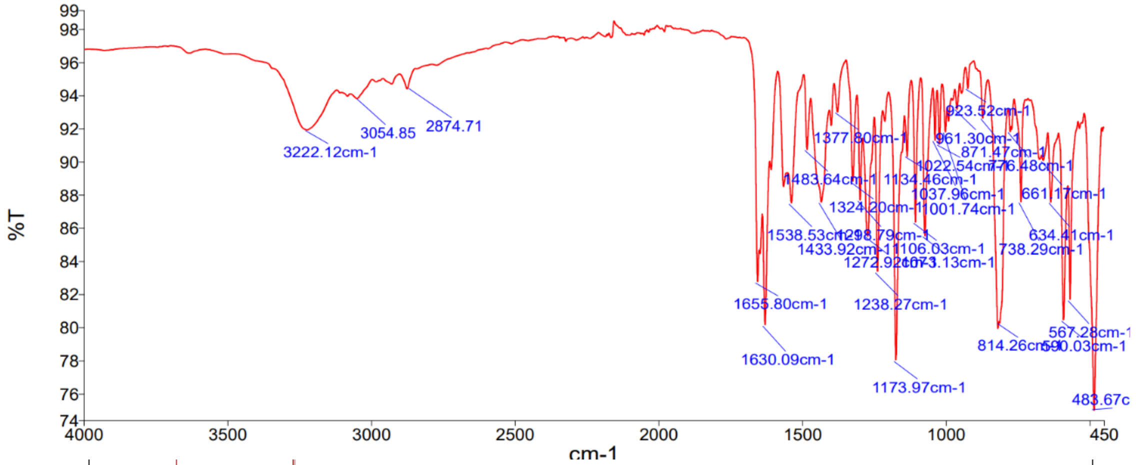
%T
cm-1
3222.12cm-1
3054.85
2874.71
1655.80cm-1
1630.09cm-1
1173.97cm-1
1238.27cm-1
814.26cm-1
483.67c

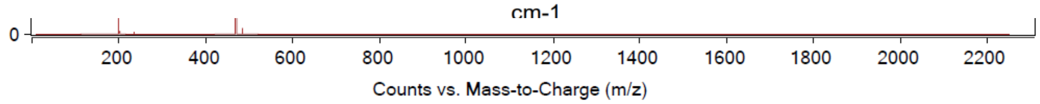
Counts vs. Mass-to-Charge (m/z)

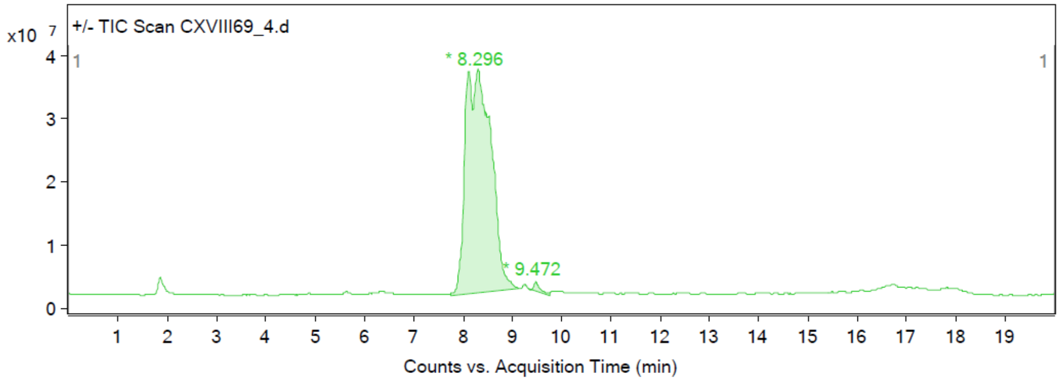

Integration Peak List

Peak	Start	RT	End	Height	Area	Area %
1	7.739	8.296	9.125	35456743.85	1278789928.72	100
2	9.336	9.472	9.773	1420821.54	13300609.58	1.04

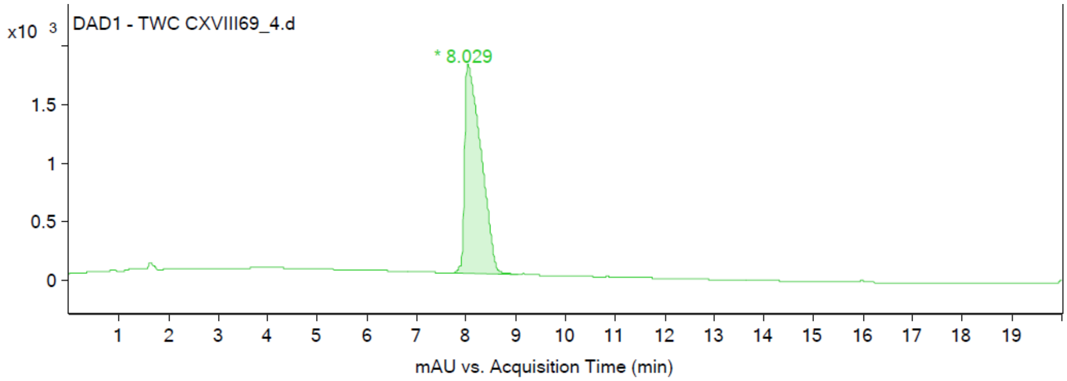

Integration Peak List

Peak	Start	RT	End	Height	Area	Area %
1	7.722	8.029	9.029	1782.73	41115.7	100

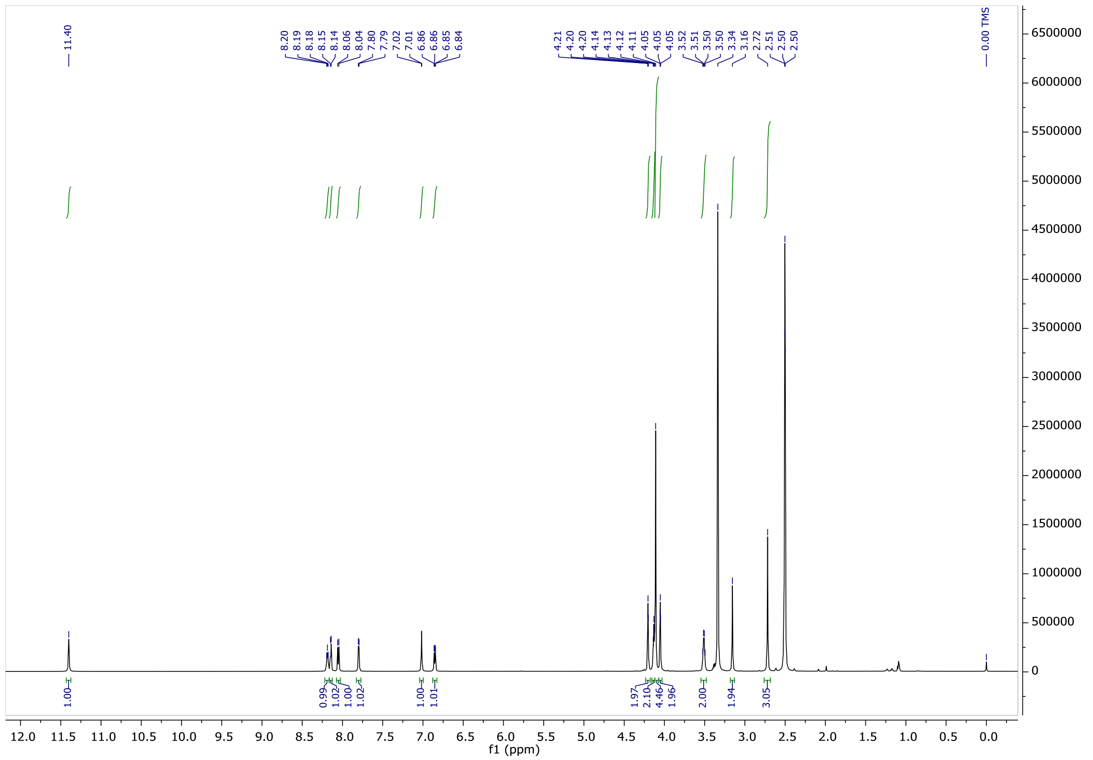
11.40
8.20 8.19 8.18 8.15 8.14 8.06 8.04 7.80 7.79 7.02 7.01 6.86 6.86 6.85 6.84
4.21 4.20 4.20 4.14 4.13 4.12 4.11 4.05 4.05 3.52 3.51 3.50 3.50 3.34 3.16 2.72 2.51 2.50 2.50
0.00 TMS
1.00 0.99 1.02 1.00 1.02 1.00 1.01 1.97 2.10 4.46 1.96 2.00 1.94 3.05
f1 (ppm)

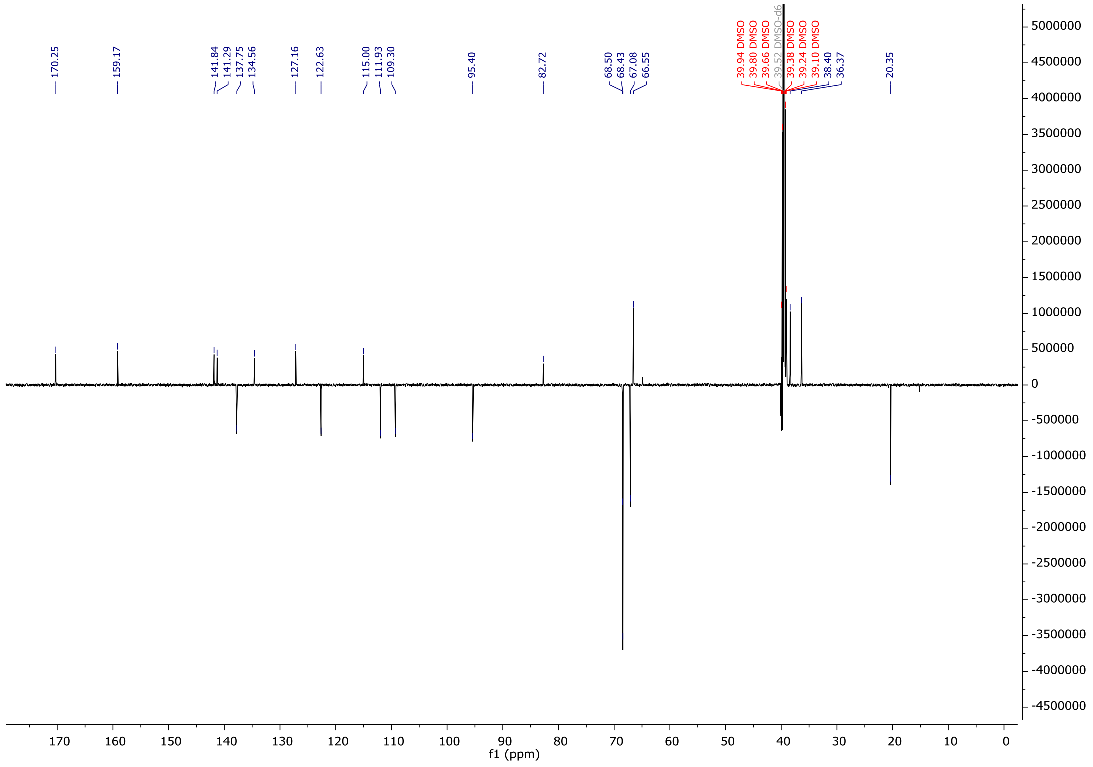
170.25 159.17 141.84 141.29 137.75 134.56 127.16 122.63 115.00 111.93 109.30 95.40 82.72 68.50 68.43 67.08 66.55
39.94 DMSO 39.80 DMSO 39.66 DMSO 39.52 DMSO-d6 39.38 DMSO 39.24 DMSO 39.10 DMSO 38.40 36.37 20.35
f1 (ppm)

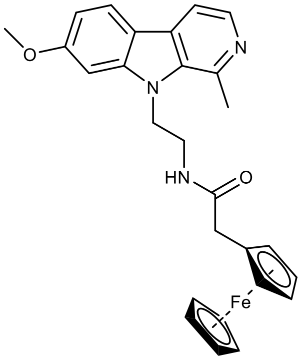
O
N
N
HN
O
Fe

37

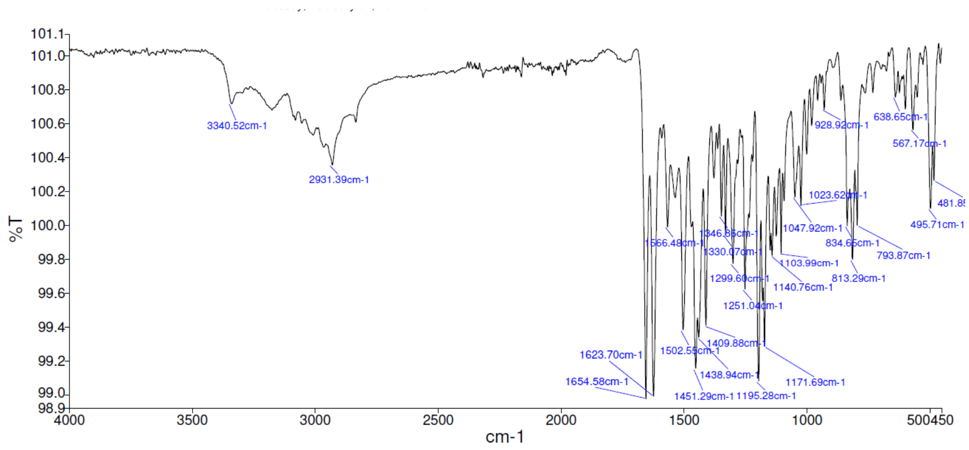
%T
cm-1
3340.52cm-1
2931.39cm-1
1654.58cm-1
1623.70cm-1
1502.55cm-1
1451.29cm-1
1438.94cm-1
1409.88cm-1
1195.28cm-1
1171.69cm-1
1251.04cm-1
1299.60cm-1
1140.76cm-1
1103.99cm-1
1047.92cm-1
1023.62cm-1
928.92cm-1
834.65cm-1
813.29cm-1
793.87cm-1
638.65cm-1
567.17cm-1
495.71cm-1

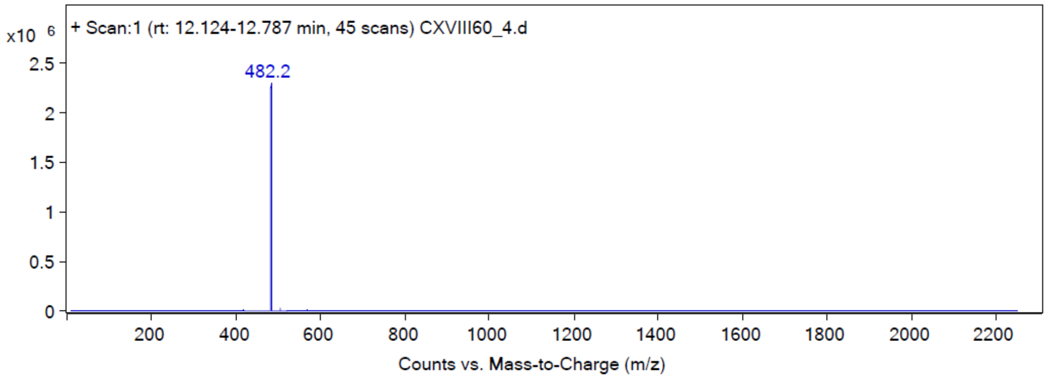
+ Scan:1 (rt: 12.124-12.787 min, 45 scans) CXVIII60_4.d
x10 6
482.2
Counts vs. Mass-to-Charge (m/z)

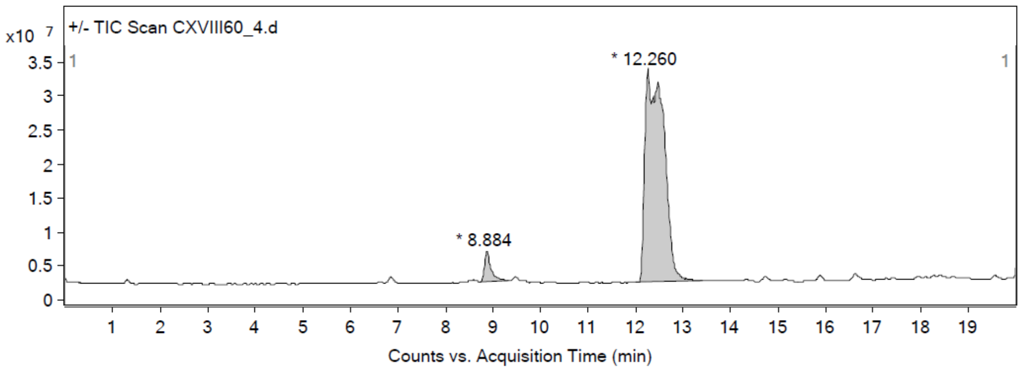

Integration Peak List

Peak	Start	RT	End	Height	Area	Area %
1	8.718	8.884	9.336	4479840.71	44307179.08	5.08
2	12.019	12.26	13.39	31597336.17	871523549.01	100

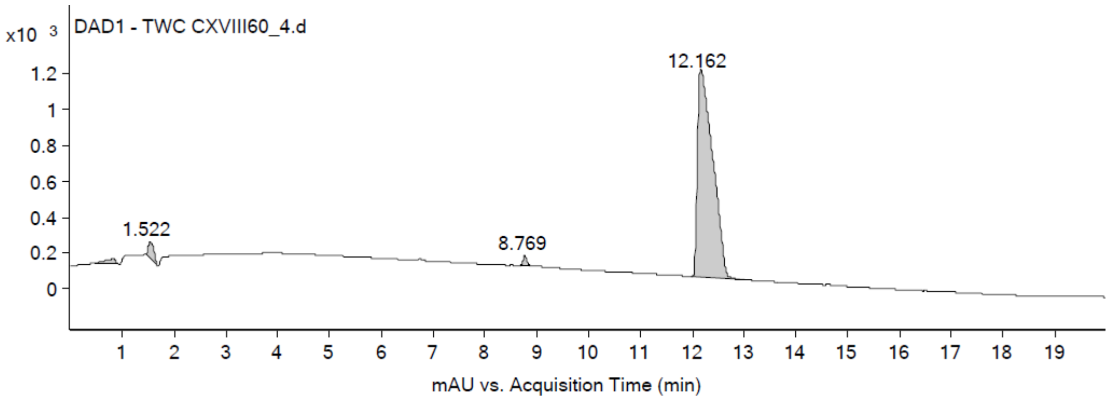

Integration Peak List

Peak	Start	RT	End	Height	Area	Area %
1	0.475	0.822	0.909	24.56	351.74	1.49
2	1.455	1.522	1.662	87.27	731.86	3.1
3	8.694	8.769	8.875	58.9	287.3	1.22
4	11.975	12.162	13.029	1153.28	23574.59	100

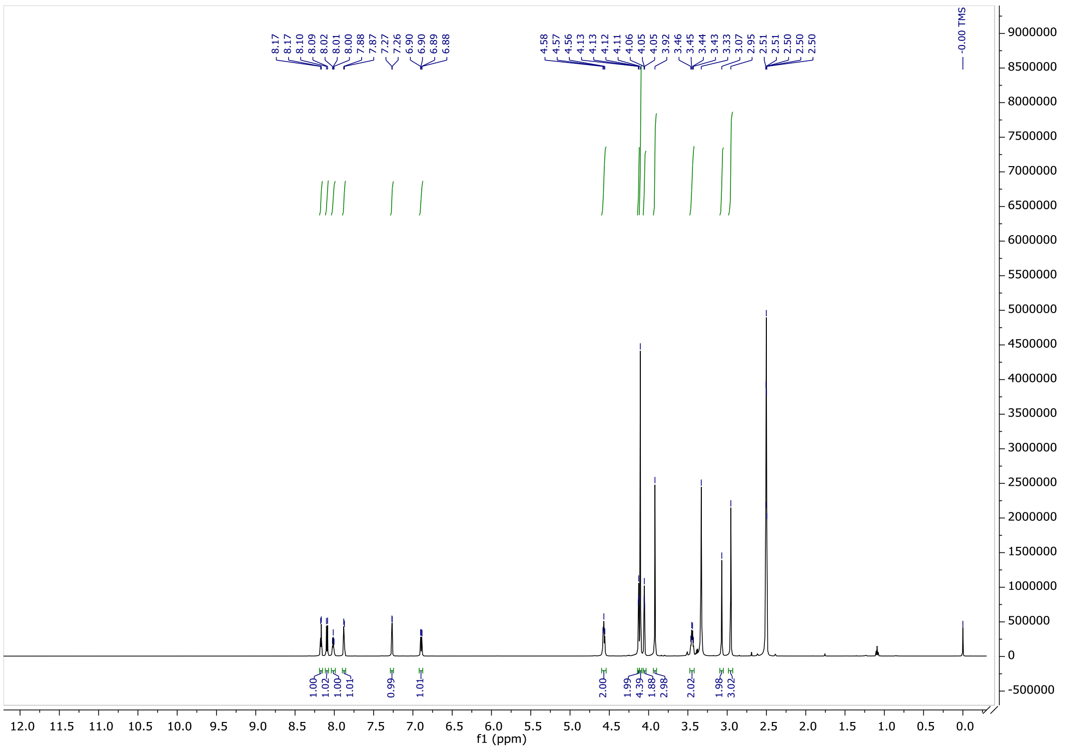
8.17
8.17
8.10
8.09
8.02
8.01
8.00
7.88
7.87
7.27
7.26
6.90
6.89
6.88
4.58
4.57
4.56
4.13
4.13
4.12
4.11
4.06
4.05
4.05
3.92
3.46
3.45
3.44
3.43
3.33
3.07
2.95
2.51
2.51
2.50
2.50
2.50
-0.00 TMS
1.00
1.02
1.00
1.01
0.99
1.01
2.00
1.99
4.39
1.88
2.98
2.02
1.98
3.02
f1 (ppm)

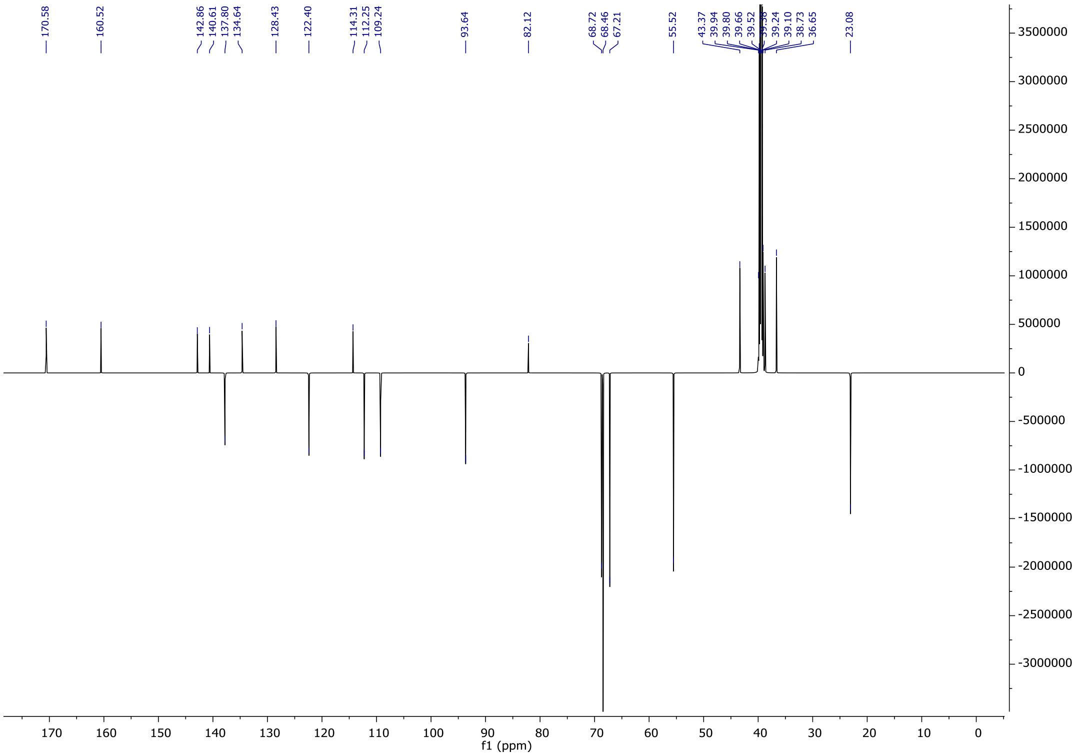
170.58
160.52
142.86
140.61
137.80
134.64
128.43
122.40
114.31
112.25
109.24
93.64
82.12
68.72
68.46
67.21
55.52
43.37
39.94
39.80
39.66
39.52
39.38
39.24
39.10
38.73
36.65
23.08
f1 (ppm)

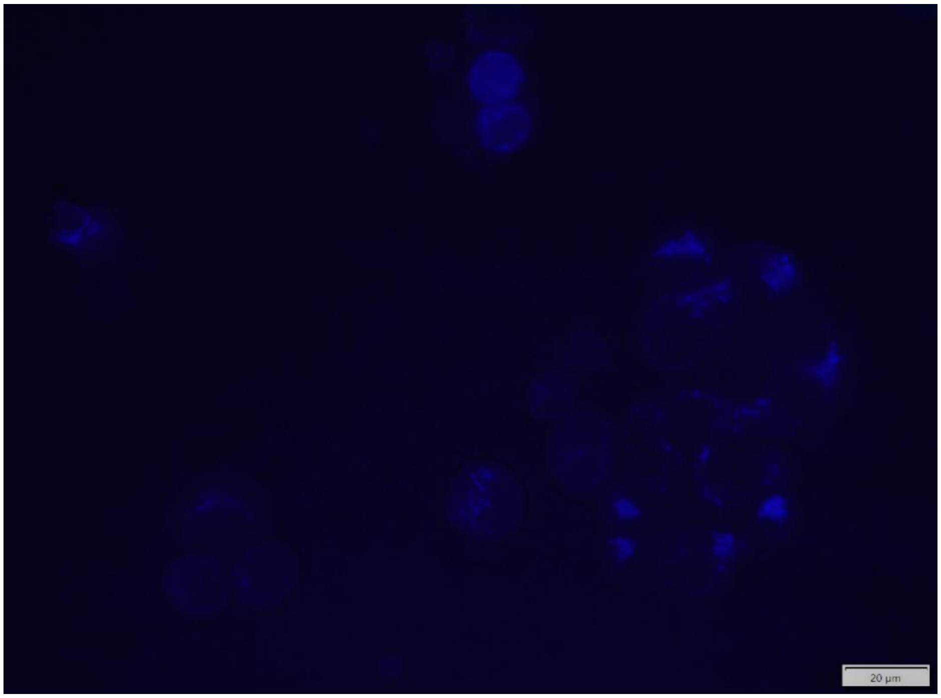

Figure S1. Fluorescent microscopy image (400 × magnification) of MCF-7 cells incubated with 5 μM concentration of compound **23** for 30 min. The scale bar is 20 μm.
